# Analysis of large mutations in *BARD1* in patients with breast and/or ovarian cancer: the Polish population as an example

**DOI:** 10.1038/srep10424

**Published:** 2015-05-21

**Authors:** Katarzyna Klonowska, Magdalena Ratajska, Karol Czubak, Alina Kuzniacka, Izabela Brozek, Magdalena Koczkowska, Marcin Sniadecki, Jaroslaw Debniak, Dariusz Wydra, Magdalena Balut, Maciej Stukan, Agnieszka Zmienko, Beata Nowakowska, Irmgard Irminger-Finger, Janusz Limon, Piotr Kozlowski

**Affiliations:** 1 European Centre for Bioinformatics and Genomics, Institute of Bioorganic Chemistry, Polish Academy of Sciences, Noskowskiego 12/14, 61-704 Poznan, Poland; 2 Medical University of Gdansk, Marii Sklodowskiej-Curie 3a, 80-211 Gdansk, Poland; 3 Gdynia Oncology Centre, Powstania Styczniowego 1, 81-519 Gdynia, Poland; 4 Poznan University of Technology, Pl. Marii Sklodowskiej-Curie 5, 60-965 Poznan, Poland; 5 Department of Medical Genetics, Institute of Mother and Child, Kasprzaka 17A, 01-211 Warsaw, Poland; 6 University Hospitals of Geneva, HUG, Geneva, Switzerland

## Abstract

Only approximately 50% of all familial breast cancers can be explained by known genetic factors, including mutations in *BRCA1* and *BRCA2*. One of the most extensively studied candidates for breast and/or ovarian cancer susceptibility is *BARD1*. Although it was suggested that large mutations may contribute substantially to the deleterious variants of *BARD1*, no systematic study of the large mutations in *BARD1* has been performed. To further elucidate the role of large mutations in *BARD1*, we designed a multiplex ligation-dependent probe amplification (MLPA) assay and performed an analysis of 504 women with a familial breast and/or ovarian cancer and 313 patients with ovarian cancer. The investigation did not reveal any large mutations in the *BARD1* gene. Although the analysis was not focused on identification of small mutations, we detected seven deleterious or potentially deleterious point mutations, which contribute substantially to the total number of *BARD1* mutations detected so far. In conclusion, although we cannot exclude the presence of large mutations in *BARD1*, our study indicates that such mutations do not contribute substantially to the risk of breast and/or ovarian cancer. However, it has to be noted that our results may be specific to the Polish population.

Five to ten percent of all breast cancer (MIM#114480) cases are inherited and consequently aggregate in families. Hereditary breast cancer, on average, is diagnosed in a young age and/or co-occurs with ovarian cancer (MIM#167000). It is estimated that germline mutations affecting the highly susceptible *BRCA1* (MIM*113705) and *BRCA2* (MIM*600185) genes explain 16–40% of all familial breast cancer cases^1^. Moreover, highly penetrant mutations in genes such as *TP53* (MIM*191170), *STK11* (MIM*602216), *CDH1* (MIM*192090), and *PTEN* (MIM + 601728) are associated with various hereditary cancer syndromes and account for approximately 1% of all breast cancer cases that aggregate in families^2^. Another ~5% of familial breast cancers may be explained by mutations in moderately susceptible genes such as *ATM* (MIM*607585), *CHEK2* (MIM + 604373), *NBN* (MIM*602667), *RAD50* (MIM*604040), *RAD51B* (MIM*602948), and *RAD51D* (MIM*602954) and the genes implicated in Fanconi anemia. Finally, a significant proportion of breast cancer aggregation may result from the cooperative effect of common polymorphisms (primarily SNPs) or by their interaction with highly susceptible genes. Recently, a large, cooperative genome-wide association study identified the association of 67 new and previously reported SNPs with breast cancer^3^. It was estimated that these SNPs cumulatively explain 14% of the familial heritability of breast cancer, and a further 9% may be explained by yet unknown common SNPs^3^. Altogether, only approximately 50% of all familial breast cancer cases can be explained by known genetic factors^1^,^4^. Therefore, the identification of additional cancer-susceptibility genes is highly sought-after^1^,^2^,^4^,^5^.

Among the candidate breast and/or ovarian cancer susceptibility genes are those encoding proteins that interact with BRCA1/2 in DNA damage response and other tumor suppressor pathways^2^,^4^. One such gene that has been intensively studied is *BARD1* (BRCA1 associated RING domain 1; MIM #601593). *BARD1* is composed of 11 exons spread out over an 85-kb region at 2q35 and encodes a 777-amino-acid protein that shares both structural and functional similarities with BRCA1. Both proteins possess an amino-terminal RING-finger motif that facilitates BARD1/BRCA1 heterodimer formation. This in turn stabilize both proteins and is essential for the expression of the tumor suppressor functions of BRCA1^6^. It has been shown that missense mutations in the BRCA1 RING-finger domain that hamper heterodimer formation are highly penetrant deleterious mutations.

The analysis of *BARD1* in subjects with increased genetic risk of breast and/or ovarian cancer led to the identification of dozens of sequence alterations, including definitively damaging, frameshift and nonsense mutations^7^,^8^,^9^,^10^,^11^,^12^,^13^,^14^,^15^,^16^,^17^,^18^,^19^,^20^,^21^,^22^,^23^,^24^,^25^,^26^,^27^,^28^,^29^,^30^. Detected mutations are distributed over almost entire sequence of *BARD1* and no strong hot-spot mutation or hot-spot region was identified so far. The exception may be c.1670G >C (p.Cys557Ser) which is relatively frequent in European populations however its role in breast cancer predisposition is equivocal^7^,^10^,^11^,^12^,^13^,^15^,^16^,^17^,^19^,^22^,^23^,^25^. Depending on a type of tested samples and a criteria of mutation definition, the *BARD1* mutation rate (ratio of the number of mutations and the number of analyzed patients) varies between 2.8% and 6.1%^10^,^19^,^23^. Recently, highly deleterious *BARD1* mutations were also detected with the use of exome sequencing of cancer predisposing genes^21^,^26^,^27^,^28^,^29^,^30^. These more objective approaches recurrently show that *BARD1* belongs to the group of the most frequently mutated genes, after *BRCA1* and *BRCA2*. For example, recent analysis of 1824 patients with triple-negative breast cancer unselected for family history led to the identification of 9 definitive *BARD1* mutations (more mutations was identified only in *BRCA1*, *BRCA2* and *PALB2*)^29^. It was shown that a number of the identified point mutations in *BARD1* co-segregate in families with cancer^10^,^19^. *BARD1* small-size mutations were also analyzed as modifiers of *BRCA1*/*BRCA2* attributed risk^31^,^32^,^33^.

However, the knowledge of large mutations in the *BARD1* gene is still very limited. One of such alterations is a deletion of 1260-bp in intron 3 of *BARD1*^24^. Additionally, a germline deletion of the entire *BARD1* gene was detected in a non-*BRCA* patient with triple-negative breast cancer. Consequently, it has been suggested that large mutations (multi-exon deletions or insertions) in *BARD1* as well as in other breast cancer susceptibility genes may substantially contribute to familial breast/ovarian cancer risk^20^,^34^,^35^. This corresponds with the previous studies demonstrating that large rearrangements may account for a substantial fraction of all of the disease-related mutations in a particular gene. Normally this fraction accounts for ~5% of all detected mutations; however, it is strongly dependent on both the gene and population background, and in some cases this number well exceeds 10%. However, to our knowledge, no systematic analysis focusing solely on large germline mutations in *BARD1* has been performed. Therefore, to unequivocally elucidate this issue, we performed a comprehensive analysis of the large mutations in *BARD1* in over 800 samples with either familial breast cancer or unselected ovarian cancer.

## Results and Discussion

**Figure 1:**
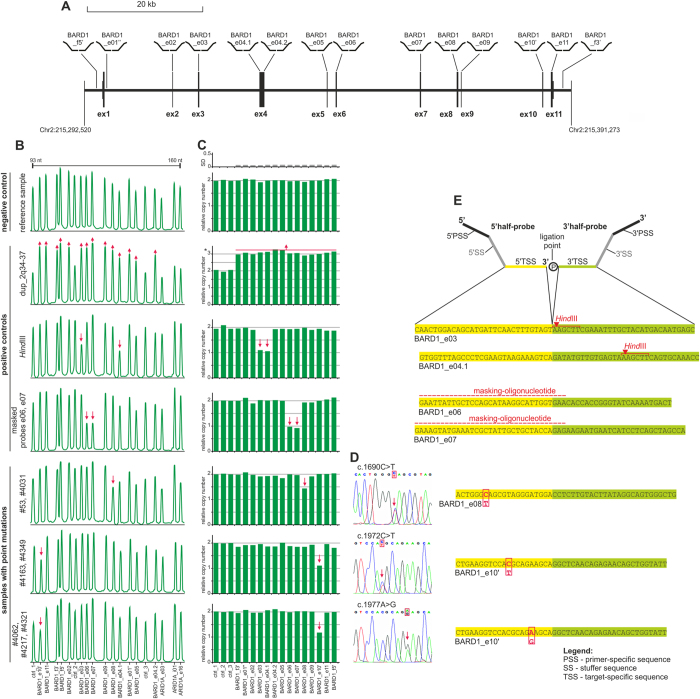
Analysis of the large mutations in *BARD1*, conducted with the use of a homemade MLPA assay. (**a**) A schematic map of the *BARD1* gene and the flanking genomic regions, with the positions and IDs of the MLPA probes indicated. The exons are presented as vertical rectangles with proportional size and spacing based on the NM_000465 *BARD1* sequence (reverse complement) retrieved from the UCSC Genome Browser (human genome reference sequence Mar 2006 NCBI36/hg18 assembly). The upper and lower rectangles correspond to the protein coding and untranslated sequences, respectively. In panels (**b–e**) there are representative results of the following control samples and samples with different point mutations (from the top): (i) the representative negative result without any mutations; (ii) the positive control sample with duplication of 2q34-37 in which *BARD1* is located; (iii) artificial positive control sample, composed of 1:1 mixture of *Hind*III digested and undigested genomic DNA sample; (iv) artificial positive control sample, generated by masking the target sequences of BARD1_e06 and BARD1_e07 probes with probe-specific masking-oligonucleotides; (v) samples #53 and #4031 with the mutation c.1690C >T in exon 8; (vi) samples #4163 and #4349 with the mutation c.1972C >T in exon 10 and (vii) samples #4062, #4217, and #4321 with the mutation c.1977A >G in exon 10. (**b**) The MLPA electropherograms of the representative MLPA results. The probe IDs are shown under the electropherograms. An arrowhead indicates a reduced signal of the MLPA probe. (**c**) The bar plots (corresponding to the electropherograms shown in panel b) representing the normalized copy number value (y-axis) of each probe (x-axis). The gray bar plot (above) indicates standard deviation values (SD; ranged between 0.066 and 0.086) of test MLPA probes, calculated based on signal variation of particular probes in each analyzed sample (except the samples with mutation). (**d**) Sequencing results of the exons showing a reduced signal in the MLPA analysis. (**e**) The target sequences of the affected probes. A schematic representation of the MLPA probe is shown above (for details see^37^,^41^). The 5’- and 3’-target sequences are indicated in yellow and green, respectively. The positions of *Hind*III sites, the masking-oligonucleotides and corresponding mutations are indicated in red.

MLPA is the method of choice for the detection of large mutations; however, commercial MLPA assays are available only for a limited number of the most intensively studied genes, and there is no assay for *BARD1*. Therefore, as a first step, we designed and generated a new MLPA assay covering all 11 exons (12 probes; one probe in each exon, two probes in exon 4) as well as the 5’- and 3’-flanking sequences (2 probes) of *BARD1* (Fig. 1). Additionally our assay was comprised of 3 control probes (located in copy-number-stable regions in chromosomes 1, 17, and 22) and 3 probes located in *ARID1A* (MIM*603024) (not used in this study). To prove the dosage-sensitivity of the designed MLPA probes, we performed an analysis of three types of positive control samples: (i) anonymous control sample with the large-scale duplication of the 2q34-37 region in which *BARD1* is located; (ii) DNA control sample, digested with *Hind*III, and mixed (1:1) with undigested sample to simulate heterozygous deletion (*Hind*III cuts target sequences of two consecutive probes, BARD1_e03 and BARD1_e04.1, and does not cut target sequences of any other MLPA probes used in the assay); and (iii) control sample, in which target sequences of two consecutive probes, BARD1_e06 and BARD1_e07, were masked by specific masking-oligonucleotides, complementary to target sequences of the selected probes (upon hybridization, the masking-oligonucleotides, prevent target recognition and subsequent ligation of MLPA probes). All the tests confirmed the dosage-sensitivity of the designed MLPA assay/probes (Fig. 1).

The designed MLPA assay was used for *BARD1* large-mutation analysis of 504 patients from families with breast and/or ovarian cancer aggregation and 313 patients with unselected ovarian cancer. The conducted analysis did not show any MLPA patterns indicating the presence of a large mutation in the analyzed samples. However, in seven samples (one unselected ovarian and six familial breast cancer cases), we observed a 28–45% reduction of the individual probe signal: two samples with a reduced signal in exon 8 and five samples with a reduced signal in exon 10 (Fig. 1). None of the rest of the analyzed samples had a MLPA probe signal reduced or increased by more than 10%.

As large heterozygous deletions lead to an approximately 50% signal reduction and commonly affect subsequent MLPA probes, we assumed that the observed reductions of the single-exon signals may have resulted from small-size sequence variants present in the target sequences of the corresponding probes. It was previously shown that such sequence variants may affect probe hybridization and/or ligation and, in consequence, lead to a relative signal reduction^36^,^37^. In all cases, the sequence analysis revealed heterozygous single nucleotide substitutions located at different distances (3–15 nucleotides) from the ligation point of the MLPA probes. In both samples that had the reduced signal in exon 8, we found the nonsense mutation c.1690C >T (p.Gln564*) located 15-nucleotides downstream of the probe ligation point, while in two of the samples with the reduced signal in exon 10 we found the missense mutation c.1972C >T (p.Arg658Cys) located 10-nucleotides downstream of the probe ligation point. In the three remaining samples with the reduced signal in exon 10 we found the silent mutation c.1977A >G (p.Arg659Arg) located 5-nucleotides downstream of the probe ligation point. Two of the mutations, c.1690C >T and c.1977A >G, were previously reported as definitely pathological. Although c.1977A >G is an apparently silent mutation, it affects several exonic splicing enhancer (ESE) motifs, resulting in the deletion of exons 2–9 and leading to a frameshift and the premature termination of translation (p.Cys53_Trp635delinsfs*12)^23^. The third mutation, c.1972C >T, was reported either as a potentially pathological or as an unclassified variant (Table 1)^7^,^10^,^12^,^19^. It was also shown that this rare sequence variant (<1%) is a risk allele associated with lung cancer (OR = 1.55)^38^. Further computational analysis of the potential functional consequences of this mutation showed that it causes substitution of a very-conserved arginine in position 658 (e.g., PANTHER, http://www.pantherdb.org/) and has a highly deleterious and destabilizing effect on protein structure (e.g., PolyPhen2, http://genetics.bwh.harvard.edu/pph2/) (Table 1). The high resolution melting (HRM) screening of c.1977A >G and c.1972C >T in a panel of 1000 unselected control samples led to the identification of one and three cases with these mutations, respectively (Ratajska M *et al.* unpublished).

Six of the identified point mutations were detected in patients with familial breast cancer, and one mutation, c.1690C >T, was detected in patient #53, who was originally enrolled as a patient with unselected ovarian cancer (Table 1). However, further analysis of the family of patient #53 revealed an aggregation of the disease within the family (Supplementary Materials: Supplementary Fig. S1 online).

Although all three of the detected single nucleotide substitutions are either deleterious or potentially deleterious mutations, the detailed explanation of their role in the predisposition of breast and/or ovarian cancer requires further functional and epidemiological analysis, which was not the subject of this study. Nonetheless, the identification of these single nucleotide variants with the use of an assay that is dedicated to the detection of large heterozygous mutations makes it highly unlikely that the lack detection of large mutations was due to false-negative errors.

An additional result of our study is the production of a homemade MLPA assay that can be used in any further analyses of large mutations (both germline and somatic) in *BARD1* in both breast/ovarian cancer as well as in other types of cancer. Our analysis was conducted on large number of samples, which further helped to prove the robustness and high reliability of this test.

## Conclusions

In summary, our study, conducted on a group of 817 patients, did not lead to the detection of any large mutations in *BARD1.* Although we cannot exclude the presence of such mutations in *BARD1*, our results clearly indicate that these mutations do not contribute substantially (>>10% of the total *BARD1* mutations) to *BARD1* sequence variation and, subsequently, to familial breast and/or ovarian cancer aggregation. However, it does not deny the role of *BARD1* as the breast cancer susceptibility gene. It has to be also noted that our results may be specific to the Polish population.

## Methods

The study comprises DNA samples (extracted from whole blood) from 504-non-BRCA patients (tested for the 5 most common *BRCA1* mutations in the Polish population, c.68_69delAG, c.181T >G, c.3700_3704del5, c.4034delA, c.5266dupC, cumulatively accounting for >90% of all *BRCA* mutations^39^) from families with breast and/or ovarian cancer aggregation (as previously defined^40^) and 313 patients with ovarian cancer that was unselected in terms of the familial history of the disease. The patients’ blood samples were collected between 1999 and 2012. Informed consent was obtained from all of the patients, and the study was approved by the medical review board of Medical University of Gdansk (NKEBN/399/2011-2012). The methods were carried out in accordance with the approved guidelines.

The multiplex ligation-dependent probe amplification (MLPA) probes and the probe-set layout were designed according to a previously proposed and well validated strategy^37^,^41^. This strategy exclusively utilizes short oligonucleotide probes that can easily be generated via standard chemical synthesis. The sequences and detailed characteristics of all of the probes as well as their exact position in the *BARD1* sequence are shown in Supplementary Materials: Supplementary Table S1 and Supplementary Data online, respectively. The MLPA analysis was performed with the use of a homemade *BARD1* assay (combined with reagents purchased from MRC-Holland, Amsterdam, The Netherlands), according to general recommendations published in previous studies^37^,^42^. The products of the MLPA reactions were diluted 20× in HiDi formamide containing GS Liz600, which was used as a DNA sizing standard, and separated by size with capillary electrophoresis (POP7 polymer; ABI Prism 3130XL apparatus; Applied Biosystems, Carlsbad, CA, USA). The obtained electropherograms were analyzed using GeneMarker software (version 2.2.0; SoftGenetics, State College, PA, USA). The normalized signal of each probe (peak height divided by the average peak height of the control probes) was divided by the corresponding signal of a reference probe and multiplied by 2. The obtained values that correspond to the copy number of particular exons/regions were visualized in bar graphs. The analysis of samples with detected aberrant MLPA pattern (with mutations) was repeated at least two times.

The *Hind*III digested positive control sample was generated as follows; 1 μg of genomic DNA was incubated overnight with 20 U of *Hind*III in conditions recommended by manufacturer (Thermo Fisher Scientific, Lafayette, CO, USA) and then mixed with equal amount of undigested DNA. To generate artificial control sample with masked target sequences of the BARD1_e06 and BARD1_e07 probes, 7 fmol of each of two masking-oligonucleotides: GAA TTA TTG CTC CAG CAT AAG GCA TTG GTG AA (specific to BARD1_e06) and GAA AGT ATG AAA TCG CTA TTG CTG CTA CCA GAG (specific to BARD1_e07) were added to the MLPA reaction together with the MLPA probe mix at the hybridization step.

The mutation sequencing was performed on ABI Prism 3130 genetic analyzer; Applied Biosystems, Carlsbad, CA, USA, according to the manufacturer’s general recommendations.

## Author Contributions

K.K. – designed MLPA assay, performed MLPA validation, analyzed MLPA test, point mutations analysis, prepared artificial positive control samples, participated in manuscript preparation; M.R. – participated in conceiving the study and manuscript preparation, coordinated samples selection and participated in DNA extraction and samples characterization; K.C. – participated in MLPA analysis; A.K. – participated in DNA extraction and samples characterization; I.B. – provided and characterized familial breast and ovarian cancer samples; M.K. – participated in samples selection and DNA isolation; M.S., J.D., D.W., M.S. – provided unselected ovarian cancer samples; M.B. – participated in mutation characterization; A.Z. – participated in MLPA analysis; B.N. – provided positive control sample; I.I.F.- participated in data interpretation and manuscript preparation; J.L. – supervised clinical samples characterization and selection, participated in conceiving the study and manuscript preparation; P.K. – supervised MLPA analysis and interpretation, participated in conceiving the study and manuscript preparation, coordinated the study. All authors read and approved the final draft.

## Additional Information

**How to cite this article**: Klonowska, K. *et al.* Analysis of large mutations in *BARD1* in patients with breast and/or ovarian cancer: the Polish population as an example. *Sci. Rep.* **5**, 10424; doi: 10.1038/srep10424 (2015).

## Supplementary Material

Supporting Information

## Figures and Tables

**Table 1:** The point mutations detected in this study in breast and/or ovarian cancer susceptible patients.

**sample ID**	**sample type**	**type of family**	**nucleotide change**	**canonical AA translation of nt change**	**predicted effect of the mutation**
#53	unselected ovarian	Br/Ov	c.1690C >T	p.Gln564*	**deleterious nonsense mutation**^23^,^27^
#4031	familial	Br	c.1690C >T	p.Gln564*	
#4163	familial	Br/Ov	c.1972C >T	p.Arg658Cys	**missense mutation**, described either as deleterious, potentially deleterious or neutral^7^,^10^,^12^,^19^,^38^PANTHER: change in conserved AA, score -3.030/-10; PolyPhen2: probably damaging, score 0.995/1
#4349	familial	Br	c.1972C >T	p.Arg658Cys	
#4062	familial	Br/Ov	c.1977A >G	p.Arg659Arg	**deleterious splice mutation** (exons 2-9 deletion; p.Cys53_Trp635delinsfs*12)^23^
#4217	familial	Br/Ov	c.1977A >G	p.Arg659Arg	
#4321	familial	Br	c.1977A >G	p.Arg659Arg	

Br – site specific breast cancer family, Br/Ov – breast and ovarian cancer family; The variation sites are defined based on NM_000465 *BARD1* sequence.
