# Supplementary material for: Analysis of large mutations in BARD1 in patients with breast and/or ovarian cancer: the Polish population as an example
Source: Sci Rep. 2015 May 21;5:10424. doi: 10.1038/srep10424 (PMC4439969; doi:10.1038/srep10424)
Supplement: Supporting Information [file srep10424-s1.pdf]

Supplementary Materials for:

Analysis of large mutations in *BARD1* in patients with breast and/or ovarian cancer: the Polish population as an example

Katarzyna Klonowska[a], Magdalena Ratajska[c], Karol Czubak[a], Alina Kuzniacka[c], Izabela Brozek[c], Magdalena Koczkowska[c], Marcin Sniadecki[c], Jaroslaw Debniak[c], Dariusz Wydra[c], Magdalena Balut[c], Maciej Stukan[d], Agnieszka Zmienko[a,b], Beata Nowakowska[e], Irmgard Irminger-Finger[f], Janusz Limon[c] and Piotr Kozlowski[a,b]*

[a]European Centre for Bioinformatics and Genomics, Institute of Bioorganic Chemistry, Polish Academy of Sciences, Noskowskiego 12/14, 61-704 Poznan, Poland
[b]Poznan University of Technology, Pl. Marii Sklodowskiej-Curie 5, 60-965 Poznan, Poland
[c]Medical University of Gdansk, Marii Sklodowskiej-Curie 3a, 80-211 Gdansk, Poland
[d]Gdynia Oncology Centre, Powstania Styczniowego 1, 81-519 Gdynia, Poland
[e]Department of Medical Genetics, Institute of Mother and Child, Kasprzaka 17A, 01-211 Warsaw, Poland
[f]University Hospitals of Geneva, HUG, Geneva, Switzerland

*corresponding author: Piotr Kozlowski - kozlowp@yahoo.com
tel: +48 616653100, fax: +48 618520532, IChB PAS, Noskowskiego 12/14, 61-704 Poznan, Poland

Supplementary data

The *BARD1* sequence with the positions of the MLPA probes marked.

The NM_000465 *BARD1* sequence (Mar 2006 NCBI36/hg18 assembly) with the locations of the MLPA probes marked. The 5' half-probes are marked in yellow, and the 3' half-probes are marked in green. The ID of each probe is shown next to the *BARD1* sequence. The exons are indicated in blue, the low complexity/repetitive regions are labeled with lower case letters and the positions of the SNPs are indicated in red.

```
BARD1
>chr2:215296520-215387673 (reverse complement)

GAAGTGGCCTTTTCCAGTGCATAATACCATAATTAAAAATCAGAGAAGAA
TTAATATAAAGGTAAGAATTTGTGAGATATCTTTACACTCCTTGGGAGTA
GAGTCTGGGTATTATGGATATCCCATTTTGCTTGTTATATATGTTTATCC
AAGTGAATATGAGGAAGATTTACCTCTTTGTAAAACACTTCATAACACAA
CAGAAGAGAAAACAGCATTTTGACTGTTTTCAACAAACCATTATATAAGC
CTGAAGATCAGAAAAGAATTCAAGGCTGGAGATGCCTTCATAGCATGGTA
ACACTACAGAGGGTAACTGAAGCAGTCGGGGGGATGAGGGTTTAAGTGGG
CCTGGAGAGAGGAGGATAGGTAAATAAGGCTGAAGCAAGCTTATCTGCAT
GCCTCAGAATCCACCCAGGACTACAGTGTTACAGTGGGGGAGGGATGGAT
GATTTTGAGTCTAGGATACTTGGGAATACTGCTTACTAGGAAGAGAACAG
ACTAGGAGTGAAATGGAGCAACAAGTCTAAACTTTTATGTACACAGGAGG
TCCTTGCAGGGAAAACTCTGAAAAAGAAATGATACCATTTAGCCCTTTTT
AAAGTGAGAAGAAGAGGACATTGTTCATGTCCTAAACCATGGCTTTACTG
AGACCCTGCTGATGGCTGGAGTTCCCTGCAGAACTTTTTCTCTGGGACAA
GAATTCCAGGATTTTACACATTGTTGGATGTACCCCATGGGAGAAGGAAT
GTGCCCTTGAAACTCAGGATCCAGGGTTAAGAGGAAGAGTTGGTTCAAAA
CAAACATCCCATCTCCACTAAAGGCAAAGCACCAGCAAGCTAAGCTGTAG
AAAAGCAAGGCTGGAAGGAAGAAAATGGAATGGGAAGTTGCACATCGGAG
AGTAACCCTGCTTATTCTCTTTATGAGTTTCATTATGGCCACTATTAAAC
AGCTGCCACGAATGTCATGACCCCTGGGGAGATGGAACAGGACAATGGCA
GAAGAAAGGAAAAAAGCAAACAAACAGTAGAAAGAACTAATTGTATCTGC
ATCATAATTCATTTAATAGCATGGTATGAATACGCCAGTTATAAATGTTT
GGTCCCAAATCATTTTACCAAAATGTGAAATTTTCATTTACTCTGATCAC
AGTTAAATTGTGCCccaagtatagtggcgcacgcctgtagccccagctac
tcggcagggtgaggcacgagaatcgcttgaaccccttgaacgcaggaggc
agaggccacactgagctgagattgtgccactgcactccagcctgggtgac
agagcaagactctgtctcCCCCGCCCCCCCGCAAAAAAAAAACTGTGCCC
TTAATAAAAAAAAAATTGATAAAAATGTTCAGTGTTATTCTTTAGTAAATA
AAGCATTAACAGTACTGATTATATATAATCATCCTCAATCagttaccatt
tactgagtacttatggtgtatgtgattttacatttaatcttatttaatag
tcctccaaatctacccgataaatattatccccattttgcagacaaggaaa
ctaatgctcagagagcttaaggcttggtttaaatcacacaattatttagt
gaaagtgggattataagtaggacaatctatctcctgaaatcctcaattta
ccccattaacaccatagtatctctcCAGGTTTTAACGTTTTAAGTTTTCG
TTCTTTTTCTTTTTCTTTCTTTTCCttttgtgtttttttttttttttttt
tttttgagacagtctcgctccgttgcccaggctggagggcagcgacacca
tcttgcctcactgcaaccttcacctcctggcttccagctatttttctgcc
tcagcctcctgaggaactgggattacaggtgcatgccaccacacctggct
aatttttatatttttagtagagacggggtttcaccatgttagccaggctg
gccttgaactcctgacctcaagtgatccacctgccttggcctcccaaagt
gctgggattacaagtgtgagccaccgtgcctgaccCATTTTAAGTTTTCT
AGAACTTGATTTCTCCCACGCTCAACAAAGAGTAAGCACTGGCTTAACAG
GACTAATAAGGACTAACTTCTTAGGTGCCTCTGAGTTAAAAAAAAAAATT
GGTTCGTTTGTGTGCTATCATAGTTTCAGTTAAAACATTTAATAAAGCCT
TATTTAGTTGTGCTTATTCTAactaaccaagtgtgtgattttggccaagt
catttaaactccttgagtttTCATTATTTAGTGGAGCTACAAggcacagt
ggctcacacctgtaatcccagcattttgggaggctgaggcagaagaattg
cttgaggtcaggagttctagagcagcctggccaacatagagagaccctgt
ctctacaaataattaaaaaaaaaattatccaggcatggtggggtgcacct
gcacaggtcccagctactcgggaggctgaggtgggagggtcacttgagcc
```

caggagttcaaagctgcagtgagctgagatcgtaccactgtactccatcc
tgggcaacagagtgagaccttgtcccaaaaaaCCCCTCAAAAGACCAAAA
ATATAATTTACTGgggccattaataatcaaaaagatctgggttaatattc
tgccactgttgttatctgtgcctccatcctggacaagtATGCttttcttt
ttacaaatcttactttctctaaagatgggggtcttgctatgttgcctag
gctggccttgaaccccggggctgaagtgattctcctgcctcagcctccca
agtagctaggactacaggcatgtgcTGAGCCccttgggcaagtaaattaa
gatctctgagccacacctgcctcctctTATTAAAGAGCActgtcttagct
gttataacaaaatactataatctagatggcttaagcaacagaaatttgtc
atatttctggagtctagaagtctgagattagcgtgccagtatgactgggt
tttggggagggccctcttcttttgcgttcttgctgtgtcctcatgtggtg
gagagagagagaactctctcttcttacaaggtcattaaccccatcatggg
ggctctgccctcataaccccatctaaatctaactaccacccaaaggtccc
ctccaaatactatcacgttggggttacaacttttaatatatgaatttggg
ggtgacacTACAGatactaatgcccatttcatagggtccctataaggctt
aaggcaggtattaacataggaaagcacttaaagctgggtctggcTTGGGT
AGGTAGttcaatgattcaacaaacactgagcacctacctggagccaagca
ctgcatgtgccacatgaagcgatattgggaaatgagtcacatgcagccaa
tctctggccttttggaggttttgaactagaaggggacacgcacataatcg
tatgtgtgtgtatctacatacacaggtgatatgatgcacctgagagaatc
cagtctaggaactaggaaaacctttaaggagtgatacttcagctgtattc
tgaaggatgaggaatggagaggaggccaattccaggttccaaagtgaatc
tttgcgcaaaagccatgaggcagcaaggtgcaggggcttttaatgaccta
agggaacgcactgtggggttgggaccatgatggccagaggaggttttgac
aagaggctagtcaaagagCAGAGAAAAACTTTAAGGAGTTTTAAGAAAGG
GAAGTGCCACGATGAGTTTTGTGTTTGGAAATGTTTTAGGCGGCCACACC
GCAGTCTCGGGACTGGCTGGGACCTGGATAGACACTTGGATATCAGCTAG BARD1_f5'
AAAGCTACGACAGGAAACCAGGCGAGGACGAGGCAACTGGGGATGTTGTG
GAGCAGAGACGGAGAGAAAATGGGTGCATTCGAGACAAGTTAGGGGGAAA
AAATGCAAGGACTTGGAAATGAACTTGGGGCGCGGCAGGAAGGCATGACG
GGTTGCTCTGTAGGTCTTATCTGTAAATTACGGCGATCAGTGAAAGATCT
GGAGGAGGAAGGTGGACACACTCTCTAACAAAAAAAACCCTTTTTGAAAT
TTTATACCAATATTTTAAAAGTAAACCAGATCTTTTCAGACATGCCTTTG
AGCTGATATTTGTTAACTAGTTAGAATTAGAAACTTTCCTTATTTTTACT
CAGTTACAATATACGCCACAGCTGAGGTGAGAGGAAAGAAAAGGTTGCTT
TCTTAGGAACAAAGAGTGGTACCTTCAGTATCGTGGGCAAAGCTTTTCCA
AGTCCAACAGCAGTCAAAACAGCGCTTTTTATAAATAACACTCAGCTAAA
AGTTTCTGGGTTTGTGATTGTTCCAACGGTTAAGCTCGGATGAGGGTCCC
TGGAGTCGTAGCTCCCGGGAAACGTCGACTGGCTTTCCACCTGGACTTCA
TCCGTCCAGGCAGCCCAGAGGGGCTTCAGGCCCCGCCCGCTCTCCTGCCA
ACTACAGCCTCGCGACTGCGCTCAGCCTTCAGGCCCCGCCCCTTCGGTCA
AGCGGCGTGCTCTCACTGCACGGCGCCTGGGCCCCGCGCGCCGGGACCTC
GGTTTCAGCCGTCCTGTCCTGCCCCGAGGCCCCTAGGCCCCGCCCCTGGG
CCCCGCGCGCCAGGACTTCGGTTTCGACCGTCCTGTCCCGCCCCGAGGCT
CCTAGGCCCCGCCCCCTCTGTCCCCGGCGTGTTCTCGCGGCTCCGCCCCT
AGGACCCGCGCGCCGGGACTTTGGCAAGTTTCAGCCGTCCGGCCCCGCCC
CCTCGGTCCCACGGCTCTCGCGGCCCCTCCCCTAAGTCCCACACGCCGGG
ACTTTGGCAAGTTTCAGCCTCCAGCCCCACCCCTAGGTCCCGCCCACTCG
GCCAGCGGCTGGCTCTCGCGGCCCCGCCCCTGTGCCCTGCGAGTCCCTAT
TTTGGGAGCATTGCGGccgccgtgccccgcccctccccgcgcgccccgcc
ccTCTGGCGGCCCGCCGTCCCAGACGCGGGAAGAGCTTGGCCGGTTTCGA exon 1
GTCGCTGGCCTGCAGCTTCCCTGTGGTTTCCCGAGGCTTCCTTGCTTCCC
GCTCTGCGAGGAGCCTTTCATCCGAAGGCGGGACGATGCCGGATAATCGG
CAGCCGAGGAACCGGCAGCCGAGGATCCGCTCCGGGAACGAGCCTCGTTC
CGCGCCCGCCATGGAACCGGATGGTCGCGGTGCCTGGGCCCACAGTCGCG
CCGCGCTCGACCGCCTGGAGAAGCTGCTGCGCTGCTCGCGTTGGTAAAGA
CGGAGCTTCTTGGGGGTGGCTGCGAGGGCACGGGTCGCACAGTTTCTGGG BARD1_e01''
GGCGGCAGAATCTTTTCAAATCTTCCGTTTCCTCCTTCCGTTCCCGCGCT
GCAGTCGGGTCGGCGTGCGGTTAGCACCTGCCGGGGGATATAGTATTAAC
AACTTCTGCTTCTCATTCACTTTATTTTTGGGCGACTTACCGGCCTCCCC
TTGCCCTGAATCCAACTGAAAcggtagtttttgaacttcagcgggctgaa
gaaccgtctggaggtgtggctaaaaaaatgttcatcccggtcgcgcctcc
agagtttgaatcggctggggtggggctgaggcttctgcatttttttacCC
GGCCCTGGATTACCCCGCTGCTTTCCGGGAGCTGTGGCGAATTGGGCTGG

CGGGCCGCCCCGGAGACCCTCTAAATTAGAAGCAGCTGCCACTCTAAGTT
AAACTGGCCTTTTTGACATTTTCTCCGTGCCAGCTTTTTCGAGTGAGATG
GGATGGAGCATCGGATATCTACCATAGTTGTAGATTGAAGATGGCACGGA
ATTTCTCATTTTCTTAGTTTGCTCAAAAGACTGTATGTCTGGTGTCCCCG
CTCTTAGTGATGCTGTTTATTGTTTTCCTTCATGCTGTCACATTATGGGA
GTCCTCTCAGGGTTTTCATTCCGTCCAAAGACATTTTCATGAGTGTTACT
TCTTGCTTTTTTTCGTGTAGCTCTTTCTAGTATACAAACCAAGAAGGTTT
GTAAAACCCAAGAAAGGTTTGTCTCATTTTCATTTCTTCTGAAAAGCATT
TTTATTGAGCACTTGCTGTGTGTACAGCTAAGTATTTCTGTATGAAGATC
ACTGGCAGTTTTTTTGTTATTTGTAGCTGTCAAAAGTTTATATCCTTTTT
GGACTTTTTTCCCCTTTGATTTCTAACTTGCAGAACTTAAGTTACCTAGT
TGCATAAAGTATAAGTAACACAACtttttctttttctttcctttttttgaga
cggagtctcgctctgtcgcccaggctggagtgcagtggcacgatcttgtc
tcactgcaagctccgcctcccgggttcacgccattctcctgcttcagcct
cccaagtagctgggactgcaggcgcccgccatcatgcccggctaattttt
tgtatttttagtacagatggggtttcaccgtgttagccaggatggtctcc
gtctcctgacctcgtgatccgcccgcctctgcctcccaaagtgctgggat
tacaggcgtgagccacggtgcccggccAAACTTTTTCTTTAAAAATGAAA
AGGACTTAACTGTCAGAGTTTTGCACTTATATTTTCTTTGACGAAAACTT
TTAATATCTGTGACAAAATGACATTGCACTTCTTAGAAAAGTGACACTCA
GATAAGTGAGTACATGAAGACATTTGAGTTGTGTGTTTGTTTTCCTGTAG
CACATGGGTCATCTGAGAATAGGAAATTTAAAACTGGCACTGCTAGTTGC
AATAACAGAAGCATTATAGTTCACACCTTGATAGTCCAACGAAAGTTAGA
CATTCCCTTGGCACATACCTATgtggaagcatcgggcttcacttagactg
tggtcagaatgcctgagttcaggttctagctttgacactattgagtaggc
caagttattcatctctcattttccggatatgagtaatactaatgattagc
ttattttcttgtgaggattcattaataatcataaatttattgagcaccca
taaagtgccagaccctattctgggtattgggatttgttagtgaacaaag
cagacaataaattccatctcttagggagcttttatggaagaaacaaAATG
AAATTTTACATGTATGTAAAGTATTGAGTTTGGTGGTTGTGATAGAATAA
ATGATAAATAATGTGTGTCTTAATCTAAAAGAATGTGGTGTTTGCTTGCT
ACATGATTTGGGAGAGACAAAATAGATAATATATATGAATATATGTTGTC
CTGAAAAATAACAAAGCCGGTTTCACTTTTTATCCCCTACCAAATATTTT
TCCAGTTTCTAACTGAAGTGAGTAGACAGTAAGAGGCAACGGAGTGTAAA
CAGACATCAGTAACCAGGCCCAGACTTTTAGCATGGCTCACCCATTTGAC
TACAGAATGCTATTGAATGCTATTGATTTTTTTTTTTTTAATCCGAACCT
CTATAGATGAACTGAATCTTAACTCTTAGACTGCATGTTTTCATGTaggg
gtctgcaaatttttttcggtaaagggccaggtaattaatattttaggcctt
tctgcccgcaaatagtttttgttcttatttatttttacatctcttaaaaa
tgtaaaaaccattctttccttaagggctgtacagaaacagactgctggct
tggtttggcccacaggcaatagttGCTAAACTGTCGATAAAGTCTTGATA
AACTTAATGTCTCAAGTTTAAAAATCCTTTTTGGTATACAATTTTATATC
ACATGGGGTGCCtttttttttttttttcccctaggcaaagtttcactctg
tcacccaagctggagtgcagtggcatgatttcagctcactgcaacctcct
tcccccgggttcaagcaattctcttgcctcagcctcctgagtagctggaa
ctacaggtgcatgccaccatgcccggctaattttgtatttttactagag
acggggtttcatcatgttggccaggctggtctccaactcctgacctcaag
tgatccgcccacttcagcctcccaaagtgctgggattacaggcgtgagcc
accatgcctggtcTTTACATGGGCTTTAATGTTTAGAAATGATTTTGCTT
TCCTCTGTAGGTGTCTCTGCAACTTTGGCACTATCTATAGTGAAAAAAGC
ATGGCCAGCTGCACCTTACTCAAGGAGGAAAAGCATTGTTCCTACCTCTG
CCTTTGCCCTGGTAGCCTCCTGACATTGCCGGCAAGGAGCAACTCAGAAT
GGTCTGCTTCTTCCATTTGCTACCCTCCATTCAAAGCAGTGTCATATTAT
TTGAAATTGTGTCAGTATGTCCTTTTACAGAGTTTCAGAGTTCTACCCAG
TGGGCTAGTGATTACCCCGATTACCCTCACATTCAGCTTGCAGCTGACAT
CTGCCTTGTGTTGCACATGGATCTGTTTTGCAGCAAGCATTACTTGCTGG
TGAGGGTGGATTGGTACATAACCGGGCATCCTTTCTTGACCTTTAACATG
AATCTGGCTTCAAGGTGAAGCCGATGAAAGTGTTCAAGAGGCCAGATGTG
ACATTTATATTAGGACCAAGCCTCACAACCAGAAAGAAGCTATTTAAGGA
TTTGGAAAAATGAGACAGGAAGACCATATTATTTGAGAGGCTGGCAGAAA
GCACAGTAATAATGTGTTCCTAAAGGAAGCAGGAAATGGTAATAGGAGCT
GCAGTGTAGAAGCCCCAGGTGTCATATAACACTGTTTTGTGAATTAATAG
GTACCTCTTGGCATATTTTTGAGGGTGAGAGTGGGATTAGACATCTATGA
CAATTGTAACGTGGGCCTTTTTAATCAACTTTTTATGAAAAGTTAGAAAG

TAATCTTTTAACCTTTATGATAGGTATTTTTTTTTTTTCTGAGTTCTTGTT
TAGGAGATGATCAAGCTTAATTTTGTATTCTCTCATACTTTGAGGcagca
tagtgtagatgttaaatgtgtggaatctggagttagattccctgggttca
aatcctggctccattatttaggttattttatctttttatgcttcctttc
ttcatctgcacaaaatgagtggatattcataAGATGCCTTGAGCTGTGTC
TGTTATGTACTAAATACTATATGAATATTTGTGAAATAAAGGATAGGTTC
TCTGACTCCGGGTTCAGCTTTTGCCTTTCTTCATGAGTTTTGAATGTTCA
GAGGCATTCTCACAGAGCAGTGATCCCCAAATGGAATGATTTCTCCCTGG
CACTGGTTATTCATTAGAATAACGAGAAATCACTGAGACACTGTGTAAGG
GGTGATAAGGTGATGCATTTTCTACTTCTGCCTTTAAGGAGTGTATGCTT
AATTATAAGGGATAAAATAAATACAAGTTCAGGGTAAATTGTCGTGCAGG
TCGACATAGTGCTGAGAAGAGTACTTGGGTAGGGCCAGTGGAAGGAAGCT
TCATGGGCTTTTAAATTGAAATATGAATAGAtttttctttttttctgagacg
gagtctccctctgtcacccaggctggagtgcggtggtgtgatctcggctc
actgcaacctccgcctgccaggttcaagtgattctcctgcctcagcctcc
tgagtagctgggattacaggtgagcgccaccaaacccggctaattttttgt
atttttagtagacacggggtttcaccatgttgctcaggctggtctcgaac
tcctgaccttgtgatccgcacttcttggcctcctaaagtgctaggattac
aggcgtgagccaccacaccaggccGAGATGCGAATAGATTTTTAAAATGG
AGCCAGGTTGCAGGGAGCTGGGGTGATGGGTGTTATTTGCAAATAACTAG
CTATAAAGAATTGTAGGGTAAAGGGGGACTGGGTCAGGTGAACAGAGAGT
TAAGACAGGGTCAAATCAGTGATAGTAGCCTAACAACAGAATACTCAGTT
GGTTTATATAGTTAACTTCCTAAAGAgagcaatttgtatagctttcctgg
actctggttcctcatctataaaatgaggaaaatggactcgttgatttgta
aagttctctccagtttgcagatttcatagttATTTGTGTCCACTGGAtga
agtttaacatacatacagaaaaggcacaaatcattggtgttcagcttgTA
TCGTATCTTCCAAGTTAGAATTTATTTGTGAGATTTATTTATTTATGGAA
TATTTTGAGTGATGTCTGTCTCCTTCAGTTATATATTTAGTAAAACTAGG
TTCACGTTTGTAGCCACAATGCCAGACACATAGTTTCTTAGTGTATGTTA
AATTGATGTATGAAAGAACTTAGGTTAAGGTAGGATCACATTTGAAGGGC
CTTTGCAAGTGGTAATACCAGAAGATCTAGTTAATTTACATGAATTGCTA
AGAATCCCCTATAGGTTCCATGTCATTATCCATATACTTTACTTGTTAAA
CACTTTATTCTTCTAAAGATAAACACAAGAAAAATTGTTTAATTGGCTTG
GTGAGCCTGCACATTTAGATGTTCAACATGTTGGTCCACACGTTAAAATT
ACCTTGAGGATTCTTTAAAATGTAAGAGCACTTTGGGAGCTGTCTTGCCT
AATATTAAATAGCAGTTGATATTTCATGGAGATACAGGGGAGCCATTCTA
TTAAGAAATAGATCTGCCTTGGTATTATCATCTCAGATGGATTTACCTTC
CTTTTTTTCTTTTCGAAGAAAATTTTGTATCAGTAAGAACTTTAAAAAAA
ATTGTTCTCACTACTTAATTCTTCCCTAAGTTATCCTCTGACAGCACTGA
TCtgaagggggtgggttgcccctccacacctgtgggtgtttctcgtaaggt
ggaacgagagacttggaaaagaaaaagacacagatacaaagtatagagaa
agaaataaggggacccagggaaccagcgttcagcatatggaggatcccgc
cagcctctgagttcccttagtatttattgatcattcatgggtgtttctcc
gagaggggatgtgtcagggtcacaagacaatagtggggagagggtcagc
agacaaacacgtgaacaaaggtctttgcaacatagacaaggtaaaggatt
aagtactgtgcttttagatatgcatacacagaaacatctcaatgctttac
aaagcagtattgctgcccgcacgtcccacctccagccctaaggcggtttt
tccctatctcagtagatggaacctacaatcgggttttatactgagacatt
ccattgcccagggacgggcaggagacagatgccttcctcttgtctcaact
gcaagaggcttgccttcctcttatactaatcctcctcagcacagaccctt
tacgggtgtcgggctgggggacggtcaggtctttcccttccccacaaggcc
atatttcagactatcacatggggagaaaccttggacaatacctggctttc
ctaggcaggggtccttccgcagtgtttgtgtccctgggtacttgagatta
gggagtggtgatgactcttaaggagcaagctgccttcaagcatctgttta
acaaagcacatcttgcacaacccttaatccatttaaccctgagtttgaca
cagcacatgtttcagagagcaccgggttgggggtagggtcatagattaac
agaatctcaaggcagaagaatttttcttagtacagaataaaatggagtct
cctatgtctacttctttctatacagacacagtaacaatctgatctctctt
gcttttccccacaCTGATCTTTCTAAATCAACTACATGTAAAGTTACAGA
AGTATGACTGTAGATGGGGAAAAAGTAGAGTAGCTGAATTTTCAAGGTAC
AGAAATCCTGTTACCATGCAAATGTCTTCTCTTATTTGTTTGGATTTTTT
CTATCTAATATATATGTATATCAAACAGAAAATAGGGTAAGGATAAAATG
GAATTGTTAATACAAATGCATTTTTGTATATAGGCTTCCATTTGGTTCAT
TCACTAAGAACTTATTGAAATCCTGCAGTTAATCTAGCACCATGCTAGGT

AGGTAATGGTGGGTTGGGGAGCTCAGAAAAAATGAAAGTTAAGATCTCTT
GTCTTAGGGCTGGAGTCTAATTTCAAGACAAGAGAAATAGAAAATCAGGA
AGTTAAAATAcagatgctcctgaacttgagatggggttatgtctggatca
acccatagtaagtttaaaatattgtatgtcaaaaatatatttaatataca
taccctaccaaacatcatagcatagcccagcctaccttaaacatgcccag
aacactaacattagcccagttgggttaaatcatctaacacaaagcctatt
ttataataaagtgtttaatagctcatgtaatttattgaataccatactaa
aagtgaaaaacagaatgacttcctgctgtggcccagcattgtactgcata
ttgctagcctgggaaaagatcaaaattcaaagtgtgatttctattgaatg
tgtattacatttgcaggataataaagctaaaaaattgtgagtcgaaccat
tgtgagtcagggatcatctgTACAAAATATTTAGTTTTCAagcctgggca
acatggcaaaaccccacctctacaaaaaattagccaggtatggtggtgtg
cacctgtagtctcagctatgtgggaggctgagatgggaggatttcttcag
ctcaggagttcaaagctcagtgagctgtgatcatgccactgtactccagc
ctgggcgacagagcaagatcttgttttaaaaaaaacaaaaaCAACCCCCC
CCCCCGCCAAATATTTGGTTTTCAACATTGAATCCCAGAACCCCAACTAG
ATGCTCTCTTCTTTGTAACTTTAGAGCATTTTGTACATCTTTTAAGTCAT
ATTAATTTGGTTTATGTAATTAATTGGCCAGATTATAAGACCCTTAGAAA
AGAGTGGAACACAGTCAACTTTGTTCTTTAGATGTTACATGGCATGAATT
TTGCAGAAGTGATGGCTGGAGGCTGAAATCCAAGAGATGGACATAGGCTA
ATAATAGTTGTAAGAGACACTGGCCTGACCACAGCAGAGTTTTTGTTGAG
GAGCAATGAGAGAAAAGGTTGCATGGCTTAGGTGTTACAGATTGTGAAGA
ACTTTTTAATAGTAAATTTTTTTTTAATGGTAAACAGTGTGTGAAAAGAA
TTAAAATAACACAGTATATGGCCATTCAGTATTTGTTAAATGAACGACAG
ATAAGTGTGAATCTTCTTCAGTAACTTATAATTATCAGTTTCTTGTGTAT
CCTTCAAGAGGTTCTATGTTCATATAAGCATTGTACTGCCTCAGATAAGC
AGATACTAAACACCATGTAGTTGTTGcatgattcatttaaccatttcctt
attggaaacatttaggttgtttgtggtcttttgctgtaacaagcagtgct
ggagtgaTACTGATGTCATGTGCCTTTACATTTAAAATGTttttaaataa
agttatacatgaacgtagttgacagagtcaataggacagactattaatga
aaacattctttgcttcatccttcatcacccctgagttctatgcatcagaa
acatcttttgtcttttttttagttttacaagcatgtacctctacatttcc
aaatatgtttatattgctatttatttttttttccattttagatgttatta
acttcctaactattctcaaagatgaATTTTCTTACTACTGTTAACTTTGA
CAGTGGAAGTTAGtggatgtagaattcaaagtacaaaataattgtcaaga
attattttgaaggcctcgttccacagttctgtagcatctcttattaccct
tgagaagtcttatgctatcctgattcctttttttaaaaaaagtatgacat
ttaattttattatttcttaaatctctggaaggttttagaatcttgtctag
atatctttgatgtttgaaagtttcaTTTttgcaagtatctagtgttgctg
aaggacaaattcctggcagtggaatagctgagttaaagttggatgcattt
atattcttgataggtatctcctagttgTAATAATTTTTTAAAAAAGtttt
tctaaagatacatgttcttgttgtgttgtccaggctggagcgcagtggct
attcacaggcacgatcatggcacactatagccctgaactcttgggctcaa
gctttcctcctgcctcagcctcctgagtagctgggattgcaggcatgtgc
cactgAATTTGGCACTCCCTTCTCTCTCAGCTTGGCTCACACACTTCCTT
CTTGTTCCCTCCCCCAATACACACAAGTGCACATACTTCTCATTCACATT
TCCATTATCTTTCCTTATCCGTTAACAGCAACACAAATATATATTCTAAC
TCACTAATTAGAATAATTAGTACAACTGATCAATTATTCCCACATGTTAA
ATCATTACTAATATGTGATAGCACCTTATTGCTAGAGTGAAGGTTAAGAA
CTATAGCTAAAGAGTTCAGTCATCTTAGGAAGGAAatttaaagttgagaa
acagaatcaaggcgttgagtcaagtattgccagagaaagccggaaggcta
cggactgcatcctgggggtatgcctacatggagaggtaggaaaagcagct
aaggaaatgctgaaataggcagatcaatgtgtataaaaatattaaaatgc
ggtggaaggaaaggtttcaggaaagaagatgaggtcagtgctgtttccca
gagtggtcagagagaaggggaccaaaaatgctttggagaacagtttcagc
agagtggagggagttaaagctaggaaggtgaggagatgggagtagctggt
atggagcactcatttaagaacttttatctgttcaagaaagggaagagatg
gagcagaaggtagaatgggaggtatgatcaaaactgtgtttttttctgtgt
caaaggagagattacctgttcatgtgtagatgcaaatgagagaagaaagg
caagtattatgaATATCTAGTGCTTTGGGAGGCAGAAAAGATATGGGAAC
TATAGGTAGAGTAGCACATTTTAGGAAGAGTGACTAAAGGAGATCGAATT
TTCCTCTTAGACTCTAGGAAGGGTAAGATGACTGGTGAAGAGATAAAAAT
GTATTTAGGTGTTAAAAAACTTACACCATTAAGTTCCAGTAAAGTTAATG
AGATGAGGAAGCATAGAGATTGTTTTGAATAGCCATCTATTCATTTGTTT

GTTTATTTATAGAATATTGAATACATTACTTTGGTAGAGATACAAACATG
AAGAGGCTACCAAATAAATTTTATGTCtttattttattttattttaatat
ttttgagacagagtctggctctgtcactcaggctggagtgcaggggtgta
atctcggctcacctcaacctgtgcctcctgggttcaagtgattcttgtgc
ctcagcctccccagttcctgggattacaggcgtgcactaccatgcctggc
taatttttatatttttaAAAtttatttatttattttgatacagggtct
cgctctgtcacccagtctggagtgcagtggctcaatcttggctcagtgca
acctccaccttccaggttcaagtgattcttgtgcctcagcctcccaagtt
gttggcattacagacatgcaccaccacacctggctaattttgtattttt
agtagagacagggtttcaccatgttgcccaggccagtcaagctcctggcc
tcaagtgatcctctcacctcggcctcccaaagtgctaggattacaggcat
gagcctcagtacccggTTGCTACCAAATGAATTTATAGAGAGAAGTTTAT
TCATCtttctttccttttttttttttttttttcttttttAATAGTGGTT
ATTCGCAGCCTGAGTGGGCAGGGAAGAAGTAGACTCTGGGGCCTATCTAG
CATTATAGATTGGCATCCATGAGTGTCTAAGAGGTCAGCACAATTAGGTA
GTGGTGAAGGTGGCTTGGAAATAGTTAACTCTGGCCTGGGCTGGATAGGG
AATCCAAGGTGCTAGGAAACTGAATGGACTTCTTTCAAGGTAGAGAGCAA
CCTGAAGGTGGAGGTTTGGCCAGGGCGATGTAATAAAAGAGGTAAAGGAA
GGATATTATGATAGGAGGAGCTTGCCACGAAATAGAATTCAGTACACTTG
ATGGGGAAAAGGAGGTTAGGTTTGCTAGGTCAAATAACAAAAATTATAGA
CACAGTATGAATGTTAAAAGAGATCATGTTTATTGGCAGAGTCACAAGTC
ATATTTTTATCTGTACGTTTTACATACTGTGATACAAAAATCAGACATGG
TGAGTTTTTAAAAAGTAATAGGTTTTGTAACGTCAGTGAGCACCATTTTC
AGTATCTTGAGAATAACATTGTGGGTGTGTACTGTTTTTCGCAATCTTTC
TGAAAATCTCAATTTCACTTGAATGTTCATATCTATTAAGATGTGCAGTG
TGATACTTTCAATCTTTCAAGAAATGTGAAGAATTGTTTTTATTGATACG
TGGTATGTGTACAGAGGTAATTTAATAATAATGGTGTAAATATCTAAAGG
TTTTAGAGTTAGTATAATAAACCAAGCAGAAAAAGTGCTCATCATTTAAA
AGGCTTTACTTCTCTGGGTACATTACATCCATTGAGTATAATGTCTTGGT
GTGTATTTATTAGTATCATTACTTTGTTAGGATTAACAAATGTAGCAGGT
ATTTTGATGGACAGTATTAGAAATATTTTGTTTCTGTGTCTCTTTGCTCA
CTCACTGCAGGGAACTTCATTCTATCTGCAGACACACTGCTAATCTGATG
ATTTCTGTTTACATTTGCTTAAAGAACAATTAACTTTCTGTGAACTGAAA
GAATGGCTGCATTTTTCACAATATATCTGTGAAAAATTGATGGCTACTTT
ACAGTAGTAAGAAACACATGTTTTTTTTAATTGGGAAACCTTGGATTCTT
ACGGTCAAAAtgactagatatcctggtttgtctggggcagtcctggttta
ttcctgttgtcccagcatcctattcaattatcactcctcttcactctcag
aagttttctcatttggatgatacgttatatggtcattctaCGTGTGGGAC
ACATTTTGAGGTATATGGTCTACACTTTGAATCATAAGGGGAAGATACAC
CTTAGTAGTTGAAAGAAGCAGCCAGTAGTTGGTTTTGCCTTAAGAGGTTT
AGAGATACTTGAACAAAATTGTTCCTTTCTCCAACTTTCTAGAAAATACA
TTTTAAAATGTATTACAACTTGTACCCAGTTTGGTGGTTACTATTTAAAA
TCATCAGGTATGTTATGGTACAATATTTAACCAGGGAGTAACAGCCTTTC
AAATGAATGCATCCTTAATACCTTCTGCTTTGAGAAAGTGAGAAATATGG
TAGTGTTGGGCCTTGGATGAAATATTGGGTGTGAGATGTTTATCTAACAA
TGACTCAAATCTTGATTGTTTTAATTTCTTCAAACAGTACTAACATTCTG exon 2
AGAGAGCCTGTGTGTTTAGGAGGATGTGAGCACATCTTCTGTAGGGTAAGT BARD1_e02
AATTACGGTTTGATGTATATAGTACAACTGTATTTTTTACTAGATAACAA
TCATGTAATCTTGTTGATAATAATGGTACTTGATGTTTGTGTAACTTTCA
AAGTCTGCAAAGTAACCTATTGTACATTATCTTAGTTTGATCTTTAAAAC
TGTGTcttaatttacaaataagaaaaccaaagctcaaagaggatgacttg
tccaagttacaaagcttagtagacagctTTGCCAAATTGGAGGAAAAAAA
TTAATGCCTTTTATATAATTCAAATAGATGTTTTTAAATTTTCCAGTTAA
ATTTTGAATCTAGACTCAAAATTGTGAAAGTATAGGTCTTACAGTTATta
tattagtttcctaaggctgccataacaaagaaccaaaaactgggtggctt
agaacaacagaaatgtattgtctctctagttctgtaggctaggagtctaa
gatcaagatgatgacagggttggttccttctgtgggctgtgagggagaat
ctgctccttgcctttcccctagcaatctttgctgttccttgacttggaga
tgtatcactctggtctttgctttcatgtacacctggcattcttccttgag
tgcttgcttctctctgtgtcaaatttcctcttttcatacgaataccactt
atattaagggctcaccctaatgttctcatcttaacttgatcgtctagagc
ctctttttccaaattaggtcacattcacagtaactgaggggtgaagactc
aaacatctttttggggacacaattcagtgcataacaGTTATTGTGAAAT
TATATCCATGTGATGGCCCTGGCTTACAGGTCAGAAGATTAGATTTTTAT

CTCTTATTTCTTGTTCAGGAGAAATCAGTTGAGAGATTAAGTGTCTTAGT
TTAATTGCCTATGAGACAGGGAAAATATAGTCTCCTTTGAATTAATCTTT
TAATTATTTCCAGTTATGAGTATGTTACTGTCTGACATGAACAAATACAT
TCGGAAATTTGAGCATATATGAAAATAACCTTGTGTATTACTTCAAGGGC
TAAGGTTGTCTGGGAGGCAGCTATGTTTTGGGTCCCAATTTTGTTCCTGG
AAAAACAGATCTGTTATACGAGGGATATCCAGCAGGAGGATGGGGGGTAG
GAGATGGAACCTGTtgcctaccagatacgtctgtttggatgatgaaagaa
aaggcattttaaagttggtgtgttcagagctgacctcttgatcatcctcc
tcttcagccttttcctgcctgggtaatcttcatcacagtaaatggtacca
gttactgctaggttgcttatcccacatcgctaagttctgtcagttctgcc
tcaaaatgtgtcctgaatcttgaattttccacctttttctgaattttc
tacagtttatagtttgttccaagccatcattgtattatctctgctcatac
tattgtgataggttcatagctggtcttcccttttggcatcatcacccct
tcctcattttctacattcttgacagtgatctttgaaaaatcacgataata
tccatatcattaccctgcttaaactctttagtgggttcctattgcagtta
aaataaaatccaagctctgccctctggtctgcaaaaccctgtatgggacc
tagaacctgtcttcctcttggacgtcatttacctctggctcacactgttc
cagcacttctcctttagctcattcaatacaccaaattcactcccgcccc
aggactttggcacttgctatttcttccacgtgaaatgtacttatgccaga
tttctgtgaggcttactttttaccagttatatatcagctgaaatgtcact
gcctccaaaacgcctcccatggatctgcttacgaaatgatgcccactccc
tgtccccaCTCTGTAGCAAGCAGATGTATTTTGTTTCAGCTGACAGATGA
TGGGTTTGCTCACTGTATACAATAGGTCAGTCACAAAGCTGCTAGAGCAT
AATGAACTCCATGACATTTTATGCAAAGTTGGTTTGTGTGTGTGGTGGGG
TGGGGGATCCATACGTTTAATCAGATTCTAAAGGACTCTGTGACTTAAAA
CAACCAATCAAAGGTTTGAGACACGAGAACATCTTAAAAGAGAAAATGAT
TATGTGTATATGACATGAACGTAGGAAAGAATTCATTCAGTGCAGTTTTG
ATTTGTTTTGATTATATGATGCCAAAAATTATGGTAGCTGTTTTTATTCA
TGCAGATTTTGAGTTAAAACTGCTCAGCAATGAGGTTTTAAAATGATTTG
ACATAGCTCAGTTCAACTGATAAAGGTAATTCATCTACTCTCTAAGATAC
AATTTAAGACATGTGGcaggggtgctcaaacattttgagcattcttcaga
attgagaaccataaagagcttttgtgtgttcatgcatttaccgtacaaaa
ctgagacttttaaaaaaattaatttaaaaataataaacccattaataca
aacacaaataacactttgtaaggaaaaataacaattttaacaaatcaaag
aaaaaaatagagtggcactgttgtacattttgcaaatctctgtcttgct
gaatggaaaacagctacattctcaacttatatattcagtctattgtgata
ctttttgattgaagaaaatctggctttatacataattgtagttggaaga
agtattgaagtattttcagtagctttttagataattgtggctattcttc
tttaatactacaccaaaacttgacaagtgctttttattttttatttttt
gtgaagacaagagtctcactctgtcacccaggctggagtgcagtggcgcg
atcatggctcactacagcctcaacttcttggcttgggtgatcatccaatc
tcactttctgagtagctgagactacaggtgcaccccaccacgcctggcta
atttttgtagtttttgtagagacaaggttttgccatgttgcccatgatg
gtcttgaactcctgggctcaagcgattctcttgtctcggcctcccaaagt
gctgagattacaggaatgaatcaccatgcctggccaacaagtgctgtttt
ctttaggttcatcacagtgtggaatctgaaactatatcaataaattttta
tactctgttacatgaaatttcattggtttatctagtactttgaatttatc
tgttactcagcatgattttatgacatcacacacgggtcatttgagaaata
ctcactgagctatacaggtccaccgaaaaatgacatttttTTCAGAATTC
CAAATTAATGTATATAAAATAATTTGGGAATAGTTTATAAAATGTTATAG
AAATATAAAATACTAGTCTGATCTGAGTACTAGTGCAGTGCtggacaagt
aacttaaataactggatctcattttcttcatccaaaaatgaggtggtagt
tcttgatgaGTTGATTTCAGATTGTATTTTACTGATAATTAAGTATTGCA
CAAATGATTTAAATTGCATGAGTAATCAGTTTTACATATTTTTTTGTGTT
GGGGTTCCAAGTTAGAGTTCTTAACTACTAGCAAACAAATGTACAGAAGA
TCCTTTGCTAAAGAAAGTTGACATTTATTGACCCCAGTGACATTTTttga
attagatggtcccaaaagtctcttccagctctggtatGGGTTACAGATTC
ATTTTACAATTTTTTTTGAGTTACATTCTGTCAGAAATCATGCTAGAAGC
CAAGGATACAAGAATTAGAAATGGCATAGGTTTTGTTTGAGGATAGTTCA
TATTGAGTAAGAATCCTCTCTGCCTACAGAGGATTGGGTCCTGTGACAAG
GAATGTCCTGTGGTGCTTGGGGAAGATGTGGCTTTTCAACTGTTACATTA
CTTACTCAGTCCTACTGGAACCCATCTGGTGAGGCCAATGAAGGAGGAGA
TTTATAGAATTCTTATTCTGGAATTCACAGATGGGCTTGTGGGGGGGATC
GTGAATCCCTCTTTTTACATGAGTATGTATAGATTTTCATCAGATTTGCA

AAAGGTCTATAAACCCCAAAATTAGAAAAACTCTTCTAACTCTGAAACTT
TAGTCCTTAGATAACCCAGGTAATTGAGTCTACAGGTTTTAAAATTGTCT
GAAAAAGTCAAAGATCTTTTCCCAAAAGCTACTTTAAAGCCTTGAGATAC
TAGTCCCAAAACAAAACAGAAGGTCTTTCCCAGTGTCTGCAGTAGTTTTG
GGTATTTTCATGCAATGTTAAGATAGAAAAAGTTAGGATGCACGACTACT
ATGCATTAGGCATTCTATCTTTCTGTTACATCTCTGTAACCTTTAGAAGC
TACAGATTTATTTGTAGGGAAAATTATGCAGACTAATAATCAGGCTAAGT
AAAGTCTCTTCATAGCAAATTACATGAGCAACCTTAGATTTGATGTATGT
ATTTTACTCTTTAAAACAGTATTCAACAAGGATATTACAATTGACCATTG
TATGTTAGAATAACCTCTGCTCCATTTATTTCTGTTCAAACTGTTTAGTT
TTTGGAATTAAATTCTGCTGAATGGGTTGCtttttttttttttttaatt
atttaaaaGTAATTGTGTAAGTGACTGCATTGGAACTGGATGTCCAGTGT exon 3
GTTACACCCCGGCCTGGATACAAGACTTGAAGATAAATAGACAACTGGAC BARD1_e03
AGCATGATTCAACTTTGTAGTAAGCTTCGAAATTTGCTACATGACAATGA
GCTGTCAGGTAAGAACTATCCCTATCTCTCTTAGTTAAATTCATCAGTTA
AAAACTGATGAATTCATATTCATAAAGTATATAAAACATCTATCTGGAGT
TCTGGAATACGTATTTCAGATTTTAAAATCTGTAGGTtttttttttttt
ttttaaatagccattgagtctctctatgttgtccaagccggacttgaact
cctgcactcaagggattccccccacctcagcctccctggtacatgcatgt
gacacacccggTGGTTTTTTGGAAGATCATTTTGagaaatgacgggaacc
atagagataattaaagtctaaccccttattttatagttgagaaatcaag
gtccagagaggtgaaataaccttgcctacagccgcatgtgtagttagtac
tagaagctggagaaataaagcccatttctgactgtaaatccaggaacttt
tttgctgctTTACATTGCCTGCTTTTTACATTTATGATAACTTCTGCAGA
ATATATATGGAATAGTGATTTTGGCCTTAATAGCACTTAACTCACTTAAA
TACACTTTCCCAAGATAGAATACGACATTTTTCCATGGATCACTTTTCTA
GACTGAATTAATTAGATGAGCACATTTTGAAAGAGCAGAAATCTAATATT
CATTTTCTTTTCTATTTAAGTGGGGGTTAACGTTTTTTAAATTGTCCTTA
GACTCTGTGTATTAACTGTGTATCTTTCCATTGTGCTTTCTGCTGAGAAC
TAATTAACTGTGGAAAGAAAGTAAAATGTTTTGTATCTTCATAATTGGAT
ATTAGAGTTGTCTTTTTATTGACCAGATCATGCTATTTTAGTGTGTGTTT
GTAGAAGAACGTGTTCTTGACTGGCAGGATGCCATGGATGATTGATAATG
CTCATATAAAATTGTTAGATTTCTATTTTTAAATCTTTTGTTCTTAGAAT
CCTGCCATGATGTCTTATTGTCAGCTAAAGATGAAGTTGATTATACAAAA
TAAATAAATGTGGCAAAAACTTTGAGTCTTACCACAGGTCCATATTTTTA
AGAATTAGAACTTAAATCACTTTATACCTATTAAAATTTTCCTTCAGCTA
GTTATCTGCGTCATTGTATTCAACTCCATTCTTTTATGATAAAATGTGCT
GTAGTGCAGAAGTTTTTCTTACTTTCAGATGTAACTATACACACACATTT
TTAAAAGTTGCCGTTTTTTAAAAATGATATTGTAGTTGTAAACCTTTTTA
AGAACACACTGAAGAAAAATTCTGTCATTAGTTCATCTGAACTCTTCTTA
TTTAAAGACAGTACTGACAATGTTTATTTTCTAGTTAAAAGAGATTGGTG
TGTGATCTACCTTTCTCTCCCCACATAGATACCGAGGGTCAGTGCAGGGG
TTCTTAATCAAGGGCCCATGCACCTTTGGCAGTGTTCAGAGTTGAAGTGG
GTAAGGGATCAGTGAGCTTCTTGAGTTAATTGTGCAGTATGAAAACTGTT
CATGTTTGTGTGAAAGTGACTTTTGTTAGATTCTTAGGAGTGTGTGGTCT
GTTAGGATTATTGGCTTTGCACTGAAATTTGGAGTGCTACTTTGGGATTT
GTGGCTATGATGATATTTTGATTTGGTTCTTCACCAGTTGCTGTATAGTA
Atgtattcatttgctagaactgctgtaacaaagtatcacaaactgagtgg
cttaaacaacagatatttattgtctcacaattccagacactggaagtctg
agatgaaagtgtatgcaaggttggttctttgttgaggtctgtgagacaga
acctgttttaggccagtctccttgtcttgtagatggccatcattatgtta
acatggttttctccttgtttgtgtcaaatctcccccttttaaaggacag
gagtcatattgcgttgggagtccaccctactccagtatcgcttcatttta
gctagttacatctgcaatgaccttatttccaagtaaagtcacattctaag
ttactgggggttaggacctcaacatgtgaattttggtgggggaggaaca
caatttaTAACAATATAACATTTTTAATGATGATAAATGGAGAGCAGGGT
AACCCTTGATGGATTTACCGTTGTCAGTTATGGCATACCATAGGGGTGCA
AAAAAGAGTAAGATAGACTCCTTTCCAGAAATTTTATTCTAGCTAGTGAT
GTTTGAATCATTTATATTTTGAGGACTATGGAATATGAAATGCTTTCTTC
ATATTTCATCATTAAAGATATTTAGGAAAATTGAGTTTAGAACAAGTTTC
TGAGGCCTTTAAGAATGCGGTCTTTCTCATCTAAGTAGAGTTGGAGATTG
GAGAGATAATTCTACTGATTAATTTCCCAAAGGCCATTTTATGTTTGTTT
TCCTTATTATAATTAGGAATAATTATTTTAGTCCTTACCAGACTTAAGCA
CTGCGCCCTCAACGTTACTTATATTAAACTGAGTTGAAGTTCCCTGTTTA

GATAACacataatcagaaatttaggaacacgaaggtcatctttaatcccg
ttattctgtaggtaaggaaactgaggacccaggctacacagggcagttgt
gttgtaaatttcttACTGTTTTTCTATTTTACACAACTTTTTGGTTCGAA
GTGTAGGTATTCATTATGTTTGTTGAATTGAGTTGGATAAAAATTGGCAG
TGCTAATTTATATGCTAGAGACTAAACttaattaaaattatttatttatt
tattttgagacagacagtgtcttgctctgttgcccaggatggagtgcatt
ggggccatcatggctcactgaagccttgaactcctgggttcaagagatcc
tcccacttcagcctcccaagtagctacgactacaggcactcaccaccaca
taccctgccaattaatttttttttttttttagaagagatagggtgataggg
tcttgctgtgttgcccaggctggtcctgaactcctggcttaaatgattct
cccacctcgacctcccaaagtgctgggatttacaggcatgagctaattgt
gcctgacctaattaaaataattttaaaataCATTCTTAGAATTTGGTTTA
AATAATTTCATTTCATAATGATAACATCTATGCATTCATAGACCTTCTTA
AAAAATTATAATGGTAGTATCTTTCTTTGCTACTTTGATAGGAAATGACA
ATAAATTTAGAGTATAAACATACTGAATTATAACCTTCTGTCCAGATCTC
TAAATTCCATGGTGTAGAGCTTTTTCTACTTTAATATAAGTTGTATAGAC
CCCCAACTTTTCATCCCAAATTCTTCCCAAAACAAACTAGCCTTGAAGGG
CATTTGAGATTTTTTTCTGGAACATTTCCCTTTGTTTCTTTATAGTTTGT
CTATTCAGTGTATTTTACGCAGTACCTTTGTGGTAATTGATTATCTAGTA
AGTACTTCTAGAACTAAAAGGTTAGAAGGTAAACTATATACCAGGAACTG
ATAGTTGGGGACAAATGACAGTTGGTCTTCTTTATAATAGTAAGGAGATT
GGAAAATGATctcattaggataagggtattagattaagtgattcctaagc
tgtctctccaaaattgtatgattctgtTAAATGGTCATGTGTAGAAGATG
ATGAATTAAGTGGAATGGTGAACATCTCTCACATTTTCTCTTTTAAAAAC
CCTGTAGTTCACAATTTATCTTTCAAATGAACCTTGTTACTTGCATTTAC
TATAATATATATAATTACTGTAATTAAGGTGGGCAATTAGGAAGAACTTG
TCAAATCATGGACATAGTGTATGGTATACTGGAGATAAAGAAGCCTAATT
TGTAGTAATACTTTCTAAAATGAGCTTCTAAATACTTGAACAGATTTTTC
TTGGTTCAGTGTACTTTAAAGAGGAGATTGTCAAATTTTGCTGACAACCA
TTTATGGATAATTTTACCTTATCCTTTATAACATGATCTTCTTTTTACTA
ATGGAGTTAAATTTAAAAGTTTTTATTTTCTCTTCAACCCTATCATATTA
AGTCAGACCTTGCAGGTGCTGGTCAGGTTCTGTGAAACATGTCGGTGTTG
GTGACATTAAAAAAAAAAATACTTTGATTATGCTTTTATAGTATTACAATT
TCCTTTTCTATTTTCTGCTTTAGAGAGAGATGCTCGTCTTTCTGTAACGA
TTACATAGTGTTTCATATATGCTATGACTTTTTTATAATAGAGGAAGATG
AATGTTTGAATAGCTGTGTTGCTGTTGTTTTTCTTAAATAGTACTAAATG
CTTCCTAGCTTACAAAATAATCATTCTTCAGTGAAGGCGTTAATGTTGCA
CAGATGGTAGCAAAGGACTTTCCTTTGATTTTTAATAAGGGACTGGTTCG
TCAAAGAGATTTCTCAACTTCTGTTGTGAAACCTATAATAGAGAATAACT
TGAAAGGCACACAGTGTACATGATTATAATCCTTTGATGTAGATTAATGA
TAGCTAAATCACATTTTCTAATTGCCTCTTTTTTCTATAGTTGATTATTA
AATTGTAAGAAAAAACTTGGCCAAGCAGCTCCTTAGTAACACATGCATCT
CTGTTACTCAATATTTTGTGATGTACTCTGAAATTCAGAGAATGGAATTC
TTTTAAATATTATCATTAAGGAATTTTCTGTGCAGAGGAAGGACCAGTAT
TTTTCTTCATGAAATGAATATGTTGATTTATCATGCTGAGAAGCATTCAC
CCATAAAAATTGTTGGTAGCTGTTAATAATGTGATTTATGTTCCAGCAAT
AGTGTTTTGCTTTTTACAGTTTTATCTTAGAATATTATCTTAGATATTCA
GAATATCTAATAAAATTCAGAATATACAGTCATCTTTGTTGTGCTCCTTG
CTTAGTTATGCAAGATACATAATTAAAATCTTTTGAGTAAAGTTATTATT
ACCAGATGAATAGTAAAATTGTGTTCATATCAATTACATTGAGTATATTA
TCTTCATTTACTCATTTATAAGGTAGGAAATATGTACACCTGAGTATGAA
ATTTCTTCTTTTTATATTGAGGTTTTTTAGTCAGTTGAGTATGTTGCTGC
TATATATAAGCCTTAATTTTCCTTTAAAAGCTCAATTCTACATTTTTAAA
GTTTTTCAAAAATTGAGAACCTCAGGGTTTGAACATGGTTTTAATTGAAA
ATGTCATCTGTGtttaaaataattaaaaaaaaatCAAATACAAGTTATTA
CAGACTATCAGAGGATTAACCTTTATGACTTTGTAATCCTGAGTCTTTCT
TGAAAAGTAATCCTGGTAACATTTCATTTTGACCTTTCCTATAGACTTGA
GATTATATCAGAAGTGACTTGGTTTTGAAATCTACTTACCACTGCAAAGA
AAATACCTTGTTGAATCTAGACATAAAGTCTCTATGTGAATGGATACGTC
ATTCTGATACTTTCCAAGATCCTGTCTTAATAACACTTTGTGGACCAGGT
CATGTTCAGCTATGATATTTAAAAGTTTATGTAGGAGAATCTGCCCTTAT
TCATATACATACTCCTGCCTTCAGTAGGTTTATCATTTTACTGTCTTTTC
AGTGTAGTGCTTCCATTTCATCTGCAGGAAGTGATGGTACACTTTGGAGT
AGAAAATCTTTACTCTTTTGCTAGAGAGGGACCATGTCTGAAATTTCTAG

AGTTAAGTACTAATTATTTTATTTTTCAAAGCCTGTTTCCCATTTCTTCT
TACCTGTTTAAGGTTCTTTTACCAGGTAAATATATTTTTGAAGATAGGAC
TTTTGTACTCAAGAAATATGAGGTGAAAGAATAGGCAGAAGAAGCAATTT
TGAACTTGAAAGAATTGAGATGATCATTAACAATCTTCTGGTGTATTAAT
AATTTTAAGCTGGTAAAACAATAAAACAAGCTGTTCTATGTGGATAAAAA
ATGACTAAAAGGAAATAAAAAATAATAGATGATGTACTTTTCTTTTGCTA
ATAAAGACATGAGAATGTTGAAAAGAAATTTATTCCCCAAAACGTAAGAA
AATGAAAATAATGTTGTATCTCTTAAGTATTAAAAGTTCTCAGTAATAAT
CACCATTTGAAAATGTTATTGAACACCTTGTAACATCAGATGTTTATTTC
CTATATGGTAGATGTTATATCCCTTAGGAATAAGACTAGTGGCCACTGGT
TTTCTTCTTAAAGTACCTTAGGTTTAGAACTCCATTCCATTAAAAGTTTT
CTTTGGTTCTGTAATGTAGGTAAAGTTTTATGCAGAGTTCTTTTCGATTT
GAATGAAGAGTGACTTAAGGAGGAAAACAAAATTTGGGTAGTTtcttttt
ctctcttttctctgtctctctctcttctctttctTTTTAAAATAGGA
ATAACAAAACATATTGTAAGAAGCCTGGTTGGTCAGAGGAGATTGTCTGT
GTTAATTAGGAGAATGTTTGTCAGGCAGCATGAAATCATTTGTTAATTAC
TGTTTTTAATCTTATCAGATTATTGTTATTTTTCCAACTTTCCTCCGTTT
CTAATGttcttctttttctttctttctttcttttttTTCTGGGCCTGAA
ATTACCTTTTGCTAATCCAGTTTGTGTGGAGAGATAGAGTGCCAGATTGG
GAGttaggggactccttccttctttttgttttaactttagtccttcattg
ttaaaatgaaagaattagactaaattattttcaagttttcttctagctct
taaaattaattctaACATCTCTTACGTAAACATTTGGAAAGATTTAAGAG
ATATCAAATAatttattcactagtacttactgagtactgtctattcatgt
gctcaccactgtgctgggctgagctgggTTGATTTTACCTATGTTGTCAT
GGGGTGGGAGAGAATTTTAAGGCAATTTTTTTTTCTTGAAGAAAATGAAT
ATCCCAAAGACAAAAACAACATAAGACAACTTGAAGGTAGTTGATCGTCA
AAATCAGAAAAGTGGATGACTTGTATGATTTGTCAATgaagaaaaacttt
tcctctacactcttaggttcattgtatggaggcctgtgaattaaactgta
aaacaattagcaagagaaaaaatagattttatttaTGAATATACTTGTG
AGTTCACAGAAAAATGTGACTCTCCAAACAGGTAAAAATGGGAGTTTATA
AACCTAAATTAAGGGGAAGAGGTAGAGGGAAGAAAAGGCTTCTATGGAAA
GAACAAATGGCTTTCTTTCTtttttttttttttaactataatttctggga
tacatgtgcagaacatgcaggtttgttacataggtttacacatgccgtgg
tggtttgctgcacccatcaacctgtcatgtacattaggtatttctcctaa
tgctatccctcccctgaccccgctccatcggacaggccccggtgtgtgat
gttcccctccctctgtccatgtgttctcattgttcacctcccacttatga
gtgagaacatgcagtgttgggttttctgttcctgtgttagtttgctgaga
atgatggtttccagcttcatccatgtccctgcaaagtacatgaactcatt
ctttttatgggtgtatagtattccatggtgtatatgtgccacattttctt
tatccagtctgtcattgatgggcatttgggttggttccaagtctttgcta
ttgtgaacagtgctacaataaacatacatgtgcatgtgtctttatagtag
aatgatttataatcctttgggtatatacccagtaatgagattgctgggtc
aaatggtatttctggtcctagatccttgaggaattgccacactgtcttcc
acaatggttgaactaatttacacccccaccaacagtgtaaatgtgtttct
atttctccacatcctctccagcatctgttgtttccatgacttttaatga
tcgccattctaactggcgtgagatgatatctcattgtggttttgatttgc
atttgtctaatgatgatgatgagcttttttttcgtgtttgttggccacat
aaatgtcttcttttgagaagtatctgtttatatccttcgcccactttttg
atggggttgatttttttcttgtaaatttgtttaagttctttgtagattctg
gatattagtcctttgtcagatgtatagattgcaaaaattttctcccatt
ctgtaggttgcctcttcactctgatgatagtttcttttgctgtgcagaag
gtctttagtttaattagatcccatttgtcaatttggcttttactgccat
tgcagtaaaagacttcaggacattgttttagtcttgaagtctttgcccat
gcccatgtcctgaatggtattgcctagattttcttctagcatttttatgg
ttttaggtcttatgtttaagtctttaatccatgttgagttaatttttata
taaggtgtaaggaaggggtccagtttcagttttctgcatatggctagcca
gttttcccaacatcatttattaaatagggaatcctttccccattgcttgt
tttgttaggtttgtcaaaaatcagatggttgtagatgtgtggcattattt
ctgagggctttgttctgttccattggtctatatatctatgtcacaatggt
ttgccgcacctatcaacccgtcttctaggttttaagacccacatgcatta
ggtatttgtcctaatgctctcactccccttgcccccaaccccctgacagg
ccctggtgtgtgatgttctcctccctgtgtccatgtcttcccattgttta
acttccacttataagtgagaagatgtggtttttggttttctgttcctgtg
ttagtttgctgagaatgatggcttccagcattatccatgtccctgcaaag

gacatgaagtcattctttttatggctgcatagtattccatggtgtatat
gtgccacatttcttta AAAATCACTTTCTTCCGTGGTCTCTGTAAGTGA
GGTATACTGGCCATCCTTTAATTCCTTAAACTTACTAcagttgccccttg
aataacataggcttgagctgcatgggtccacttatatacagattttttt
ggtcaaatgcagatggaaaatacagtattcatggaatgcaaaacccaagt
atacagagggctgacttttctacactctgcttctccaggagtgagtaca
ggaattgagtgtgtgggtatttggtatccaagggagttctcgaaccagt
cccctgataatatggaggaacaactTTGTATGCTCTCTGCTATTGGGCTT
TTGAACATTTTATCATCTTGCTCTTGCCTCTTGGTCTAGGTAACTTGTGA
TCTTTTAGAATGctttttaccatttctattactgtaacacttgtcagagc
caatatatttcctcttggatgatagccacataattggctattgtttctac
tcttttctttccaaagttcctcctccacatggcagtcagagtggtaattc
tacaatataaaacattatatcccttttctgcATTGGATGTgagatccaca
ggactgtttcctttacatcatttacatctcagctcaaaaatcaacttctc
cttgatgccttccttgatgaccttaattaaagtagcaGGTGAGTGTAAAG
AAGAGTATCTCTCACAAAATCTGGCACTTTGTGTACATAGAGACAATTTT
TTTTCTTTTTAAAAACTCTGCTGAATCTTTGTATATTCTGTCTACCCTCA
ATGGAATCTTTGTGTTTTAATTCTTGattataaatctatattttcaaat
ttttaaaaagtattttatatatatatatgtagaaaaaatatatattttCT
AACCATATAGTTTAATGAATTTGCACAAACTGAACTCACCcagaacaaaa
acaatactctggaagcccctcttgtgctccctttcagtcactacttttcc
ctgtaggggtaaccaactactattctgacttaacacattaaatcagtttt
gactgttttatatttgatgtaagtgaaaacatataatgtgtatgtagttt
attttgcttaagagattatgtttgAATATGTAGTTATTGTTCATTTATTC
TTACTAATCTTTGGTTATACAGCTTGACGTTAATATTAGATGATACCTAA
AGGGTCATGACTATTGTCTTTAATCCCCTTTATTCATTTTGTCAGAGAta
cgttttgaagtcatatagtatgagtcctccaacttggttcttcagtatta
tgttggttgttctaagtcttttgcctttcaaattgtacctttctttttcc
gtgtaaattttataattgcttgtcgagttttacaaacaagcctactggaa
attacattgggatttgttatatctgtaaatctgtttggaacaattggcat
cttaacaatattgttatagttcatgaacatggtatatctctccatttgtg
tagaattctttaataagtatttgtaattcttagcaaacagaacttatac
attctgttagatttgtattttatgggtttttttgggtactaTATTTGGAA
TATAAGTTTCAtgttttgttctctgctatatcttcagtctctaggatatg
gcacaaagtcggattcagtaagtattgaatgagtgaatAT*CTGCTATCAA*
*AGAGTTCACAC*TCTAGGAGCTGAGAAAGAAGTACATAATTAAAAGATGAT
ACACTTTAGGGGAACTGTAAACAAAATTCTTCGGGAGCTCCATGTGGGAG
CAATAAATTTCATGTAACAGATTTCTTTTTCTTTTTTTCTGTCAGATTTG exon 4
AAAGAAGATAAACCTAGGAAAAGTTTGTTTAATGATGCAGGAAACAAGAA
GAATTCAATTAAAATGTGGTTTAGCCCTCGAAGTAAGAAAGTCAGATATG BARD1_e04.1
TTGTGAGTAAAGCTTCAGTGCAAACCCAGCCTGCAATAAAAAAAGATGCA
AGTGCTCAGCAAGACTCATATGAATTTGTTTCCCCAAGTCCTCCTGCAGA
TGTTTCTGAGAGGGCTAAAAAGGCTTCTGCAAGATCTGGAAAAAAGCAAA
AAAAGAAAACTTTAGCTGAAATCAACCAAAAATGGAATTTAGAGGCAGAA
AAAGAAGATGGTGAATTTGACTCCAAAGAGGAATCTAAGCAAAAGCTGGT
ATCCTTCTGTAGCCAACCATCTGTTATCTCCAGTCCTCAGATAAATGGTG
AAATAGACTTACTAGCAAGTGGCTCCTTGACAGAATCTGAATGTTTTGGA
AGTTTAACTGAAGTCTCTTTACCATTGGCTGAGCAAATAGAGTCTCCAGA
CACTAAGAGCAGGAATGAAGTAGTGACTCCTGAGAAGGTCTGCAAAAATT
ATCTTACATCTAAGAAATCTTTGCCATTAGAAAATAATGGAAAACGTGGC
CATCACAATAGACTTTCCAGTCCCATTTCTAAGAGATGTAGAACCAGCAT
TCTGAGCACCAGTGGAGATTTTGTTAAGCAAACGGTGCCCTCAGAAAATA
TACCATTGCCTGAATGTTCTTCACCACCTTCATGCAAACGTAAAGTTGGT
GGTACATCAGGGAGGAAAAACAGTAACATGTCCGATGAATTCATTAGTCT
TTCACCAGGTACACCACCTTCTACATTAAGTAGTTCAAGTTACAGGCGAG BARD1_e04.2
TGATGTCTAGTCCCTCAGCAATGAAGCTGTTGCCCAATATGGCTGTGAAA
AGAAATCATAGAGGAGAGACTTTGCTCCATATTGCTTCTATTAAGGTAGG
ATGCTTACTCTGAAATACCATCTCAGAATGAGGCCAACTATAAAGCAATT
TCTTTGCAGTTTTTGAAAAATGGCATAGGATTACTAGGATAATTAACCTT
TCACAGACATGATACTTCCTCTGAACCAGAGAAGCCAGATTCATAGGGAG
AGCATCTCTACTTCAGTTGGAGCAGTGGCCCCTGAGTCTGGGCGCATGAT
CTTGTAGGAGAAAACCAATATTTGAATATTTCGGCTTTTATTTTGCCAAG
TGCTTTTGCTTTTGTCTATTTTACCTTCAGTTTTTATCATTTTGTTTACC
TGTCTTCATGCTTTATGAATGTAGACAATTGCTAAGTTATTACAGGCAAC

AATGTTTACTTAGTAAAAAAGCCCATATTTACCATCCAAATTCAACCAAA
ATTTGGAAGGTTGAAAGATGTGGTCTGTACATTTCTCCAATGACCGGGAC
ATTTGACTATCAGAAATGGCTCCTCCAGTTCACCACAAAGGAGCTGCTTT
TTACCCTACAATCAGCTGTTCCTTTTACTGACCTGTTCATTGACGTTCAT
TCTCTTTAATTATTGCCTCAACACCTGAGATGTGGCAGCGTTTTATGGAA
CCTGGCCTGTTTTCTGAACACTTGGCAGTGGTTAATCTTTGAAACTGACA
CAAACACTACATGTGAAATAGAGCAGTAGGCACCATAGCTTTATAAGCTT
TGCTCTGTGATTTCATGCAAATGTTAGGAAAGTATGATATGGTAGACCTT
AGTACTAAATTAGGATAGCTTTTATGCAGTCTCAGCGACTGTACGTAGTC
AATTCACCCAGGAAATCCTTTGGTCTTCTGGATGGGTAGAGAACTTTGGG
ACACCTGGGAGGAACCACTTGGAGGTCCACAAACATGAGGCTGGTGGGCA
ACATAAAACATTTGAGAAGAACATGGAGTAGACATTGAGTGTGGTCCAAA
GATTCCTGTATTTCTGTGTGGAGAAAGGGAATAGCATAAACTGAGCTTAT
CCCAGCTGCTGCTGGTTTGTTTTCCTTGCTTTTCTTCCAGATTAATGAGG
AATAcagtcagctctgtgtatctgtgcattctatgtttatgggttcaacc
aactgcgaatcaaaaatatttgggagaaaagccaataacaatacgcaata
aaaaaatgcaaattaaaaaatatcgtataacaactatctacatagcattt
atattgtattaggtgttataagtaatttagagatgatttaaagtatactg
gaggatctctgtaggttatttgcaaatacatcattttatatgaggaatct
ggattttggtatctgctgggattctggaaccaatcccccacagatactga
gggatgactgTACTCTATTTCTGATACCAGTGAGGCAGGGACCTTACAAC
ATGACCACATATCCTTTGGACATGTTGAATTTTTGCACTTGGCTCTGGTT
GTGTTTTAGTTTGATTTTTGCAAAGCCTGACTTAACAAAATTAGACATTT
TTTGTCATCTCTGTTATATATATGTATATTATATGTACATATAAGCTATT
GTAAACAAAGTTATAGACATAAGTAGCTAGTGTAATGATTATTTTTGTTC
ATTTTGCTGGCTAATACATAGTAATTTATATATTTTGCTTTCTGGGAAAT
TTTTGGACTATCAGGCAAGCATTTACTTATATTGGTGGCGACTTTTTCTT
CTAACCATAAGAATAATTTATCTTTATTAATGAGGATCCCTAGGGTCTAG
GCcagagttttgtaagttctttcttaaaggtgcagttagagtaaatgttt
tcagctttgcaggccatgaaatctctatcatagcggttcagctctgcctt
cttagtgcaaaagtagccacaggcaatatgcaaactaataaaacttacgt
acaaaattgggtgactggcccataggctatagtttgcctactcttgGTCT
AGGCATGTGAAGAAACTGTAATAGAATATTAAGTCAGGATAAAATGATGG
CATGCTCCTGTCTTGGTGAATTTTCTCTATTAATACGTTCAAGATTACTT
CATATGAGTTTTGTCAGTTTGCTTAGAGTTTTGCCGGAATTATTAAATCC
ATTTTTATTTCCTTGAACTTAATTATTAAATGCAATTATTGTCACCAGTT
GTCAGATTATGTTATGGTACatttttaaaagttaatttttattgttttt
aaaatCACCAATTAATAGTGTCCTTTTAAATTTTTTTATGAGTTGAATGT
ATGCCTCTCGCCTTAGCTTTATTTTAATAAATATATCCTTAAAATCAGAT
TTGCttatataaatatatttatatatttttttCTTTTTTGGGGGTGGGGG
GAGGGGCGGgggacagagtcttgccctgtcacccaggctggagtgcagtg
gcacaatctcagctcactgcaacctccgcctcctgggttcaagcaatcct
tctgcttcagcttcccgagtagctgggattacaggcacctgccatcatgc
caggctactttttatatttttagtagagacggagtttcaccatgttggcc
aggctggtctcgaactcctcacctcaggtgatatgtcctcggcctcccaa
agtcttgggattacaggcatgagccaccatacctggccAATCTTTTTAGA
AACAGTTTTCATTTCATTCCTATCCTAATTTTCTCATTTGCAGCCAAGAA
TGAATAAATCTATGTGTACACATATTGCAATTCTGCTATCttatttatat
tttgcacccacctgcttcagaaaaggatttgaagtggctctaaagttaaa
gctactataaaatGGAACAAGGAGTTGGTAAAGAGGAAAGAGTTACTACA
ACTGGAGTGAATTTAGTTTTGGTGACCTTTAAGGCAGCCAAAGCAAATAA
GGGGAAGGACCAGGTTTTGTGCTGTCAAAAGATAATGCATCTTTTTTGGT
GGTGATTATTTATGTTTTGTACCCACCTACTTCAGAAAAGGATTTCAAGT
GGCCTAAAGTTGTTTAGACTTGCGTTGTATTGTTTCTTAAGCATGTTGGT
AAACTTGCAGTTTCTATAGTTTGAGCACATGTTTGCGCTGAGCATCCTTG
GTGTATGCACCGAGGGCAGAGAACCCAGCAGGAGTTTGTTCTCCTGCACT
AGTTACCTTCCCCCACTGCAGGTAGGGCTTTACTGATTGATTTCTGAGCT
TGGAAACATTATTAATAAACATCGTATAATACCTTGCCCCAAACTACCTC
TGGGCATCATCATTGGCCATAACCTTTCCATAGCTACCACCCTACTGGCA
GCTACTTCTCTTTTCTTGCCCCTACCACTtgcattaattttctattggtg
ccaaaacaaacttccacaaagttagtgattcaaagcaatgcaaattaatt
ttcttacagttctgtacacctgaagtctagtatgggtctcatcacactaa
aatcatgttgtcagctgttttcaggaggttccaggaaagacccatttcat
ttttgcttggatggttggcagggacttcataattcttgtggtaagactga

gatccagatttcttgctggttttcagctgagggttgtttccaggttctag
agactctcacattcctttagttcgtggtcctcttcttcatctcatccagc
agcagcagattgagtccttctcacatcttatctttctgacatagccagaa
agaccctctgcttttaagaattcatacgattaaattgggctacttgagaa
atgcggaataatctccccatctcaaggtgtgtccacttaattacactgca
gtgtccctttcaccacgtaaggtaacatagtcacaggttctggggattat
tcgtgtgtggacatttttggaggTCGTAatttttttgaccacaccactgaa
gtagcttcctaatttccctttgtatttcctgcagtcagttctctatgtaa
cagccagagtaatctgaccgaaatcaaaattatcatttctctgctcTCCT
ATCAGTAAGTCTTTTTTATACCCCTCTTCCTATTTGAATATCTTCATAAC
TCATGTTCCTATTTGAATGTCTGCATCAGCTATGTTAAACTGCTATTGAG
AACACTGTGTCATGAATGGGAGAGTTTATGAGAAAGCTTTGTGCGTAAGT
AGAAACTAAGATACTCATTGTTTATTCTTCTGATTTATTACCACAATTAC
TTTTGCACCAACCCAATAGAAAACAAAATACTAAGGATATAGAGAAAATT
TTTTATTTTCTATTATGTCAGCTTTGCTTGAGAATTTGTGCCATCCTGAC
CTTCCCCTTTTCTTCCCCCACACCCTTCTACCAAGTTCTCTAGCACAAAT
TCTTAAAATGGCAATGACTACTCATAATTTATGCCACAGCAATGACTTAC
TGTGGAATAGAAGAATGGCTTGAAGCTGGCATGCCATGATTTATTAGTGA
TGTAGACATAATAGATAATGTTTTGAATGCAACTTACAAGGCATGGTATG
CTTAGAACATAACAAAGGTGATGAAGATCCCCTGGAGAGAGAGAAGATCC
CTTGTGAGGAGTCTCATTTAGAGTTTAACTTTGTAGTTTATTATACTTGA
AGACAGTCATGTTATATTGTGTGTGAAATTATATATCCTTCATAGTAGCT
TCATGAGAAGGTAGCAAAGGAAACAAAAGCAGCCTTGGCATATGTGATAT
TTTCAAGAGTTTAATTTCTAAAACACCTCCTGGAGTCAGTATCTATTTTT
CTCCTCTGTCACTCACCACATATGTATAAGTCTGCTTTTGTAATACCAGT
AACTTATTGTGCCATTGACCAGGTTATCAGAATTATCTGTTAAATTAcag
gcatacctcatttaattgcacttcactttttttatgcttcgcagagagttt
tgtttttgtgtttttttggcaatcctgcattgagcaagtctgtcagtgctg
tttttccaacagcctatgctcagtgtgtctctgtgtcacattttggtaat
tctcacaatatttcagatattttcactattgttgtatctgttacggtgat
ttgtgatcagtgatctttgatgtaactattgtaattgctttggggcacca
caaaccctggccatgtaacatggcaaacttaattgataaatgtcttttct
gactattctgcttactgataattctcccatctctctccctttgaatccct
gtttcctgggacacaagaatattgaatttaggtaaactaacaaccctaca
atgtcctctgagttttcaagtggaaggaagagtcactcatctcttactttt
atataagaagctagaaatgatgagtgaggaaggcatgttgaaagttgaga
taggccaaaagctaggtctcttgccaaacagccaatttgtgaatgcaaag
aaaaaattctttaagtaatttaaaagagctaccctgttgaatacacaaat
gagatgagaagaaagcataaccgccttactgccgatatgaggagagttgg
aatggtctggatagatgatcaaaccaaccacatttccttaagcaaaaccc
tactccagagcaagatcctaaatctcttcaattctgtgaagactgagaga
ggtgaggaagctgcagaagaaaagttggaagctagcagaggttggttcat
gaagttgaaggaaaaaagctgtctccataatataacaatgcaagttgaaa
cagcaggtgctgatggagaaactgcagcaggttatccagaagatctagcc
aagataattatgagagtggcacactaaacaacagatttcagtgaagttga
aacagccttttgttggaagaatatgccatctgtgtctttcatagctagaa
aggagaagtcaatgcctggcttcaaaggacaggctttcttgttaggggct
aatgcagctggtgacttgaagtggaagccaatgctcatttaccatttaga
acaacaaagcctgggtgggagcacatcttgacagcatggtttgctaaata
ttttaagcctgctgttgagacctactgttcaggaaagatattcaaaatat
tactaattgacagtgccacctagtcacccaagtgttctgatgaagataag
gacattaatgctgttttcatgtctcccaacaaaacatcaattctgtagcc
catggatcaaggagtaactttactttcaagtcttgttacttaagcatttt
gtaaggctatagctgttgtagatgggtattccacagatgattctggaaat
gactcaccattctagatgccattaatatcattgatgattcatgggaggat
gtccaaatgttaacattaacagtagtttggaaaagagttgattacagttt
tcatggatgactttgagaggttcagaacttaagtggagaaagtaactata
gatgtagtagaaatagcaagataactagaattagaagtggagcctgaaga
cgtaactgaattgctgcagcctcatgatcaaacttcaatggatgaggcgt
tgtttgttatagatgagaaagaaagtggtttctgcagatggaatctactt
ctggtgaagatgttgtgaacactgttgaaatgacaacaaaggatttagaa
aattatatcatcttagttgataaaacagcagcagggtttgagaggattgg
ctccaatcttgggagaagttctacgcagataaaatgctaccaaacagcgt
cacatgctacagataaatctttcatgaaatgtcagtggatgtggcatact

tcattgtcctgatttaagaaactgtcacaaccacccaaaccttcagcaac
tacctgcttgctcagtcagcagccagcagcatcaaggcaagaccatccac
cagcaaaacaattgtcaccatttttagcaataaagttattttcaattaa
ggtctgttcatttttttttaaaaaacataatgctattgcacacttagtaga
ctacagtatagtgcacatataacttttatatgtgctgggaaaccagaaga
ttcgtgtgacttgttttattgcaatatttgctttatcgcagtgacctgga
actgaactgacagtatctccgagatatgcctgTATTTTATTCAGTAAATT
CTAAGAAGCTACAGAAATATTTAACATATTTTAGAGCTTCATTCCAGTTG
GTTCATTTTTGTTTGTTTGTTTTAATGGCATCTAATTGCCAAGAAGGGAA
GTCTTTGGTTCCTGGTCGGTTCATTTACATGGAAAGGCTTAACCATGCTA
GGCAAGTCATTTGTCTTATACTTAATTGTAACGTTCTGTATTGTTTTTAT
GAATATGTTTATTAATGGTATTTTAGAAGAGCTAACATTTATAATAAGGA
AAAGTTTTGTTTTTATGTGTGTGTTTTTGGAAAGTTAATTTATATTGTTG
GAATATGTGTGGATCTGCAGTATGTACAAATGTGTGAAGGATCTTTCCTT
TGATTGAAAGATTAGGAATAAAAGATTCAGTGGGACACACACACACTCTT
ATTTTCAGCCTTCAGTGTATTACTTGAAAATACCAAATTCATTTAAAATT
TTTAATAAGCCTTGGCAAATACTTTCCTTAGATAAATTATGTTTGCTGTG
GTTTAAGAAAAGTTCAATTATTCTTTCAAGTTGTGAGGAGCCCAAAAGTG
AGTACTTGAAAGTAATTTAAAGAAGCCAGAATCAAAACTTAATTTATTTC
CTGGCTACTTCATATCAGTTCAAGCTATTCCAGTTACTGTAGACTCTATC
TAATTATCATAATTTTATTTATGCGCATGACATTACATTTACATGAAGTA
GAAAGATGAAAATATGACAAAACTTTCAAGTACTAGAGAGGAGAAAGCAG
ATTGGTTATAAAAAGCAAGCCGTTTGCTAATCAGCTATACAGTTCAGGGG
TGGGGAGAATGTTAACAGAAGACACTTTGATAATATGAAGAGGTGACATG
AAAGAAAAATAGCAACTGTCTAACAACCTTAAAAGAGAGTCTACTTGTAT
TCTTTAAATTGTCACTCCGTTTCTGTTTGACTATTCCCTTGAGACTCTTC
CATTTCACAATTGTGTTTATATTATTTTTTAAGCTTCTTTAATATTTTTT
TTTTGCATTACCATGTAGAATTAGAATGGGGAAATCTTTGAGAAGGTTTT
TGTGTGTGAGTACTTTCATGTTAAAGATAGGGAATGTGTGTGGTTCTGGC
AGTTTATCTGCTCACTTGTTACAGGATTGTTTTGGAAAAAAAAAATTTGTA
TCTTGGCAGATATTCCTGCATTCATTTGTAACCGTTATGAATATAAAGCA
AAAGTTgcaggggctcacacctgtaatcccaataattttggaagctaagg
caggagattgcttgagtccattagtttgggcaacatagtgagaccccccat
ctctacaaaacaataaaaaaaattagctgggcatggtgatgtatgcctag
cagtagcatactgctgaggctgaggcaggagaattgcttgggcccaggag
cttgaagttacagtgagctatgatcatgccattgcactccagTTGCTAAA
TACATGATAACTTGCTCTAGAAGTAGAATTTGCACTGTTAAGAAATCTGG
GAAAATGTTGCTTTTTTTTTTTTTTTTTTTTCCTGAAACTGGGGTAATTAA
TTTGAAACCTACAATCCTTTTAGACCCATTGAGCATTTAAGGTCATTTTG
TTAATTTAGGAATATAAAAATGTATGTTGCATGAGAATTTACTTTTTTAG
AAAACTGAAGATTTGAGAAACAGCCCAGAGCACAGAAACAGAGACAATTA
GATATAAAAAGCAtttttttttttttctgaggtagagtctcgctctgtcgc
ccaggctggagtgcagtggtgtgatctcaactcactgcaacctccgcctc
ctgggttcaagcagttctcctgtctcagcctcctgagtagctaggattac
aggcgtgtaccaccatgcctggctaattttttgtgtttttaggagagacgg
gatttcaccatgttggctaggctgatctcaaactcctgacctcatgattt
gcctgcctcagcctcccaaagtgctgggattacaggtgtgagccaccacg
tctggccAGCaaaattttttaaaaagaaaaaaaaatcaaatattatattt
aGGAGAATTAGTGATTTCTACATTATCAAACCAACAGAGGAAAAAAATTT
CAGAAAAAAGTATTGGGAGAGGGGTTGGGTGGGGAGAGATTTATTCACTA
AAGAAGCACTCACGAAACTCATTGAACACAATGTTATGCATGTAACTTCT
GTCTATAGCTTTTTATTTGGTCTTTTTTGAGAGTATGCTCTGCTAGATAT
TTTTGCTTTATCGTCATGGACTTAGGTACTGTACCCTGCCCTGTTATCCT
AAAAGAACTGATTAGTTATATTTGCAGTGAGTGGTGATAATAATGTAATT
TCAAGAAAATTTTTACCTTCATCCCGCTGCATAAATGCCTCCTTCTTCCT
AAAGCCTGACATTGTTTTACAAAGGGACAGTATCCTTTATATTACAGCTA
GAGATTTTTAAAAAGGCTTTAAGAGTTAACGGATACTTAAAACGTAATAT
GTAATTAAATATAAATGATAGATGTCAGATCTTTCCACATTACATGAAGG
GCCAAATTTAAGAGACCCTGGTATTAGATAGCTGGAGGATTATTATCTCT
CACTTCTAGGAAAATAAAAAAGAAGAGTGGGAGGGCAGACTACCTGAGAC
CAGTTCCCCAGAACTCCTTTGCCACAGTCTACCTTCGCTGGAGGCTGGCA
GCAGAGGGAAGCTATTTTTTTTGTCTTACTTCAAGACAGCTTTAAAGTGA
CCTTAAAAGTCTACTCATTTATAGTAGGATTTAGTTGGATCATGACACAC
AAGACACAGAAAGTTCAAAACTGGAAGGGGTCTTCAATTTTAATGCTATT

TAATAATTGACGGTGTCTTAATATGAAAGTCGAATTAATATTGTCTGCCT
TCAGAGTATTTTCCTTGCCTTGTGCCTCATTTTAATTCACTTATTCCCCG
GATCTTGCTCATCTGATTTAAAAAGAGGTTGTGATTTCTAAAGTAAAGTT
ATTGTATTTCTTACTATTCCTCATCTGCCTTCTCCATTGCATCAGTGTGC
TACTGTTGCTACTGTTATAACATTGTTGCATAGCTCTCCAGAGTACTGGA
ATAGAGAAATAGAGAAATTGAAAACCACTTGTTAGAAAAGACTTGTAAGG
CTACATTACAGAAGCTCTATTTCGTATAGTTGAATTAGTTGATAATGGGT
GGTGATTTTAGTCTTGAACTGGGCGGGGAGCAGGGGGATAAAAAATTTAA
TGCTAATTCTGTGGGCTGATTTGAATAGGTTTGTACTGATCTCTTTTTTT
GCCATTTTAAACAGAGCAACTGTTGCTTCCTAGAAAATGTTGAACAAATA
AGGTGAATATAAACTAAATGTATTAGGATGGAGTAACTGCCAATAAAATG
GCAGTTTTTGGCTGTTTTTTAACTTTGGTTTCACTGAGTAGTCTTCTGGG
CTAAACAGTTTTGGTTGTTTTAAACAATACTCAGAAGCCATTTACAAATG
CTCAAAAATTGTTTTTTTCACAAGTGAAATTATGGCTTTTAAAACTTCAT
TGTGAGCACATGATCTTTGGCCAGTGTCCTTTATTTGGTACCTGAAATGA
ATTTTGAATATGGCTTTCTAATAGATTTTGTAGTCTATATTCAAGCATGA
CTGTACTTCTTGATATCTCAGAATAATTTATACGCAGACAAtttcttttt
tgttttgtttttttgagacggagtcttgctctgtggcccaggctggagtg
cagtgacatgatcttggctcactgcaacctctgcctcctgggctcaagtg
attctcctgcctcagcctcccaagtagctgggattacaggctggcaccac
catgcccgactagttttgtatttttagtagagacggggtttcaccatgtt
gaccaggctggtctcaaactcctgacctcaggtgatccacccacctcggc
ctctcaaagtgttgggattacaggcgtgagccaccacacccggccTAATA
ATTTATTAACTCATGAACAGTAGCCTTAAGAGAAAACGATTTAAGTTTTA
CTTTATATTGAAGAAGGCAGCATTTAAAAAAGCTCAATATTTTCCTTTCT
TTCCTTAATGCTTTTTAATTTCCGTTTTGTTCATTTTTCTAGGGCGACAT exon 5
ACCTTCTGTTGAATACCTTTTACAAAATGGAAGTGATCCAAATGTTAAAG BARD1_e05
ACCATGCTGGATGGACACCATTGGTAGTTGTCTGGTTTTTATTCTCATTC
TTTCTGTGTTTTACAGTTCTTATAGTTTATAGTTATGTAGTTGTCTATAT
ATCATCCTCTGCCACATATACTCTTTTTAGTCTGAAGAACTTATGTTTTC
ATCAAGTATGAGAACATGATTACTTTCCTTCTAGCTTTTCATTTGTGACA
GGCAAGAAATTGGTTACCTTTTGACAGACTACCTTTAGATTTAGGAATCC
ATTTGTACTGTACTGCAGAATTTAGCTAATGTCTAGAGGTAACAGCTACA
GCTGACATCAGGCTCCATTCTGTAGCACTGCATGTCACTGGAACCAAATT
TCTTGGAACAAAAAGAGGTCGGAGGAACTGAGTATAGGAAAGTGATCACA
AGGAAGTAATTCTCACTGAGGGTCTATCTTAGCCTCACTTATACCCTATC
CAATTGTAGATATATAAGGCAGTAGAAATCTTTGCTTACATTAAACATTT
TTAAAGGTCTTTGCTTATTATTACTAAAAAAGTGTGAAGCATAATCTGGA
AACAGAATGACACAAATACTTGGAAACAATTGGTATGTAGGCCAATTACA
TTAGGACAAACATGAGATTTGAATGCAGCCACAGAAGTGTCAGCAAATGA
AGTGGATGCCTTCATCCTGTAACTAGAAATTCAGCCCAGAAATTAGGAGA
CTCTCAGTAGGCTAGGTGCAAGAAAGCTGGGAGAGTTTAGTCTAGATCAG
TCGCTTTGCTGGTTGGACTCTACTGGCTGGCTAACCTTTTCAAAGAAAGG
TTAATTTTATTTTTTTCTTCAAAGCCACTAAGGCAAGTTGTAGTAAAGAT
TGGTTAGTGATTTAGAGGGTTTAGTGCCTAGTTTGGATTCCTTTCTCCTA
AAAATAGACCTACAGATGTTGAGTGTGATATCACTTATTATGAATTGAAA
TTGATGTACTTAAAAAGTATAGAATGCCAGAGATTTATTAGAATACAGCA
TTGTTTATAGTTCATTAGACATAAGGAAAGATTTGGATGTAAGGCATCTT
TGAACACTCCATTGTTGCCGTTTCTGTCATTCAGCAAGCTCTTTTTGTAT
TCCTACTCGTGTTATTTCTCTTATCAATAACAGCAGCCATACAGCAATTT
GTAGGTTCAGCAAATAGCTTATCTTAAAAGCATTCCTTCACGGATACTTA
CTTTGTTGCATGATATGTGTATGTACTGGTACAGATTTTATGTATCATCT
TGTTATTAAATATGTAGACTTTTTTCTTAATGTGTTACATTTATTGTAGA
ACATTTAAGGAGCTACCGTTTGGTTTAAAACTACATTTTCTTCTAAAAAAC
AGAAAAGTGCTTGACCCAAGGCTCAAATGAGAATAGCCTTTCTTTTTTTA
TGAGTTACACAGATCTTGATTGAAAGATTATTAATAGTAACTTTCACTCT
GTCAGCAACTTATAGTGTTTTTGAGTATTTAGGTAACAATAAATTTACTG
CCTGACGTTTACATTTATTTTTCTAAAGTGTGATATTATAATATCATCCA
TTGCTCTTTCTTATCACTTCTTTCACTTCTTTTTCAAAAAATTTAATTAG
CATGAAGCTTGCAATCATGGGCACCTGAAGGTAGTGGAATTATTGCTCCA exon 6
GCATAAGGCATTGGTGAACACCACCGGGTATCAAAATGACTCACCACTTC BARD1_e06
ACGATGCAGCCAAGAATGGGCATGTGGATATAGTCAAGCTGTTACTTTCC
TATGGAGCCTCCAGAAATGCTGTGTAAGTAGTTCAACGTAAAAATTATTT
TTAAAATGGACCTATATTCTTGAATCAAGGTGTGTGATAAAGCAGACTTT

AAAATAGTCAAGTTGATGGCTTTCTTCACTTTCACAACTAAAATTAGATG
TGATCATCACATTCTGCACTCATAATCAGCCTTCATGCCCTTTTTATGAT
Acagttggtccttcatattcttgggttctacacttgaggattcagccaac
tgcagatcaaaaataattgggaaatatcaatgacagatcggataaaaaaa
atgtgttacatatataccatggaatactatgcaactacaaaaaagaatga
gatcatgtttttttgtgggcacatgatggagctggaggccattatcctta
gtaaactaacgcatgaacagaaaaccaaataccgcatgttctcacttata
agtgagagctaaatgatgagaattcatgaacacaaagaagggaacaacag
acaccagagtctacttgagtgtggaggatgggaggagggagaggagcaga
aaaagtaactattaggtactaggctcaatacctgggtggtgaaataatct
gtacaacacacccccgttacacaagtttacctatataacaaaccttcaca
acttaaataaaaacctagaataaaagtttaaaaagggggaaaaaaaataac
actacgataataagtaatataggtaaaacaatatagtataaatatttata
cagcatttcatactattaggtattacaagtaatctggagatgatttaaag
tatacgggaggatgtgtgtagtttacaagtaaatactatgccatcttta
taagaaacttgagcagcggcacattttgacatcacgggttgaggaaccaa
ttccccatggatagcaatggggataactgTGCTGACATATTTGGGGGAGA
TTTACTTTCTTAATTCAGAAACAGTTGTCAATTTTGGAAGCTTTCATTTA
ATGGAAAAATTTACTTAGTGTTTATATTCTGTAGATTGATTTACACTTTA
ATAAGCAGTTATTGTAGAAATAATTATTTTGTATGCTTCCTAATAGTTTG
GAATTATTTTAAATATTTCATTCCCATTTTATTTTTTCTGTGTATTTACT
ATTACAACAGGTAAAGCATATAAGAATTTAAATATTGGGCCGGGGCAGTT
GAATTATTTCTTAAATTTTTTACATCATTCACATGAGGTTCTGGGTGAAC
TTTTTGCAGTGTCAGGACACAGAGATGCATTAGTGAATGCCAGCAGCTGG
ATTTTAAAATTGGCATTGTCAAGAGTGTCACAAGCAAATGCTGTATGTAG
ATTTTGTATCTTTAATCTGTAACTTGGTTCATGTCTCCCTATGTAGTATT
TTATTCTTTTCAGTTAATTAAGTAGTTGAAATGAAAATGCAGGTATTGCG
TCTCTGCCCTTGTTGCTTCTATAGGTTTGAAAAGGGCACAAAAAAATTAA
GTTTTCAATGGTAACAGATGCAAATAAGAATTATAAAATGCAGGGCTTCT
AGGTCGTAATTTTTTATTGGTAATTATTTAACATAATTGAAAACCAAATT
AATAATGCAAGAATGTACCATACCTTGGTGCTGTTAGTTTCAGTAAATGT
GTCTAATCTCTTTTATCTCTCATCACAGGTTATAAGATAATTGGGGCAAA
TTCAGGGGATGTGTATATTCTTCCGGTTTTTATTtgtgtgtgtgtgtgtg
tgtacacacgtgcgtgtttgtgtgtgtCTCCATATACATGGACCTATTTC
TTTGTAAGGACCTAACCGATATGACCCTCCCATATGTGTTGCAAGCTATT
TGGTAACGCAAATTATTGCTTGGGTCAGGTGAAACTGACTAGTTTTCATT
CTAGGGTTCTTAAATCAGCCATGACTACTTtaatggatggagcactggtc
agggcgttagggaatctagcttttatttgtgactccattactgactgtat
agcttcaggcaggttaatttctgtgccttggtgtcctcacctataaaaca
tgggtgatgcctgctgcatttctatttcttcagagcatagtgaggattaa
tgagCCAAAACACAGAGCCTGTCTTGTGGTTTGTACACCATGATTCGTCA
CTGCACTGTGAGGGCTTATTAATATACAAGGAGTGGCATGAGACAGTGAG
TGAGTCTGCCTTCACAGGTGCCTTCAGTGTTGGTTTAAGTAGGACTACCT
AGTGAAGTGGCAAATTTAGGTATATCTGTTCCTcagcactgtccagtaga
cctttctgtggtggtggaaatgtttatatctatgctggacattcagtagc
cactagtcacctttactattgaacacttgaaaattagctattgctaccaa
ggaataaattttaaaatgtattttattttaaccatatgtgccagtgacta
ctgtgttggacagcTGGTTGATACAGCTGGGCAGTTGGGAAGTAGGGTTT
ATTTCACCCAAGAGGTTGGGATTGATATTGGATAAAGGATAGTAGGGCAG
ATGAAGGGACAGATTTAATCCTAGGGTACAAGAGTATGGTGGCTGTTTCT
AAAAATTTCCTTTCCAGACTATTGGTCTTCATACTTTTTTCCTGTGTTTT
TTGCCTCTTAAAAACATGTCGTAGTcattgttgttcatctgattggcttt
acaaatttcctgactgttctcctccttttcaggttactttttctttattca
ctctccatcctgcagccagagcacctgcagaagaacacgagtctggccaa
gtcatttatttgcttaaaatccttccgtggcttcctcttaatgtttttaa
aataaaggttaaatcccaaaatatggtttataagaccctggatggtctgg
tgctactttcctctctgttctcatcggtcttcacagcttctcctttcccc
ctttccttcaagcatttttttgcaccagcttttctgaattttttgtttcctt
aaaatatgtagggtcttttacctcctagcctttgtacatgtctttgcatt
gtctagaatattTGTTTTTGGTAGCCAGTTTCTCATTTGGGTTTCACCTG
GGTTATCCTGTCCTTTTGGCCACCTTTCCTGATGTGGTACTTCTCCCCAG
TCGTAGGACTTACTGTTTCCTCCCTTCTCGTGAGGCTGTAAGCTGTATGG
GCCAGGGATCATGTCTGCCCTCCCTAGTTCTGCCAAGTGCCTAATGTAGT
CTGTATCCACATTTGTTTGTAGAGTGAATTAGTGTTTGAATGAATGAAGA

TTATGGTATACTTTTTCCTCTTTTTTGATAAAAGGTCAGATGACATGAAA
ATGGGAATTGCCTCCTTCTGCCACTACCTTTACTGTGTGACAAGATGTCT
AAAACCAAGTTTCCCATATCTGTGCCTTTTCTAAGGTGGTTGAACTGGTT
CCTGGCAGCTTTTGCCCAATTGGCATCCATGGCATGTTACTTTTTGTGGG
ATTGTGGCTACCCAAAGTAATTCCTATAGCCTTGACTTTTAAGTGCATGC
TGTCTCAATGTTCTAGAGACTGTCTTCTAGAATTAGGTAGTAACAGGTGA
GAATAGATGAAATTTATTCCTGCCCTTTCCTCCATTTCTTAAGGAGATCA
TGGGAAGGGGGTCTGGAGCATGGAGACTATGGGATTCTTACTACTCTTCT
ATTTCTTCCTGAAGCTCTTACTCCTTATAAACCATTAACATCTCAAATTC
TGGAATCTGAGTTGGCAAACAAACTTTTTCTGTCTCTGCTTACTGTATCT
CTTACTCTTGCTGGAACTCTTTTCTCAATAACCAGGCCTGAACTCTTCCC
ATGGAAGAAGGGTAAGAACCAGCAAGGGCAAACACCATTCTGTCTGACCC
TCTTTAGGGTTTGCTACTTTGGCTGATTGAAGAAGTAAAGTCCTTTTTGC
TTACTGTAAATTTCTAAGTGGCCTCCTTTAGCTCTAGCATAGACATTTTG
GCTGTGTTCTTCATGACCTCAAGGTTGAAGTTTCTCTTAGATGCCTTCC
TTTCTTCTTAAGTCTTCTCATAGTCCCTCCATTGCTCCTTTGCCTCATCA
TCCCCTCAGTTGTCTGCCTGCTTCTGTTCTAGTAGGAGTGAACAGTGAAC
AGTCTGTTAAAGCACTGATTATAGAATGGTGTGAGGTATGCCTTCATAGC
ACGGTGCTCACAGTAGTATGAAAGTCTCAGCTAGGGCTGAAAGTAAGACC
AGAGGAAGTCACAGAAGGCTTTCTGGAAGAAGAAGAACTTCGAGTTTTGA
AAAAATAGAACGGGAGGATGGGATTTTGAGAAAGATAAGATATGGGTGCT
AAGGTACTCAAGGCCAGACTGCTTCTTCATTCATTCTAGGAACTTCCGTT
AGTGATGTAAGTGGAATAAAAGGATGGAGGAGGATGAGAGGCCAGAGAAG
ATGGGCAGGGTCTTTGAAAGGCTGTGTGTGCTAAACAGAGTGCTGATTTT
AAGTTGAAAACCTCCTCCTTCACACTGGGGagggaactcagttaagaggt
ttgtccagtaatccaggaaaggagtgatgagagtcaaatcaaagtagtga
cagtagtggtgggagggggtggggtGGAGAATTAGCCATATTTATTAAT
TTATTCTTTCAAATGAAGTTTGATTTACTTTGTACTGTTTCAAAAATACC
ATATTAGGATGTTTTGAAGTTGGGTACGTACATAGATTCTAGTGGAGTAC
CGATTAGGATGTTGATTGAAATTGGTAAGTATATACAGTGGATTAATAAA
GTTGGTTTCTATCAAGTCGATTGATAAACTTTATGTCGTGTGTTATTCTT
TAAATGAATACAtcagtttgctaggttttgtttaggaatttgcatttt
aaaagttagagtgatttgtgattttatctttcacttcttgtcggtagatt
ttgatatcaaagttagactcataGCTTCTAAAAAGTAATCAGGACCAAGC
TGCATGTCTCCATTTGTTTAAGCATTCTGTTATATATATCTTAGTAAAGT
TTTTAGTCTGTTTCATATATATGTATGTAGGTATACACATGCATATACCT
ATTAATTGAGGTTATTCTAGTTATagactgagtatcccttgcccaaaatg
cttgggatcagaagtatttgggatttctttcagattttggaatatttgca
tatacgtaatgagatgtcttagggatggttctcaagactaaatgtgaaat
ttatctgtgtttcataaacatttatacacatagcctgaaggtaattta
tacaatattttaaacaattttatgcattaaaatggtttttaagtactgt
gtttggaattttccgcttgtgtcatattggtgctcaaaaagtttccaatt
ttggagcgttgcaaatttcagatttttggattagcaatgctcaacctgTA
TAGTATTTGATTATTTGTTGCTAGTAAACTGGATATTGTGTTAATAAATA
TAATTTGTTATTCTTGATATATAGGAGAGCTGTTGATTTAGTACGTTTCT
ATCCAGTCAACACCTAATTCTAAAGatctaattatcatttattaacttct
atttcttattgcatttgtcagtgcttccagaatgatgcaataatgatgat
agttggcttgcttgttttgttcttcctttttaaaaaagtactgacccat
agagttatagtcaatactaatatccatattaaatacaaagattaatgttc
tttattgccctgtggaagctttcttccctttattattttctaacaagact
gtatttatttattgaatgattttcagcttctgcagagatgatcTTATA
Cttttttgtttttgtttttgagatagggtctcactgtgttgcccaagct
ggtcttgaactccagggctcaagcgatcctcccacctcagcctcccaaag
CTTATGCTTTTTTGActattgatgttgttaatacatttaaaaatattaaa
taatgcttgtattcctgggataaccttgacttggtgttgtgcatcattta
aacacaaattttacatgaatttgctagggttttaggaattttgcatttat
gattataagttagagtgatttgtgattttttatctttcatttttttgcctc
ggattttgataccaaaattatactcatagtttctaaaaagtaatcagaat
gctttttttttttttctTTTTACCTTTCCTCTTCTCTGGAACATGAATAG
AATGTCTTCTGGAAATATCCTGCCCCCTGCCAACTTAGCATAACAGTATC
TATTTTGATAACTCACATATTATATAATTTTTGGAGATCTCTCTAGTTTT
GTTATTAGTAGTAATCACTCTGATAGACCAGTGTATTCATAATAAAATAC
AGACACTAAAGAGAAAATGGACTTTTTTTTTTTATCCCATCAAGTTCATC
CTTAAAAAGTCCCTGTGGAttgagaagccaaggcgggtggatcccgaggt

caggagatcgagaccattctggctaacacggtgaaaccccgtctctacta
aaaatacaaaaaattagctgggcgtggtggtgggcgcctgtagtcccagc
tactcgggaggctgaggcaggagaatggtgtgaacccgggaggcggaggt
tgcagtgagccgagaccgcaccactgcactccagcctgggcgacagagcg
agactccatctaaaaaaaaaaaaaaagaaaaaaGTCCCTGTGAACTGTAAA
ACTTTTACCTATTTTGACGTTTTATCTGAGGCTGTGATTTTGATTAACTT
TATAACTTTCCTGTTACTGTAAGAATATTAGTTTGAATTAGAAGAGTAAA
TATTTTTGTAATGCAGCCCTAGGAGAGAGAAGAAAAACTAAGACAAAATC
CAGCCATAGAGTTAGTCTCGGATGTCACTGGGAGAGAGGAAGGCTTGTAT
AAGAGGGCTAAGTAATCCAGGTTTTATCCTGATACATGTTCTGGCATTAT
TAATAAGTTATGggcctggcgcagtgcctcatgcctgtaatcccaacact
ttgggaggccgaggcgggtggatcacttgaggtcaggagttcgagactag
cctagccaacatggtgaaaccccatctctactaaaaatacaaaaattagc
cgggtgtggcggcgtgtgcctgtagtcccagctactcgggaggctgaggc
acaagaatcgcttgaacctgggaagcggaggttgcagtaagccgagatcg
tgccaccaccctccaacctgggggacagagcgagactcggtctcaaaTAA
TAATAATAATGTATCATGAATGGTGCTAAGTAAGATGTGACTTTAAATAT
TGGAGACCAGGCTTAAGAAGTACCTTTTCAGTAGTGACATATAAAATATA
AAGATAACTGAGAGCTTTTTTTGTAGCCCAGGAAGTGGGGACAGTAACCA
ACTCAGTCATATAAGTTAGACATTGAATGTGTGTGGAAGACTAGACATAG
GAAAAAGAATGGAGTATTATCTGCTGCAAAGCCAAGTGAATTTAAAAGCG
TCCTATTGAATATTGGTGGAGGGAAATCTTGAAACCTTATAAGGCAAGGC
AAAATAGCTAAGAATTAACCATATTGTGGTATGAATTTAAAGTCAATGAT
ACTCTTTATTTGCCATCTTCACTATTTAATTTTAAATTAAATGAGCTTTT
ATAGTTCACCAGTGAAGTCTCCCAATCCTATTTTTTCCCATAGAGGCTGT
GTTCTTTATTAATTTTCCTACTGCAATGTTTTTGGGCCCTAAATCTTTAT
AATTATGGAGGATTCGAATGTACAGTGGAAAGTCATATATCTACTTCATA
CTTTCTCTTTTGTTTTGATTTAACTCTAATATTCTGGCAACTTACTGTTT
TTATATGTCTTGCATTTAAATAACAGTATTAAGTCATTTAACCCTTAATT
TTTTTTTGCATAAAAAAAGTACCTTTTGTGAGCTAAAACAATAAGAAAGT
CAGTTCTGTTTTAACCCTAGATCATGAAAAAAAGATTTATAATCTTTGTT
GTCTGGGAGTATTTATAACTGTTCAAAACATCATTGGTAATCTGTCTTCC
CCACTGCCCTCTGTACATCCCAGTGTGAAGCTAGAACCACAGCTCTTACG
GTCACATGAGAACCATTTTGAGTGTGTGGCGAATAACATGTGCTTCTTTT
TTTTTATTTTTAATGGATATGTAGGATTTTAGAATGTGGTACTTGATCTG
CCATTAACAGTTTTCCTTGGAAGGATGTTGAATTCTGAAGTAGAAAATAT
TACATGGACCATTGGACCTATTAATTGTCCCACTGCCCTAGGTTAATTTT
AAAAAATTAATTGCCAGAGATAATTTATGTTTTATAGCTTTAAAAACCCA
TGTTGCTAGTTTGAATTACTCTGCTTTATCCTGCTCCATAGCAAGCAAAG
TGACTTGAAAAATTAAATCTATATGTTTGCAGTTTTGTAGTTATTCATGA
AGGGTTTCATTCATGAACTCTCTTGGTATCCTGTCATGCGTGATAGATTT
AGTATTAGTAAAAATGCCTTTTTGCATATGTTTTTGCAGTTTGGATGGTC
TGTACATGCAAGTACAAGTTTAAATTTGCCCATCTCTCCTTTCTCTCTTT
TATTAGTGGTGTAATGAATTCAGATCACCATTGTAGGCAGGTGTCTGCCA
TATTATTACTGAGGGGAAAAGGATATTAAAAGGTTATACTGCATCCTGAA
TGTGGCCTTTGGGACAAAACTGAAACATTGAACACCACAGTTTAGCCCTT
TCATAAGTACTTCCTTTTCCTCATCTTCAGGATCAGAAGGATTCCTAGTT
AGGAAAAGGATTTTCAGAGTTATGTGGTTCATACTCTTTAGATAGTGCCA
CATGGGAAAATGAAAATCTACTCTTAGAATGTTGTTAAGAAGACATAATT
CTATACTTTCCCTTAGTAAACTTCCAGATTATCATGAAAAACATGCATCA
ATTGTATCATAAAATTGGGGAAAATGAAGATATGAGGTTAATGCTCCAGA
CATAACTTATTTTGTGCCCTTATCTTCATTCTGTATCTGTCTTCCAAAAA
TGCAAATCCAATACAGTAATATAATGGcagttattgagctgttcatatat
gctaggtactgttttaaatgcttttgatgtattcatttgattctggcaac
aatcctgtaaaatagatagtgttattatttccatcttataaatgaggaaa
caggcacgggatttaagtaattcgtgtaaagtcataagacttttgagtgg
cagggctgaaatgtaagttcaggcagtcttattcggtaaccccaactctt
aactaccatgctacgttgATGCCATCCCTCCTGCTTACAATGATTTAGGA
AGCTGTTCAGGGTTTCTTATTGCCTTTGGGAGGAAGTCCAACTCTTTAAC
ACCTCCTCTTTGTTCTTATATAATCTGACCATAGCTTACAGTTCGAGAGT
ACCTCATATTCCTGTGGTACATTCCCTCTCTTTCTCTCACTCTCCCTTTA
CCCCCTTTTTTCCTTTTTTTTGGTGGTTCTAAAAAGTTCTAAGTGCTATC
TCACTTAAAGTCCTTCACACACTTTTCTGTCTGCTAGGATATTCTTATTC
TGTTTGTGTACCCGCTTTTCTTCCTTACCCTTTCAGGTCTGGGGAGCTTT

ACCAGACTGCAAAACCTTTTCTGTGTGCCTCTCTGCTTTCCTCCTCTGAG
TTCTGTGAGGACATTGTTTCTATAGTGCCAAAAGTTGTTGGTACATTTTT
TTCAAAGTATTTGCTGAATGATGACTATTTTCCAAACTAATTGCAAAACA
GAGTTTGTGACTTTTCCCCCTACTTGTTTATACAAATATATTCATTCCAT
GTGTGTTAAATAAATAAACGATTATTGCTACTTCTGATATATGCAATATA
TATATCATGCATTATGTATATTAATACATATATCCATTTTCACATAGGTT
CTATCAACATCAGTAACAAATCATCCAGTATTTCATACTACATTTTAGAT
AGCAAAAAGCAATGACACATGAACACCTGGATTGTCTGTTCAAGATTTTA
AAATGTAAAATCTCCAGTCATATCTTTAAAGATTTTATAAAGCATATTTT
CCACATTATGATTTAAAATCATAGGTAAAGGAATAGCGCAAGAGTACATT
AGAAAAAAAAAAAACTGGTGAAATCTGCCAGTGTACTGTGTTGTCAAGGA
ACAGAAAAAAAATTAGAGCCTTTGAATTTTTAGGATTCAGTATAtgtatt
agtccctttttcatactgctgtgaaacacctgagactgggtaatttataag
gaaaagaggtttaattggctcatggctccccaggctatacaggaagggcc
tcaggaaacttacagtcatggtgggagatgaaggggaagcaggcacatct
tacgtggtcagagcaggaggaagaggcgaggaggtgctgcagccttttaa
TTTATTTATTTATTATAAttttttttatttccataggtttttgggaacaag
tggtatttggttacacaagtaagtttttttagtggtgatttgtgagattgt
ggtgtacccatcacccgagcagtacacactgaacccagtttgtagtcttt
tatccttcaccaccttcccaccttttccccttgagtccgtgaagtctgtt
gtgtcattcttatgcctttgcatccccatagcttagcttccacttatgaa
tgagaacatacaatgtttgattttccctcctgagttacgtcacctagaat
aatagtctccagtcccatccaggttgctgtgaatgccattagttcattca
tttttatggctgagtggtattctattgtatatatacaccacagtttcttt
atccactcattgattgatgggcatttgggttggttccacatttttgcagt
tgcaaattgtgctgctataaacatgcatgtgcatgtatctttttcatatc
atgacttcttttcctctgggtagacacccagtagtgggattgctggatca
aatggtagctctacttttagttctttaaggaatctccactgttttccata
gtggttgtactagtttacatttccaccaacagtgtagaagtgttccctgt
ttaccacatctatgccaacatctgttatttttttatttttttaattatgcc
cattcttgcaggagtaaggtggtatcaatatggttttgatttgcattttc
ctggtcattagtgctacaccctttttaaacaaccagatctggtgaggactc
aatcactatcacaagaacagcaaggaggaaatccgcccccatgatccagt
cacttcccaccaggccccacctccagcattgggaattataatttgacatg
agatttgggcggggacacaaatagaaaccttatcaGTATAGGTAAAGTAT
ATTATCTACATATAATCATTTTCAATTCACTGAATTCGCGACTGTCCGTT
GATAAGGCATTAAAAGAACTGACTGTCGAAAGTGGTACATGTAAAAGATA
TTAATATACTGTCGTGTACTAGATGTAAATTCAGCAGTTAAGATCTCGAC
ATTTTCATATATTCCAAAATAGTTTTAAATATTTTGGTGTTGCATTGACC
CATGTCATTTAGCTAGTAGACACATGTGCCTTTCCATTTTCACAGCAACA
TACGCTTTGTGTCTGTAATGTTCATTGCTTTATATCTGATTTTTTCATTG
GTTTTTCATACTGTGTTTGGTTTCTACACATGAGCACACAGTGCCTAGAA
GGTTTGATAGAAAGCTTTCTTCCATCAGTTGCATCCATTTCAGTAATCAT
GTTGCTTATTCCCTACACGGCCCCTTAACAGTTAACCACCATATTCCAGT
TTCTAGTCCTGGGATCAAGGCCATTTAAATCTTATCTTCTATTTCACCTC
ATTACTTGTTTGGGTTTATACTTTATCAGTTTGATACCTGTTTGTTGAAT
GAACGTTTATCCTCAGTAGGATTATAGGCAAGGACAGTGTTTTTCTAAGT
CTTATGTGTCCCTCCAAAACACCTAGTTTGGTAAAACTTTAGGttaaatc
atgctagaaaagagagaatttactatatttaagtgtatttagaaaaagcc
attttataagcttccccagtaacccattctaataAATATCAAATCAAGTT
ATTGATGTCTGTTTTGAGTCCATGATGTAGTAAATCTAAAAAAGAGTCTT
CTTCCCTTGTATAGTGATGAAGAATAATGTATTTATCACTGACTTCTCTA
TTTAGTAATAAGTCATGTTAATAGTTCCTGAGATACATTTCAACATAACT
TTTCCTGTATGTAGTACCTATTAGTTTTACAATGGGAATAAAATCCACCT
CTGTGTATTTTAAATTGATTTTTCACACTAATTCTCTAACTAAAGTGTTA
GTCTGTTAAAATACATCAGTGGGAAGAAAGCACACATTATTGTGCCCAAA
TAATAAGAAATCTTACACATGCCCCATCTTCTCTTGCTATGGTCTTTGAA
CGTCCCACATGTAAACTTACATCTACTTCCAAATTTTAATGGTTCATGAC
AAAGCAGAAGCAACATGACTGGATTTAATAGGCTCTATTTCCCATGATGC
AGGACTGAGGTAAACTGATTATCTTTCCATCTGTAGCACTTGTAATGTGC
CATTCAGTTCATTAGCGTTACAGGAGGGCTAAGAGTTTAGTGAATTGTGT
GTGAGCAGAAGCCATCCATAATCCAAGCAGAGAAACCACATGTTTTAAGA
CCATATGACATAGTAAGCTTTGTATAGTAAGGATCCTAAATGTAAATTGG
AACTCTGGTAGTCACTTTGTAAAATCATCTTACTGGCTTAGACCATGTTT

TACTGGCTGATTTTTGGTTTATATTCCTCCCTAGCTGATGGTGGTACTTT
GAGAAGCACTTGAACAGTTTTTCATGTTGTCATCATGCACTTTTTTTCAA
ATATGTGTAAAATAACTATGCTAATGTGATTCAGTTGCTGTGCATTAAAG
TGCACTTGATTGGGCTCTTAGATTTTATTTTTTTGAGGTGTAAGGTCTAT
GTTTGTCTCTGTCAAAAAGTCCTAGTGTATGCATGGAtttttttttttct
catttttttctttttttgttgaaacagtatctcttgctcaggctggagtac
catggtgcaatcatggctATGCAGTTATGATTTTTTTTTTTGGATGGCAT
ATAATGTGGCCATTATATGTTGTTGTTTTGTTTATTCATTTTTGCAC
TTGGTGGTTTAAGACCTAGAACATTGGTTCTTCAACTCAGATTTCTATTT
TTTGATTGTCATATGTATAGCATATGTGGGTGAGGAGATTGTGTAGTTGG
TATATGAGCATAATAATTTAGAGAATGAATTAGGAAGTATCAAGAATAGT
GGATGAGTTTCTTTTAAAATACCCTGAGATTGTCTGCTGTAAATGTATAC
TGTGACATGTGTCACTTTGTTTTAAATTATAAAGCTGAAATGCAATCCGC
TGTATTATATCTTGGCTGGAGAAAGACTGTTGCCAGAGGAAGGTTGTTGG
ACTTGGACATAAAGAAGAGTCAGAATACAAACTTGTCAGTGTTTACCCAG
GACTGTCCAAAGAAGAAAGCAATTGGACCTGATAGTCACGAATTGCAGTC
ATTAGAGAAACATTTGCTGTGGTTCATTTcttattctaatacattagctt
tcttttcccactctgccatcaaaacagttttcaccaaggtcattagtgac
cttgtctgtgaactcagtagatgcttatatccctcatctacatgatctct
ctctgcagcatcaaacggaattgGCATTTTTCTACCTTTCACTTCCCCTA
ATGTTTCTCTCTGTTTCCTTCCAGATCTTTTTCTATACATTTGATACAAT
TTACAGAATTAGTATTATACCATGTTTATAACTTCATGTCCCATTTACTG
CATTGGATTTTCACATGGACTTCTGTGGCCATTATATGAATATAGCATAA
TCTTTAAAAAAAACATAGAAGACTATAAAAGAATTGCTCATTTTGAAAAC
TTCTTGCCATGGTATAGTGTATTTAAATCATATTCTGAAACCATATGTAA
GGAAACAGAATAGTAGACAGAAGTCCTTTAGTTTTAACAAGTTACTTATA
ATTGAAAGACTTTAGGCTGGGCAATATTTAATTTCAGTGAAATTGAACAA
GGAGAAtatgaccttagttaagccattggaacttgttcttctctcttttt
tttcttatcttgaacaggatcaaactgggatctctaaaatctcttccagc
gtaacattctgtgacttgtgaATTAGTACATGCTGAGAAGTTTGTATCTC
TCTGAGCCATAATACTTTGTTCTGCGATGGTTGTGATTATTATAGGTTAT
TGTATGCACACATGTTTAAATTAATTTTTAAAGTACCCTGTTAACTATAT
TATTAAACTGTTTGTTATGTGGCATAATTTTCCTTCTAGTAGAACAAAAT
CCCTGTCCTGTGAATTTATCTAATTTTTTTATTGGTTTATAAAGACTATAT
GGCCTATAATAGCTATAGTAAATGATTTTTATTGGCATTTGAAAGTCTGT
CACTTATAGTGATTGGTGATTATGAAGCCATATTTTAATATGAATAAGAA
TGCAGAATACAGTTGTGAAAAATTCATAATACTATATTCAGTAAAAACAA
TCCCTATAATCTGATGTCAAACTGAAATTTTACATCATTTCTCCTTTGAG
TTCAGCAGCTTTTGATTCTAGATTCTTCTGCCTAATATGAGTTCTGAGTA
ATTTATTTTAGTTAAAATTGTATATTATTAAGGATGTTGAAAAATTGAGT
CGAGTCACACATTTGACTTACTTAAACACATCTGCACTTATTTTACCAGT
AATATATTTGGTCTGCGGCCTGTCGATTATACAGATGATGAAAGTATGAA exon 7
ATCGCTATTGCTGCTACCAGAGAAGAATGAATCATCCTCAGCTAGCCACT BARD1_e07
GCTCAGTAGTAAGTATGGATTTAGCTTTGGGACatttatatattttatta
aaattGGTTATGAAAGGAACATAATAGAAAAATTTCCATTTGACCAATTG
CTTAcattcaccaaacaattattgagcacttcctgagtattagctactgt
gGATTCAAAGACATAATCACAGTACGACCATCTAGaaatacttattgagc
ccactctgtattttaggcagcattcataaaacaatgaatatgactggtag
aactcttattctcagggaggagcttaccatctggtgagaggtaggaaaga
gacaaactgtaaatattgaactaatataaataaaataatttcagacactt
agacatgagtgtttcgaagatgttgtagagtgtcgttgggtggaggtagt
gggcttctgcaaggccattgctttagctagggtcacagagtggagcctta
aagcactgatttgaattgaagcctgaatgttgaagtgaggaggctgccag
gtgactatctggaggacacagtgtataggcccttcagtgaatgaggctgg
tatgttggaaggttagaaagaagtttgttattgctggagactagcgagcA
TCAAaacgggactttcggatagattacatatgaagtgtgggaaaagaatg
gaataagtatgaccttagcagggaaatagatggtaatgccatttgttgcc
atggagaaggaaatcaatagttctatgtgccaatttgatgtctgtaaaac
atgaaatggagatatcgaatagataattggatatgaaagtctggagctta
ggagagaggtacaagctagagataAAGTGGAAAGAATGCCTGTTTCTTAG
GAACCCATAGCTTAGCAGGGATTTCAGATATAAAAACTTTTACTGTACAT
TGTAATAAATTTGTAAAATAAGCTATAAAAATATACAGTAGAATCAAAGT
AAGTAGAAACTACAGGAGATGTCAGGAAAAATTTCACAGAAAggccaggt
gcagtggatcgcacctgtaatcccagcactttgggaggctggggcgggtg

gatcacttgaggtcaggagtctgagacctgcttggtcaacatggtgaaac
cccatcccatctctatgaaaaatacaaaaattagctgagtgtggcggtat
gcgtctgtagtcccagctactagggagactgaggcacgataattgattga
acccgggaagtaaaggttgcagtgagccgagatcaggccactgcattcca
tcctgggtaacagaacaagactgtctcaaaaaaaaaaaaaaaaaaaaaaa
aaatatatatatatatatatatatatatatatatatatacacacacacac
acacacacacacacacacacacacaGAATTTCACAGAGAAAGGTGTCCTA
AGTCTTTCAGGGTAAACATTTTAGCAGAgtgtctaatagaatgctgttgg
ctgccagtaacagaaaacctttctcaaatgcttgaagacaatttattac
tttatatagaattaatcccaatgcaggatagctctgcatttgcttaattt
agcacttagtggttgcatcaaggaaccaaattctttgtatctctttacct
tgccatcctcagtttgggggatttgtcctcactaagaagtttcctttaat
ggttgtcgaatgattgccacaattctagatatgacagtggccaactgaaa
aagagacactaaagtctttccctgaagcctttccgtggacttcccctgtg
tcttattgtcagaaattgattttgacagcccctcctggggctggaggtta
gggtcagtctttgaaagggggctacctgaacaaaactgggcttctgtta
gtgtggaggaagtgagcaatagattttgagtgtaacagccaacATTCCCC
ACTGCAGTCAGGTGTACAAAGTGAGAATGGCAGCCCAAATCTAAGGAACA
GCTGCTCAGAGACAAGGAGGCATAAAGGACCTTAGGAATTTGAGAAACTA
TAGCATTTACTGTGAGAGGAGTATGACGTGTGTTTAAGAGGGATTCAGGG
TAAAGGGTTAGGTTGGGACCAGATTGTGAAAAGCCTTAAATTCCCTGATA
CGAGTTTAGACTTAATAGGGTACCTTCAGTGTGTTTATAACTGAAATAGG
ACTGTCTGCAGGCTAGCTGGGAGTAGGAAGAGACTGAAGGCTGATTGGGA
GGCTGCAGTAGTGGAGACCAGGTTTGAAATGCTGAGGACTGGAATTATAG
ATGGTAGTTATGTGGGGATGGAGAGAAGAAGAAGAGTTTTAGAGAGAGTT
GGTAGACTCACCAGATCCCAGTACTGCTCAGATGGGGGAAAGTAACAGAA
AGGGAGGAGCTGAGGTCAAGGATCATTCACAAGAGTAGTAGTAGATGCCA
TTGTTTAAGATAAGGAGGTAGCCTAAGGTAGCTACAGTTTGATACTCTCA
TAGATGCTTTCATGAGCTAGTTGCTTTCATATCTTGAAAACCCTATGTCT
TCTGCTTTAGACTTCAGCCTTATAAATCTGTTCCAAACTATAGTTTTGGT
AATGAATTGGAAGAGAAGCtagaaatcagcagtttaattttctcacttta
caaatgagaaaagtgagacctaaagaggctatgatttgccccagggttaa
ccagccattaaAAAAAAAAAAAGGTTGTTTTTTTCTTTATTAATGGACCA
GTTAAAACTCACATTTCTTAAAACTTAGGTCGGTACTCAATTCAGTCCCA
CCGTACTCAGCAACTATAAATTGACTTGTTTTCTTCATTTGTACTTGAAG
TCTGACAGAGCATAGCATAACCAAGTTGTCCTTTGCTGATGTTCCTTTTA
TGTAAAAGCTGGAAAATGATTACTTTAAATGTGTCAAGAATAAAGATATC
TTTATGGTATTCTTGGAGTCTGGGGGTGGCAGTGCCTTTCAGAAAAGGAG
GGTTTTTATTACATGGAAGAGACCTATACTTTGGGCATGTCATGTAGACA
TGGCCTTTTGAGTTTTACTATTTACTGTCTTAATGGTTGGTACTTGGCTA
AGGAAAAGATAATATCAAGTCCTTCAGGTTTAAAGGGTAGTTTCTTAATG
TGCAGTCCTTAAAGATGGAACCTCTGGAATATACCTTCccccagccacta
ccagtctcttcttcactcactctttttcagtcacatgggctgccttactg
tgtcttgagtccaccaaacatactctcatttcaggtgtgcagcaccactt
gtttagtttttttttttttccaggaactagatgttcccatggccagct
tcctcacacccttcagacccagctctagcatcgtcttagcagagagggct
ctgtgccactgcacagtaaatagcaacctcccagtgctgtctaaccccct
tcccctggtgtgttgtaccatactatttctcatttgatgtaatgtatttt
tattttcttgtgttgttttcccctctcaactagaatataaactgtgtgag
ggcaggggctggctcttctgtttactatagtagccctagcattgagaaca
gtgtttggcacatactgagtgttcagtgtgtgctgaatgcatgaaGGAAC
ATTAGAGTATTCTTTATGATACATACTTGCCTCTGAAACAAGCACAAGTG
CTTGGTAGCGTCATTGGGAACCCTTTAAATCAGTGATCTCCAGTGAGCTG
CTTTAGGGAAGCAGCTCACTGCTTCCCTAAAGGAAGGGGTTTGAATTTCT
CTCCTTTCTCTTTAGAATTTTTATGTTTTAAGTAAAATTTAGTGGCTGTT
GGAAATCTGCTTTGAGAAGCAACCTCTAATCTTTTAAAAATTTAAAAAAC
TCCTTATCATTCATTCATTTTGAGACTTCTCTGTTTGTGATTCATTTTAA
GCAGCATGTTTACCTTTTTAATTTTTTTTCTAGATTTTTTAACCCCATTT
TTGATGAACTTAAGAAAAACTAATGATTTTATCTAAGAAAAAGTACCCTA
TTTAATTATTCCTTTGAATTTAATGCTAATGAACATAGTATCTAATCTCT
ATAAAATCAGGGTATTAAACCTTTGGGGTGTTTTTTGTACCTCTTTTGAG
CCTCATGGGTCAGAGACCTATGCTTATTCAAGTCAAATCCCCAGCCCCCA
TGCCACCTCACCCCAAATCAGACATGTGGCTTTGGCAGCTGTTTGCTTTA
TGTTTTATTATAGCACTCTTTAACTGTGAGAAATTAGTGCCAGTTTCTAT

TTTTTATGCAAATTTTTAAAAGTATGATAGGAAAATACTTGACCCACTTT
GGTGCTTTCCTTGGGTTGGTAGAAATAGTATCATATGCTATATTTAGAAA
TGATATCTTTTTCACTCTTGTGTTTTGTGCAGAGAACTAATGTAGCTGAA
GTGGAGTTATATAAATTGAGAAGTAATGTGTTCTGTTTAGAGCTATTTTT
ATGGTATTTGTGTAACTTAAGAATTTTTGAAATACTGTAGTGTTTTTAAA
TATTATCAAAGAGTAATTGAACAATCCATAAAGTACACTGAAtattttcc
ttttttaatttttttatttttatttttattttattttattttattattat
tatactttaagttttagggtacatgtgcacaacgtgcaggtttgttacat
atgtatacatgtgtcatgttggtgtgctgcacccattaactcgtcattta
gcattaggtatatctcctaatgctatccctccccctcccccccaccccac
aacagtccccagagtgtgatgttccccttcctgtgtccatgtgttctcat
tgttcaattcccacctatgagtgagaacatgcggtgtttggtttttttatc
cttgcaatagtttgctgagaatgatggtttccagtttcatccatgtccct
acaaaggacatgaactcatcctttttttatggctgcatagtattccatggt
atatatgtgccacattttcttaatccagtctatcgttgttggacatttgg
gttggttccaagtctttgctattgtgaatagtgccgcagtaaacatacgt
gtgcatgtgtctttatagcagcacgatttataatcctttgggtatatacc
cagtaatgggatggctgggtcaaatggtatttccagttctagatccctga
ggaatcgccacactgacttccacaaaggttgaactagtttacagtcccac
taacagtgtaaaagtgttcctatttctccacatcctctccaacacctgtt
gtttcctgactttttaatgatagccattctaactagtgtgagatggtatc
tcactgtggttttgatttgcatttctctgatggccagtgatgatgagcat
tttttcatgtgtttttggctgcataaatgtcttcttttgagaagtgtct
gttcatatccttcgcccacttgttgatggggttgtttgtttttttcttgt
aaatttgtttgagttcattgtagatcctggatattagccctttgtcagat
gagtaggttgcaaaaattttctcccattctgtaggttgcctgttcactct
gatggtagtttcttttgctgtgcagaagctctttagtttaatgagatccc
atttgtcaattttggcttttgttgccattgctttggtgttttagacatg
aagtccttgcccatgcctatgtcctgaatggtattgcctacgtttcttc
tagggtttttaatggttttaggtctaTGTGAGGAGGGTTTATAGCGTTTA
GGTTAGATGTGGTTACATCATCTTACAATTTAGTGACTAGGAAAGATGAG
CTGAACTTTTAAGTGCTGAATTCTGTCATTTACAAACACTTTTATGGAAA
TATATATATATATGGACATATATTGTTTCCTGGAGTCTTTCACTTCTCAG
CTTTAATGCTGTCCACTCTTACCTTCTCTTTATTTTTGTCTTTTTTGTCT
TCTGTGAATTCTAAGTCTAGATTTTAAAGTGAAACTATTAGTCTTTGTTA
CAGCACTTCTTTCTTAGTCGTGAGCTATATGGACTTCTTACTATTTATGG
ATCTGATCTCCTAAGTTTTGAATTAACTTGTCTGTTTTTATCTTTTCCTA
GTTTTGAGGGGTTACTATTTTGATGCTAATTTGTTTTCTATCTTTGAGGT
CAGCACTGTTCTAGAAGCCTTGGCATTCTTTGATTTTTCAGATAATCTCA
GTTTAAACTAAACAAGTTTGATTTTAACTCTATTGGGACAAGTTAGTGGA
GGTGGAATAGGGAATTGCTGATTTTAAGTGGATATTTTAAGTTACTTGGG
AAAAGAAAAAGACTTACTGGTGACTGAATGAAGTAAAACCCTAGAGAGAC
CCAATTTAAAATTGAAGAAATGAGATGCCCTGGGTATAGAGAGCTATCAC
AATTGACATTTTCTTGAGGGAAAAATAAAGAGAAAAAATTTATTTAAAAG
*GTTCTGGGTGTAGATTCAATG*GAAATAATTGAAAATTATTAGAGTAAACT
AAGTAATGAAATTCAAGCTTATATCAAGTAACAGTCTGTTTAATGTCTTT
GTCTAGTCGTCTAATGTTTTTAACACTGGTATCTCCTTTTATATTAACAG
ATGAACACTGGGCAGCGTAGGGATGGACCTCTTGTACTTATAGGCAGTGG exon 8
GCTGTCTTCAGAACAACAGAAAATGCTCAGTGAGCTTGCAGTAATTCTTA BARD1_e08
AGGCTAAAAAATATACTGAGTTTGACAGTACAGGTGAGGATTTTGAATTT
TGGGAGGTGGGGTAGAAAAAATGTTAAATAGATGATCCTTTTGGAGAACT
ACCTTTGATAATTTACATATGTTTTAACCA*TTGGGAGATGGCTGTATACT*
TTGCATCTTGTAATACATCTAAATTTTTTTCAGTAATAAACTACTTATA
GACAACAACGTAGTTAGGAAATGTAAAGTTTAAAGGTTTGCATATATTTT
AGGACAAGACAGTATTTCAGAAGAGCAAATTTGTGACTTTTGCTAGTGAC
AAATAAATTGTTGTGCAGTGTAATGTCATAATCCCTTAGCTGTAATTTGA
AAATAAAAATTTGTGTTTGCCAATATGGCTTTAAGATATATGGTTTATGA
TCTGATTTTTCATATTGATGGCCAGGTTAGAGAACTAGATACTAAATAGA
AGTAGTCTTACACTTAAGTGTAAAAATTGTTGCCTTTGAAGATTCAGATA
TAAGCTTACAAAATATAGATGAGTTATAAGAAGCAGGCCAAAGAAATACT
TTGGCTTGTATCTTTCTTTCTCTTACTGCTTTTTTTGTATTTTAGTAACT exon 9
CATGTTGTTGTTCCTGGTGATGCAGTTCAAAGTACCTTGAAGTGTATGCT BARD1_e09
TGGGATTCTCAATGGATGCTGGATTCTAAAATTTGAATGTAAGTGTTGGA
TTTGAGAGAATTAAGAAATGAATTAGACTAGTTTTGTTTTTCATGGTTAT

TAATGCCTGTGATTAAGGAACTTGATGTTAATTTTCTTACCTCTGGTTAG
TCACTGCATTTTGGAAAAGCTTCTggctgggcgtggtggctcaagcctgt
aatcccagcactttgggaggcccaggtgggcggatcacaaggccaggaga
tcgagatcatcctggctaacacggtgaagccctgtctctactaaaaatac
aaaaaaattagccgggcgtggtggcgggcgcctgtagtcccagctacttg
ggaggctaaggcaggagaatggcgtgaacctgggaggtggagcttgcagt
gagccaagatggtgccactgcactccagcctgggcaacagaatgagactc
tgtctcaaaaaaaagaaaaaaagaaaaaGCTTCTCATATTTATGGTGTCA
TTTAGATGTCCTTAGTAAATGTATATACATATGTTCTGGTTTTAAAGTCT
TTTTTCCTACAATACCAACTATTTCAATGAAAAGTAAATGGAGTACATAG
TATACTTTTAAGCCTAAGCGATTCATGTTACTGTTAAATGATATATTGAA
TTTAATAACACATGTTTTAAATATACTGCATGACATGTCTGTGAAATGAA
AGCAATTTATTGCTTTTCCTTTATGATTACCTGCATAACACCCTTTTGGT
CTTAGTTAATTTAATCCAGTATTTTTAGGAAGCTGTTGGGAAATTGACTG
CCTTTTACTACTTTAGTTGGAGTCACTATACCAGCTATCTCAACCTCCAC
TGAAAATGTATACCTGTTCCCCTCTTCTTGCTGATAGTCAACCCACCATC
ACCTTCAGAGCTGCCTGTCACCTTCCCTGCTGTCACTCATTTACATTTCT
ACTTGGATTCTCTTGGGACTTAAAATGAAAAGAAAGCTCATACACGATTA
TTTTCTACTCCTTAATAGTTAACATATCTACTTTTGATACTATGGCTAAC
ATAGAACAGTGGATTGACCTTTTTTTGTTTATTAATTGTTTACAAAAGAT
TAGTAGTTACAAAAGATTAGTGGTTACAGAGGAGGAATGTGGGGAGCAAT
acagttgcgtatcttgatgttagtggtggccatgtaacacaagactacct
gagataaaattatgcaaacaagtgcatttataaccagtgaaatcaggcca
ggcacggtagctcatgcctgtaatcttagcactttgggcgaccgaggcag
gcagaccagccaggccaacatggcaaaccccccttctgtactaaaaataga
aaaatgagccaggcgtggtggcacatgcctgtaatcccagccacttggga
ggctgaggcaggagaatcacttgaccccgggaggtggaggttgcagtgag
ccgagatggcgccactgcactccagcctgggcaacagagtgaaactccat
ctcaaaaaaaaataaataactagtgaaatctgaataagctctatggatcg
tattgaactcaatttgttggtattatgttatagcggtgttagatgttaac
attggacactgaaggaaaagtacgtgggacatctctgtacattcctttga
aacttcctgtgactataattatttttaaataagatgttAAGAAAGAACAG
TGGGTATATTTATTGAAACTCTTTATGAAGCAACTGATGGATTAAAATTA
GTTTATAAAAATGGTTTTATGGCTTTGTGCTTTGAAGGTTTTATGGTAGA
ATGCCTGATTTATCAAGATCTTAATTATGTTATTAATTTAATCTGTAGTT
AGAAGTTGATTTGGTTTGTAAAACAGTTTCCTTTCATCAATTGGTATTCA
TGTCATTTTCAAATACAGCTACCACTTCTTTTTTTGTGATGCTTGTCTCA
ACCAATGCAGTCTGATCCAGCTAAGAGGCTGTGACTAGCAATGCCACTCC
ATCTGTTCTCATCAATCCCTATTAATTAGTCCCTCCTAGTTATGATGAGT
TTGAGAAGTATAAGTAATTGGACTAATTTGTCTCACTTAGTTTTGAACTT
TCTTTTTCCTTTTAAATCTGTGAAAGTCTTATTGAGCCTCAGTGTTTAAA
AAATTCATTTAAAATGGTTTCATGATTAGGGATGCTTTTGTATCAAACCT
GTGCCAGAAACCTTTCCAGTGGTTATAAGGAGAAATAGATTTTCATCATA
GTAAGGTCGCAGTAATAAAGAAGCCATACGAAATTTTGAATGTTAAGACA
TTTACCACTTAACTTTTGAGTCTAGTTCTTGTGAATATGCTGAAGTGGCA
TTTGGTTACATATACAGGCTACATGATTTATTTCCATAAAATATGCCTTC
TTTGGGAAATGTGGGTTTTGTCACTTTAATATCTTGGCCGTGTCGTTTTT
ATAGAACCACTGATGGACTCGGACTAACTTAATAGTGTGCTATTTGGTGA
TTCTGACAGTAGTGAATAACATCCTCTAATATCAATGGTTCTTTTAGGAT
AAAGGATGGATGGAATCCCTTACTGAAGTTCATATCGTCATATTTTCTAA
TATAGAGTACAAAGAGAAACAGATTCAAAGTTTGAGATACTTCCTTTAAA
TAGTCACTGGGTTTACAAAAAGAAGGCAAGAACCTTCAAATAGTTCTTTC
CTAATATATATTTCTCATAGTTTCTATACTGTTCTCAGTGTCAAGCATCT
CAGTTTGATTCTTTGTTTGTTTTTTAAGAGATGAGGTGTCActcaagcaa
tcctccagcttcagccttctgagtagctggtactacgggtgcacatcact
gcacctggcTCAGTTTGATTTTTAAAAATGTTTCAGAGCTATTTAAAACT
ATTTGAAAACCATTCATATTAAAAGATGATAGGTGGACCAAAGCTTTCAA
AATTAATTTTAAAGATTTATGACTTTTTAAAACTAGGTCTCATGTTTGAG
TCTGTCCATTAACTTGCTGGTAGACAAAACTTCAGGATTAACTATTAGGA
AACTTTCATATTAACTTTTCATGGGATTTCTCATAAACCAAATAGATTCA
AGTAGAATTTTATTTGTAATTTTCTCTATAGATAATCAACACATTTTATA
AACTAAACCATTTAACAAAAAGAATTATACAAAAGATTTGGATCCATTGG
GTCCAAATTTAACTATGAGAAGTTTTCTGTTGAAGTGTTCTGTCTTAATT
GGTTAATATCTCTTATTTTCCTACATTGTTTATACAGAGTATCTTACAGT

GCCATAATTAATGAACTTAGCATGAGGATTCTGAGACTAAGCCATAATTT
TACAGACCATCCAGTCTATCTTATATTTCTTTTACAGATATTTTACAGAA
TGATACTTATTTTATATTTTACAGAAATAGGAGCTATAGATAAGAAAAGT
CCCTCCTTCAACATCAAAATGGTGAGGGAGACTCTACTTTAGTCCTAGTC
TGGGTGCTAATTTCTCACAGGCCATTAGCACCACCCTCATACAAGGTTGA
TTTTGGCATTAAAGAGGTGATAAGAATGAAAGAATAGAAGGTATATAAAT
AAGTCATCTTACGAATCTCATAGAGTATGAATAAGATGAAACTGTATCCT
AAAGATGCAAACTAGGCCAAGATAAAATGTTTTCTACTGACCTATTCTTT
TAGTACACTGCTTTCAAAATTTTTTTGTATCTTAGCCTTTTTTTCTAAAA
TAaatttaattctacatcttcttttcttatctcattatgaaggggagaac
agatggctgtcatcttttctttaataatctttcCCTCTTCCCCACTCTCA
TCTAAGTTTAAATAAAATTGCAGCTGTAGAAGTTGTAAAATTCCCCAGTA
CCTAAGGGAATTACTGAAGGAATGTGCCAGACATTTTACACTGGATTTAT
GTCTGTGAGATGGGGAAGGGCTAAATTGAGTGTTTTTTCATTTTTGTTTT
AGCTGAGGAGAGATGGACCCTAGAAGACAATCATTACATCATCATTTAAT
AAATAACCTCATAATCAGGCTAACACATTAATTCTTTTCAAACAAATTCT
AACAAGTTGGCTTCTTTAAATGGATACTGAGAGTACTATGGAATTTTAAA
ATTAAATGCAACTTTTATTCTATACAGACTTTGCTAATTATTTAAAAAAA
AACATTCGTATAAATTAAGATTGATTAGGCAACTTTATGCTGACAGAAGG
CTTCCACCAAAGATTGTCTAAGAATAAAAaaaaataacatcctttgatct
aatagtcccagttctgagaatttaccctacgcaagaaatacagatgaaag
aaaaagtatttgcagtgttacttctaacagcaagaagtagagaatataac
ctacatatgaaagattaggaaaatTCTGAAATGCCTGAAGATACTCTCTG
GAAAAAATGCATATTTTTAATATGATAAATATTAAGACTAAAAGATTATG
AAAAATAGAATTGTTTAAATTTTAAACACATCAAAATTATATCTTCACTT
TCAACAATATATAAATGTATGCATGCATGTGGGTAAAAAAATAATCAAAG
AGCAGTAAATTGGTGGGTTACTGAAGAGGACTATTTGTTTATAACAGTTT
TAAAAATAGTGTGTGCCTGTTAGATCAAATTTAAGTTCTCTAGGAATAAA
TTAATACGTTGAAATTTTCAAGTTGTAATAATAATTGGAAGTATTTTCAC
TTTTCTAACGATTAATTGGATAGGGAAGGGCTTTGGTCTTAATTAGGGAA
ATTAGAACCTGCATGTATTTATTCCTCCCATCTGATACAACAGGTCTTTT
ATACCTGTTTAATGGATACCCAGCAAATACATTTGAATATGGTGATTGTC
CTAATCAGAACAGAATTTTTTACATAAAAATTTGAACTTTTAATATATGA
CTTCAAAAGAACACTGAGCATGAGTTTTCTAATAATTAGGAGAATTTTTG
GACGTTAGACTGTGTTCGGTTTTTAACCATACCTATATAGAGACAGACAA
TTTTGATATTATTCATAATACAAGAGATAGACTATTTTGATATTTTGATA
TTATTATGCATAATACAAGGTCTTAGAATTGATACCCAAGTTTTGTCTAA
GaaaaatttatgatataataatatatttttGCAATAGAATTTAACCTGCT
TCTCAATGCATTAAGTAAAATAGCAAATTACACATCTTGTAATGTGAAGA
AATATATTTCTACATATAAATATGCTGATAGCCAGAATTTTTACTAAAAT
GTCAAAAATAAATAATTGCTAAATGATATTTATATGGAGCCAGAATTTAA
TGACTGTCGACAGTATTTAATGAGATATCTCAAATCTTAAATGGAAACAT
TTAGCCTTTATTTTATATTTTCAGCCTTTATTTCATTGTCCTTTCACTTT
TCGCTATGGAAGTTTCAAATTTCAAACTCATTTCTCTGTTCTTTTACTTA
AGTTTTCCAAAAATTGCAGTGATAGTATGCTACTGTTTTTTCATTTTTGT
CATTGTTGTAGGCTGCCTATTGTGATGTCCCTTTTTACTAAAGTTTAGTG
CTGTTATAGTTTACTCGACTAAAAAGTCTAAAATATAATCATTTGCAAAA
CACTTGTTTTGCTTTTACAATCTGGGTGTTGATTTTTAATAGAATCAAAG
GAAATTCTTTAATTTTTATTTTACTTACATACTGTTATCTTGAGTGACAC
ACATCCCTAATTTAAACATCTTTAGTTTAGATCTAATGACATACTGTAGT
AGAACATTTGTTTTCATATAGAATAGTGATGTTTGATTCTGtattttaat
tttttttttttaGGATATTTTGTATTGACTAAAAGTGGTTAATTCTGATT
CCCATAGCTATTTCAGTTACAAATAGCCATTAATTCCAGAATAATAACAA
CCCTTTTTTCCCCTCCctcactctgtcacacaggctggactgcagtggtg
caatcatagctctttgcagccatgaactcctgggttcaagtgatcctcct
gcttcagcctcctgagtagctggaactacaggcactcaccaccatgcctg
gctaatttttttttttctttttagttgaggcagagatctcactttgttgcc
caggctgttcttgaactccttgtttcaagcggtcttcctgcttcaccctc
ccagagtgctagcattacaagtgcgagccaccaggtctggccTAAAAATT
CTTTAAAATGTGGATCTGTTTACTACTCTGGGATTTGAAGAGTGAGAAAA
TTCTAAGTATGGCTTTCCATGCAGAGTTAATAATCGTCTCCTCCTTTGGA
CATTTAACAGATCTCCATTTTGAAGTTTTCTTCCATCTTCATTGTATTCT
CTCATCATTTTTGTATAACTGTGGATTCAGGAACACCCCTCCTTTTTTTA
ATTTCCTTGCATTTGTGTTATCTTTTTTTATTATAGGAAATGTAAGCTTA

ATATAAAACCATGACCATCATCAGTGGTCCATGCAGCTAAAACAGAAATA
TACATCTACTGATGTATATGCTTTTATGAGGTAGGAGGAGAGAGTGGTTA
TTAGCAATTTAGAATAAAAAAATACCTTCTGTACAGTACAAGTTTCTTTA
TAGAAAAGTGTTGTAGCCCTGGAATTCATAAGCGTCATTATTATGCAAAA
ATTCCAGGCTTTGCTAAGGTAGACAGAGCTACCTTAATGACTTTGGGTTA
ATCAGTACTTTAGAAATGGGGCTGCTTACCTATATGTAAAAAATTAATAA
GATCTCAGGAAAAAAGGAAAGCTTACATGAAATGGAGAACATGATCAGGA
ATACACCTGGGCTAAAAGAATCTCTAGTTGTAATAGCTAAGTAGTTCATC
CGTTTGTTTCTGCGAAGTGATAGCTTCAGTCAAAACCTTCAACTATAAAG
GAACATATGAAAAGTAACAAAGATGAAATAAAGCATACATTTTATAAGGT
TAGTGGCTTGACACATTTTCAAGTATGTATCATTCTGGCATATGCAAAAG
TTATGTTTACGTAAACTTACTCATGCAAGGTAATTTTAGTAAGGAAATTA
TTAGCCAAGAAAGCTAACTAAAATGTGGTCTTATATTTGTATTTTTGCTT
TTTTTTTCTTCAAAAGGAAGTAGAAGAGTTCATCTTTCTACTAAACTGAT
GGAATAATTATGGACTTTATTAACTGTTTATTACAAATATAAAATAGTTT
AAATCTTAAAATGTAACTGTGTTTTAAATGGAAAATAGGTTAAGAATTCT
TGCTGCAGATTGCTTAACTTTTATTCAGTAAATAAAACCTTAAAATGTTA
GTCATTATTTAGGCTTGGGTTAATTAAGTAATGTTTGGCTGGTAGATTCT
TCCAGTGCATTGATAATGTATCACCATTTGAATTGCTGAAAGTTTATTTT
AAAGCATTTCATTCTTATTTACTTTTCAGTAAATCAACTCAATTGATTTG
TCTTAAAAAAAGGTTCAGTAGGAAGTTGATATGAATTTGTAACTGTGGTA
TTGTCTCCGGAGTAGGTCATCATTCTGAAACTCACAGTTTCAGATAACAA
GTTAGACAGTGGCAACCTTAGCATGTCGGATTGAGTAAGCACATTGATCC
AACTAAAAATAAAGATGAACAAAAAAACTTGATCTGTGACATGGaggata
tttcagttctcatctcagttatgtcactgagaaagccacttaaatctctt
gggacttaagtttccttatctataaaagaggttgattagaCAAAGCATCA
AAAGTTTCAAATCGGCAGGCTGAGGACTCTGAGGGCTGAAGGAAATACAG
TGTCTGGTTTTGTCTAACataattaatttttatatatttaGGCATGATGT
TTGTTACAGtctccatagcttcctacacctcacaccaggttcttcacac
atttatgtaatctacctggcttttgtaggcatttgagtcgcaatccttgA
AGTAGTTGTCACTCCCAGCTTTGAAAAAATTCTAGAGATTTTCTATGCTA
TAACAGCAACTTTAACAAAACACTTTGCCACCCTTCAAATGTAGTTGTAG
ACCTGGAAACATCCTTTCTTTTCAGGAAAAGAAAAAGAGCTACTTTCATT
CTTCCTTAGTTAGTCTTAGGTGACACAGGATTCTGCAAATTGTTGCTTGG
AGTAAAAGCATATTAGAGAAATGTATTTGTTTAACTCAATCATTGTAACT
AGAGCATTGGATTTGCTTTACTTTACAGAAACTTACTACATAACTCCCAT
GAAACAGAATATTTAATGAATTAAGACTTACACTGGCTCCTTCTCATGGA
AGTACCATGCTGCTCTGTTGGTATCTGCCATCTCATTTTTTTGATGCCAT
ACTTACTGGACTGTAGTATCTATTAAGGTTAAATTCTGTTGATTGACTAA
TGCCAGCATGTTTTCAGGAGGAATAGATGCCATTTTAATGTGTTTCTTGT
AAATAATCAGTCAAATAAACCCATAAAATGGAGAAGCAGCTGGCACAAGC
ACAGCCTACTTAATTCTGCAATTAGACATTAGCCATTCATTCGATACAGT
AGATATCATAAGAATGTTCAGTCCTTGGCTTTCTGTGCCACCATATGCAG
TTAAAGATACAATTATGAATACTCTGTAAGTACCATTGGGAAGGAGATTT
GGGAAGGGGAATACCTGCTTACGTAATGAGGAAAATAAAAAAATCACATA
TAAAATAGTGAATTTGAGTTAACCATTTCACTGGATTCCTGATGGTTTTT
TATTTGATTTTTAATATAGAAACAATATATGACATAATTCCAGCACACTG
GCAGTGCTTCTAGTATGGTATTTTGACCCAGTCAAGAATTTTGAGTCTCA
TTTTCAATGtataataaaattaatatttatattaCCTTTATTAGAAAGGT
TTTTAATTCAGTGAGTGGTTATTAAAATTTATGCCTTTTTTAGAGTATAA
TTTTTATTATATGTAAACAATTTTCAAAGCACATTTATGATAATTTAAAA
TTATAAGATTCGGAAATTCAGCTTAGCAACCTTGGGTGAATATGACTTTC
ATATTAAAAAAATGGGGGAGAGAGATCTGAGAGCCAAATAAAACACAGAT
AAAACTAACAAATTAAGATTGACATGATGGGTAATCATAGACGTCCTGAG
TGTGAGTGTTGAATTTGAATTTGCATTAATGACCCAATTCAGGAAAGACT
CAGCGTCTTGACAAACCTCTGCTTTTGGAGAGATGGAGTATTCAGTTGTT
AGTCTTCTGACCTTTTTATAATAAACAACATCTTAGAACACTCAATTTAA
GCAGCTTTTAAGCATTTGACATATTCACATCATGCTAAGATAATACCCAA
GGTTTATCTTGTGTACCACTAAGTTATATTTcagtgtgaaatacacagcc
tttagaaaactaagcagatttaggttcaaattcccattctgccacttaat
ggctgcattcagctttaagcaagttgacttcttagcatttcagttccctc
ccctgaattggaagtaataaacagtaaatcctcattgtttgtggattctg
tgtttgtgagttttcttacttgctaaaatttttcttgtaatccccaaatc
aatactcacagtgcttttgtagacatgtacagagtagtgaagaactttag

ttgacaaacaagtgtgtccccagctgaggtcaaacaaggtgatactttac
ctcctcttttcagctctcacgctgtaaacaagtggccctttcacagtctg
ttgatgccatgtttttgcatttttgtgtttttgttgctgattttattat
ttaaaatggcctcataatacagtgctgaagtgctgttttgtgagtctaag
ctcaagaagatggtgatgtgcctttcagggaaaatattaaatactgtgta
ggataagctttgttaaggccttaattgtagtactgctggccctaagttta
atcttaatgagtcaacagtatacaataaataatgtgtataaacagaaaca
tacataaaacaaagttaagtattgatcagttgatggaaatgctggagcca
caggcccacagaaatgtaaccctgtatttcctttaacagcagtgtttcat
tattcactaatcggtgttcatagcatctttatagaacataactactgtag
ataatgagcattgactatactactcttgctggatttggggaaggatACAA
GTACTTTTGGTAGCTGTTATCACAGTAATTTTGATCAGGATCTTTTCTTA
GAACAAACAGTTGTGTCCTTGGTCATTTAAAGTTAGAAAAGTAAAAAGAA
AACAGAATAGAATAAATTGGTATTTTAAGAATTTTCTTTTTCTCCTTTGA
CCAGAAAATATTGGTGTGTGATTTTTAGCATTGGATTGGTTAGGCCTTAT
GATTTATGAGTATTTTATTCTAGATTGTGGAAAAATTAAAGTTTTCAGAG
CACTGCACAATGTACACGTTCAATCTATAGTAAAGTATTTTGCTCATTGG
ATGAATATTAGGTAACATAGCATTCATAGTTTTTATAAAATGATACTGCT
CTCATGGTTCTCTATTAATTTATAAGAAGAGAGTTCTGTCTGATAGTATT
TACAGGTGTAAAATAAACTTATATACAATCAAATAAATTCTACTCAGGTT
CTACTCAATTTAAACCCAGAGAAGTAACAATGGAACAACTTATTCCATAT
TTTCTTAATGATGTTCTACTTTTTCTTTCCATATTTAATATTGTGATTTC
GTATTTGTTTAACAAGAGCTATAAATGGCTAAAACTCTGGCTGTGTATTA
TTTCCTGCTTTATTAGTATTTTTGAATACCACTTCACTAATACATGTAAA
TCATTAATACTTCCAGCatattttttttaatatttaaaatatattGGTCCT
CCATTATTTTCAGAAACAACTTTGCATTCAAACATTTTATTCAAAAAACC
TATTATCTTTGCTTTTAACATTGAAATACATGCAAAATATAAAAGAATAT
TTTATTATCTTTCTGGCAAGCAAATCTGCTCCCACATTTGTTATTCTTTG
AAAGGTTAGCCATTTTAAGTAGGAAAAGAGGGAAAAGCTGAATCTGAAAA
ACGAATCTTCTCATGTTTGTCTATGTATAAGCTATTAACCTTTTTTCTTT
CACTGACTTTGTAACAGAAGTTTACGTAATGTGAAGTTCTTAGGATTTCT
GGACAAGAGTAGGGAGTTTTCTTGTTATTAGATATACCATGATTGAGATT
ATCTCAGTGGCATCCTTAATATCTTTAAAACATGGATGTAATTTTAAATG
TGCTTGAAAAATTAAATTTGATAGTGGATTGCTTGTTGACAGAGGAAAAT
AAAGCAATAAAATTTCATTTAAAATTAAGCACTCTGAGATAGAGCATACA
ATATTAATGAAAGTTCAGGATTAGATTATTGAAATAAAGTGACAGGAAAC
AGCAGGAATTTTCTTCTTAGGGAAGAGATTATGACTTCTCTTGTACATGA
TAGGTGTTTAACAcagtttgagcattcctaatctgaaaatccaaaatgct
gtaaaatactaaactatttgagtgccagcacaacacacacgacctaacat
gatggctcacagtctaaacgcagacacaactttatgcacaaaattattaa
agatagtatataaaattatcttcatatatgctttatctgtataaagcgta
tatgaaacataaatgaatttctttttagacttgggtcttgttgccaaga
tatacctaattatgtacgtgcaaatattccaaaatccaaaacaaaattga
attttaaaacacttgtggtttcaaacacttcagataaggatacttggcc
tATAAATATGATATaggaagagcgccgaagtggaagtctgttgctttagg
aagtggttcactttaactttgctattagtgatcttagcaagtcactttat
gtttctgcacttgactgtgcagatctttaaaacaagaaggtaggactagc
tcttcagggtcttttctacctcgtaagttatttgattctaGGTATTTTCA
AAATTCAAGTTACAAGATTAGAATAAATAAAACATTTTATAAAGAGGACT
CAAACACATTTTTTTTCCCTCAGAAAGTACCAGTTTTTTCAGTTCTGTCG
CTTACTCCAAATTCTGCAGATGTGGCTCCTGGAAACAGTTGAAGTTTTGT
TATTGTGTCAGTGTGGGGTAATAAAGAAGAAAGTTTAAAACCGATTATGC
CATAAAGTCTGAGTTAGTCTAAAGTGGACCATTACTCACTCTTGGAAAAC
ATTGCATTTCTTTTTCTTCTCCAGTATAAGTTTTTGCTCTCAAGATAATC
ATTATAGGTCagcaaggtgtggtggttcacgcctgtaatcccagcacttt
gggaggctgaggtgtgcagatcacgaggtcaggagatcgagaccatcctg
gctaacgcgatgaaagcctgtctctactaaaagtacaaaaaattagccag
acatggtggcgggcgcctgtagtcccagctacttgggaggctgaggcagg
agaaaggtgtgaacccaggaggcggagcttgcagtgagccgagattgcgc
cactgcgccccaacctgggtgacagtgcaagactctgtctcagaaaaaaa
aaaaaaagaaaaaaaaaTCATCATAGGTCAAAAGACATTTTACTCATACA
CAACTGAATCTTAATTTATATTAGATGCAATCGCAGGACAGTTCTGGGTA
GCAGATTAACTGTTGGTTCTTAGCCACCCTTTAGTCAAGAAAGGAGCCCT
TTTGCAGGCATATGCAGTTTACATGCCGAGTAGTGGTTCTGCTCCTGTGT

TGGAATCAATTTACTGTTGAAGTAAGTAGGGATCAGCTGGACAAAACTTT
ATCACTAGCATTTTTCAAGAGGTTTCCAAAACACTGTAGTATATCCAACA
TATATGTCTAATCCTCAATATAAAGTATATGTTTATGCAGCCATAGATTT
TTTTCTGATCATTTTAAGCCATTAGATATCAAGTGATAACAGGATGTAAC
AGTTTGATATTCAAAACTATGTGTAACTCTGAACTCTTACAGGTGCTATA
TAATTACTTATTTTTATTGATGTGGATACAATCTGTCAATGCTGTGTCCA
TAGTGAATTTTTAAAAACTTTTGTTCCTTTATGTTAACAACACTAGACAG
TGAAGGGGATACCTGAGTTATTGTTCCAACTTTCCCGATACCCTCTGCTA
TTAAACTCCTGTTCTATGGGAAAGTGTATGAAATTCTGAAGAACGCATAG
GTGAATAATATATGGTTCCTACCCTCAGTGATAAAATGAAGTATTGAGCA
AAACAACCTCAAAGACAAATAGGAAATTGTATGGAGGGTTTGTTTTTATG
ATGAAGATAATTCATTGTAGTATCTAGTTTTTAGAATGCTTCTTGCATTT
TATCCACAGAAGCCCGGTGAAAAACATTGTTTTCATTTTTTATTTCCATA
GGCAAGGAAGCTTCCAGAACAAGTAATTGGGATAGCCAAGTCCTTCAACT
GCAAATCTCAAATTTAGTTTAGTTATATGCCATCAGAACAGAAGCATGAT
AAATATATATACATGCCATCAGTTCCCTCGCTTGGTGGTAAACAGGCATT
ATCCAAGATGTTGTCTCTAGCTGAAGCTGACCATGTCCTTCATGATTGGT
CCTGGGAATCCCAAGGGTTCCTTTCATTTCTAGAGCCTAGCGAGACAATT
GGTACTGGCTGCCAGCCAGTGGCCCTACTTAGGGATTCCCCACCCAGGTG
CCAAGCAGTGGAATGACTCTAAACAGTGCCAGGCTGAGCTGTCTCTATTG
CTTTTGCTTTTTAACCTGAAGCTGTGAACAGACTTACTCAAGCCATCAAA
AGAATGCTTTTTTTTTTCCTGCAAAGCCCAATCAAATCATCTCTAGACTA
GCAACCTCAGTTGTGTACCTGTCACATGCCAAACCTATCCCAAGCTCTGC
TTGTTTATTTTAGGAAGCGCTGTTGAACCCCTGTTGTGTGGCTAGCTGAG
CTTGGTGCTGTAGACTAAAGCACATTCCTTCATGTCAAATCACTTACAGT
TTAACAGACGATTAGACATATAACTGTCAAAATGAGCAGTATAGATGGTA
AGTGCTCAGTTTAGGTTATTGTGTCATGGACTTTTTATTCACCTTAATTT
TGGGTAATTGCTATGAGTGGAAATGTAGACTTTTATTTTTGTCTTTGAAA
TAGTATCCTGGCTTAGGTTTTTCAGAAAGGAGATTAAAATTACAGTTAGT
GTTCAGTACTAACTTATGGCTTAATCCTCCAAAGAAAGAGTTTTTTAAAA
TATTTTCTTTATATGGGAAAACCAGTTGTATTACATTTTGTTTTGGCATA
AGTAAGATTTCTGTTTGCATTTTAGAATAATACTTAAAAACTGCCATGAA
GAAGAAAAACCACTTAGGTAAATTGCTTGATTTTAATGAGAGAGATATAG
TGCTCACTTGATACTTAGTTTGCTTTAATTCTTGTGTTTTTGTCAGGGGT
AAAAGCATGTCTACGAAGAAAAGTATGTGAACAGGAAGAAAAGTATGAAA exon 10
TTCCTGAAGGTCCACGCAGAAGCAGGCTCAACAGAGAACAGCTGGTATTT BARD1_e10’
TTCTTTTAATACAACTTTCATTGTTCTTATTATGACATACTATTATTATC
ACCATCAGGAAGAACTTCTGCCCTTTCAACAGCTACAGGTGACTGATTAA
AATTTTAATTGTGCTTATTTCAAGCACTTGATTCTGAAAGATGATCACGA
TGAGCAGTAAAATCCAGAAGGTAATAATTTCATACTGTTAATGGATTTTT
GGCATCTTGAACATTGCCATAAACCTTTCAGAATCTGAGGTAAATCTCAG
ATACAGGAAGTAGCTTGAAAGAAGACTTACAGCTGCTGCTTGGATTTAGT
TACCATATGTCTCTATGGCCACATATTGTAGCTTTAATGGATAATATCGC
ATTATCCTGTTGATATTATATAAGTATATTAGAAGTCACAAAGAAAATTT
CCATAGAAGGGAATTATGAAACTTTTTTTATTTCCAACGAGCATACGGAA
GTATGTTTCATAGCTAATTGGATCCCTAGCCTCAGCACAAAAATCTTTTG
TGCCCCGTGAATACATTTCTGGAACCCTGGAGGGCACACCCCCATGGTGG
CTGCCCTGGAGACCTTAGGTTGGTAATATGTAAGGACCTGAATGTGGATG
GGCAGAATTGGATAAAAGTCCACGGAAAAGATGTTACTCTTGTAATTTAA
TAATGTTTAGCCTGGTGTCTCTGAAGCCTATTTCAAATAAGCTAGGAGTT
GTGGAGGCTTTAAGTCCCACCAAATAAGCATAAACATCCTGATGAAAAAA
GTTTGATGAATAGTTTGTTTTTTTCTTTATACCAAGCATATCTAAAATTT
TAGAAGAGTGAAAAGGAACCGAGATGGTGACTGAATCTTAGGGAAAAAAT
TGTAAATAGGAAGCCCCTATTTGCCTAAGTATTTTTCTTGATCCAGTTAG
TATGCTTGAAATATAACTTGTCCCAGCACCTCATTAAGTAGCTTCTTAGC
TGCTCATAATTGTTAcagatggagcattcctaatccaacatctaaaatgc
tccaaaatccaaaactttttgagctttgacatgatgccacaagtggaaaa
ttccacacctgacctcatgtgacaggtcacggtcagaacacagtcaaaat
tttgtttcatgcacaaaattactgaagatattgtataaaattacttcagg
ctatgtgcataaggtgtacaagaaacaaacgaattttgtgtttaggcttg
agcctcatccttaagatacctcatgtatatgcaaattttccaaaacccaa
aaaatttatgaatctgaaatgcttctcctccaaatgtttcaggtaaggga
tattcaacttgTATTTTTATTTTCCTCATTCATATACAGTGTTTTTGAAT
ACAGTATTTTGATCTGCCTTTAACAAATGTTTTCTCATTATTTCAGTTGC exon 11

CAAAGCTGTTTGATGGATGCTACTTCTATTTGTGGGGAACCTTCAAACAC
CATCCAAAGGACAACCTTATTAAGCTCGTCACTGCAGGTGGGGGCCAGAT
CCTCAGTAGAAAGCCCAAGCCAGACAGTGACGTGACTCAGACCATCAATA BARD1_e11
CAGTCGCATACCATGCGAGACCCGATTCTGATCAGCGCTTCTGCACACAG
TATATCATCTATGAAGATTTGTGTAATTATCACCCAGAGAGGGTTCGGCA
GGGCAAAGTCTGGAAGGCTCCTTCGAGCTGGTTTATAGACTGTGTGATGT
CCTTTGAGTTGCTTCCTCTTGACAGCTGAATATTATACCAGATGAACATT
TCAAATTGAATTTGCACGGTTTGTGAGAGCCCAGTCATTGTACTGTTTTT
AATGTTCACATTTTTACAAATAGGTAGAGTCATTCATATTTGTCTTTGAA
TCAAAAAAAAAAAAAAAGTCTAATGCCAGATTAGGAATTCATGTTATGTT
TACCATTTAGAAGCTGGGATTGCTTTTAAAGGTTTTTCTTTTTAAAATTG
GCATGTTTTTGATTTATCATGTCTTTCTATTCAGATTATTGGGTATCAAA
GATTAATGAGGACACCAGAATCTTGGTTAAATAGACAAGTGGTATCATTA
CTGTTTGAGTCTTTTAATATTCTCCATACCTGCCACCAGTGAAAAAACTT
GCCttttttttttttttttttttAGTAAACAGAATATTATCAAACAATTT
ATTTTGGCTTTATTGAAAAAAGAGTATTTGGTCTAAATGTGCCACCATAG
GTGTTAAATTCTCCTATCTGCAATTGTCTTTATCCTATATTGTGTTCATT
TCTTTTCTTAATAATTTACTTTGTTGTGTGTTTCTACACTTTCATCCCTG
TTTTTTATCTTGTATATCATCAGGAAATTGTGATTTAATCATTAACATTG
GTTTTTTTGTGTGTGTGGTAAAAATCAACACTAGGCTCATGGTACATATT
TTTATTCTGTACATTTGCTTGTAACTATCAATTTGTAACTCTGTTTATCT
ACTACATGTGTATATATACTTAGAGCATTTTCTCTAACACATTTTAATGT
TAGTATTTTTTAAAAGGTCTGACCAGTCTAGCAAATTGTCAGTCCAACGT
CATTACTTTAAATTAAGAAGCAGTCTTCTTCTGGTAAACCTTGTTGGTAT
TTGTAAAATAATTTTGAAGGTCTTAATTTCTTCCTTTGTAAAAGGAAAAG
GTTTTTTTTAAAGTTTTTAGGTTGGCATGGAGGCAGAAGTTGGTGATTAC
TTGATTTACAACAGATTTTTTCCAGATCATACAAAAGGCCATACAGTAAG
TATAGAAGTAGGTATGGGGAGGGCTTACTAATATCAAATAGGCAAGGCCT
TAGTGAGTGGGCAGGATACCACCTGAGAGTGGCCAGATGTGGGGAGGTTA
CTCTGCTCTGGGTGCTCTCATTCATGAATCGACAAGGATACATTAGATTA
TTTTGAAACATTTTTTTAAGAAGCAGAATTCTTTAATAATTCCTTCCTAG
ACATTGAATATACTTATAAAATTAAAGACTTGGGGAAGGAGACACTGAGA
GACTTGCCAGTTTGGTTCCTCATGAACAAAAGAGGACAGTTTGATAACTA
CCAGAATAGAATATCCCTAGTTTTAAAATAGTGAGAATCTCTGAAGTTCA
TCAACATCTTAAGATGCACTTACTTGAAAGTTTGAGATTCTGTTTATCAT
TTGAAAACACATTTTGCTTTAATTCTTTCTTTGACATGTTGTTTTTTCAT
ATCAAGAAATATATGAACAAAATAATAACCTTTTGACCCTGACCTTGCTG
GGTGAATTAGCTCTGAAACACTCTCTACAACCAGTAATGCATTTGTCCCA
CATTTCATTCTGATAGAAAATGAACACCATAGCACCAAACAAAAATCCGA
GGCGTTAGATAATGTCTGGATTAAATAATTTAAGACTCTCTAGGATTTTG
GTTGTCATTTTTTATTTATAACAGACTTTAAGTCACTTTCTGTTGCCTCA
TAGGTCACATTTTAGACAGGTTTGTGTCTGTTCCTTGCATCTGAATTCCT
GATTGTAAAGACACCTATGAGGTCTCTTAGTTTTTGTCATTCATTTTCTT
GGTTTATCACCCCTCCCTTCTTTTTGTTGTTTTTCCCTGACTGTTAAGCA
GTTTCATCTTTGCTTTTGTTAAATATTTGACAGCAGTTAGTTTGTGTTAA
GCTCTTGAAACTTGTGATTGTACTTTCTGTGTAGATATACATGTAATTAT
TTTTTATTTTTCAATCATAGATTCAAGCTTCCTTCTTTTTTACCACAAAT
CATTAAAGTTATTTGTGTTTCCATATACCTGTGTCTTGTATAAAATTGGC
TTATTCTGTGCTGTTGAATGAGGCTCAACATGACTTGGTGAGGAAGTCTA
TTAACTAACAAAAGCTTATCTTTTTTAACATAATGCtttttaattaattt
tgaataaaaatatttCTAAAGTGTACTAGATACTTTATTACCTTAGATTA
TTCCGAATACAGTATAACTTTGATAGTTTGGAATAGTCATTAAGAAACAA
TTACACACTGATTGCTTTGTGTCTCTAAAAGTGAGAGGCTGGTAGCTTTT
CCACATTCTCATGGCTATTTTCTAGTTCTACTTGAATTTATAACTGTTTC
CCTTTTTCCTTGACAGCTGCCACTTTGTAGCTATTTTTCTGTCTCTGCTA
ATACTTTACCATATCTATCTCAATTGTTTTTTCTTTTGACTTGCTGAAAA
ATAGAAACCAGATGGGAAGTATATTAGCATTATGATTGAAATAAGGGTAA
ATGAGCAATGTGTGAAGGTTTTCACTGACTTCACCTAAAAGATAGTTTAG
CTACTTGAATTTTAGTAAATAGAATTTTTCCTTTATTTCATCGGTCCCCC
CACCTTTTTTTTTTTTTGCACCTGCCTTGTAAATTTAATAGTTAAGTGAC
CTCTGCCTAGAGGATGATATTTGGGGAGGTTTGATGTTTCCTGTGGGAAT
AAGACGATTCACAGGTGAGAGTGGGGCCACATTAGCTGTTATTGTTTCCA BARD1_f3’
TGGGTCAGTGTGGAAAATGCATTAATCATATTCTAAACGTTCATGGGCCT
CATTACAGTCACAATTGTCTATTCTGTTTCCTACCCTGAACACATTAAAA

TGGTAGGAACTAATGCTTGTCTTATTTAATTACTAAAAGCCACCATTTTC
TTTGATAGATTGAGCTACAGATTGTAAACTTCATGTATTTCTTTATAAGT
CAACCCTTTTCAAAGATACGCACATCAAACTGAATGAATAAATAAATATT
GAGAAGTTGTCTTCGTTTAAGAGCTGTAATTTTTTTTTTTTTAGTCAACT
AATAGTCTTATGCCTGGCTAATTAAGCTGCAGACATTCTTATGAATTAGG
GATTGTTGATTATTTGTGTAGCTTTCTAAGGCACAAGGTGCACTTTCTGA
TACCTGTAAGTTTCTATTAGAATGGTGATGTGAGAGAAGTGGGAAAAGCA
TGTTCTTTGACTAGGCTTAAAGGGTTAATGACTAATACTCCAGCCCCAGG
GAGGATCCTGAATCGGCTTTGAAGTTAGACATGGGTTTGTTAACAATCTA
ACCTGTATCATCAGTGATAAAGGGGGGGAATACAGTTCATTTCACCTGAG
TATAAAGATGTGTGAAAGTGCTTAGCACACTGTAGGCATTCACAACTCGC
CTTCCAGGACCCCTGTGAGCAAGCTCATGAAAAAGTGACTGTTGGTAGCA
CAGCTTGTGTCAGTCCTGAGTATCCTGGCTCTTGTATCCCATGCACAAGA
CTGCTGGGTGTCCTCCTGTATTCACTTTACTGGTTTTAGCCTGAACAACA
ACCAGAGCCAGGCCCTAGTTTCTTATTTCTGACTTCTCACCATCCCATCT
GCACTGGTAAAGTTTGCCCATCTATGTAGCCACTCCTGCTCATTTAACTA
AAATGTTTAGCTTTCATCCCCTCAGTGTATTTACTGTGGATACCAAGATT
GCTTAAGCCTCCACACAGAAGTGGGGCACCTTGGACAGCACTGGCAAAGC
TTCTACAGTTACCATAATATCATAAACCCTTCCGCCACCTCCACTTGAGG
CCTAAAACAAGGATGTTTATCTTTTCTGCCTTAGTTCATAGTTTGTGAGA
CTCATATTTTCATTAATACCACTAATTTGTCAAAACACACCTTATTTGCC
TGCTGTGTAAGATAATGTAACCTAAAAATAAAGGTCTTTTTCTGAATCTT
ACTACTGTAAATGTTTGAGCTTTTAAACAAATCTGTTTACAAAAATATTA
CATAGTTCAGAAATACAAAAAGAATTTCTGCAGAATACAAAAGCACAAGG
AAGAAAACAGGCACAAAATCCAGGGACCGTTAAACTGACAAACTATTTTG
TGTGGCCTTCATAGTGTTAAAAGTACATCAAATGCTGTTGACAGGGGACA
TGAGCTTCCATTCACCACATCCTCACCACTTGTCTCTCACCCCTTACATT
TATTCTTTTAGTTGGGCCTTGGAAACATTGACTTAATCCACAACTCAGGA
ACAACAACTATTAACATTCTTGTCtttttctttccccataagttattggg
gtacaggtggtattgggttacatgagtaaattagttgtgatttgtgagat
tttggtgcacccatcacccgagcagtgtacactgcaccatatttgtagtc
tttatccttcgccccccctcccactcttcctcccaagtctccaaagtccat
tgtattattcttatgcctttgcatcttcatagtttagcttccacatatca
atgagaacatactgtgtttggttttccattcctgagttacttcacttaga
ataatagtctccaatgtcatccaggtcgctgcaaatgctcttcattcatt
gctttttatggctgcatagtattgcatcacacacacacacacacacacac
acacacacacacacacacacgtatatgtatatatacacacaccacagttt
atccactcattgactgatgagcatttgggttggttccatgattctgcaat
tgtgaattgtgctgctataaacatgggtgtgcgagtatgtttttccagta
atgacttattttcctctgggtagatacccagtagtgggattgctggatca
aatggtagttctacttttagttctttaaggaatcttcactgttttccata
gtggctatactagtttgccttcccaccagcagtgtagaagtgttccctgt
tcactgcatgcatgccaacatctgtttttgattgtttaattatgaccat
tcttgcaggagcaaggtggtatcacattgtggttttgatttgctgatcat
ttctgatgttgagcatttttcatatgtttgttggccatttgtgtatctt

Supplementary Table S1

The MLPA probe set designed for the analysis of large mutations in the *BARD1* gene.

probe	5′ half-probe							3′ half-probe							5' HPL	3' HPL	total probe length
	5' PSS	length	5' SS	length	5' TSS	length	Tm	3' TSS	length	Tm	3' SS	length	3' PSS	length			
ctrl_1 [chr22]	GGGTTCCCTAAGGGTTGGA	19	cgctac	6	GGCCCAGATCACCGAGGAGGA	21	75.6	GGCAAAACTTCTGGCCCAGAAG	22	71.0	ac	2	TCTAGATTGGATCTTGCTGGCGC	23	46	47	93
BARD1_e10'	GGGTTCCCTAAGGGTTGGA	19	cgctacta	8	CTGAAGGTCCACGCAGAAGCA	21	70.8	GGCTCAACAGAGAACAGCTGGTATT	25	69.1		0	TCTAGATTGGATCTTGCTGGCGC	23	48	48	96
BARD1_e11	GGGTTCCCTAAGGGTTGGA	19	cgcta	5	CTCAGTAGAAAGCCCAAGCCAGACA	25	71.7	GTGACGTGACTCAGACCATCAATACAG	27	70.5		0	TCTAGATTGGATCTTGCTGGCGC	23	49	50	99
BARD1_f3'	GGGTTCCCTAAGGGTTGGA	19	cgctactac	9	GTGAGAGTGGGGCCACATTAGCT	23	71.4	GTTATTGTTTCCATGGGTCAGTGTGGA	27	71.5	c	1	TCTAGATTGGATCTTGCTGGCGC	23	51	51	102
BARD1_f5'	GGGTTCCCTAAGGGTTGGA	19	cgctac	6	CTGGGACCTGGATAGACACTTGGATAT	27	70.5	CAGCTAGAAAGCTACGACAGGAAACCA	27	71.1	tac	3	TCTAGATTGGATCTTGCTGGCGC	23	52	53	105
BARD1_e02	GGGTTCCCTAAGGGTTGGA	19	cgc	3	CAGTACTAACATTCTGAGAGAGCCTGTGTGTT	32	71.5	TAGGAGGATGTGAGCACATCTTCTGTAGG	29	71.3	ac	2	TCTAGATTGGATCTTGCTGGCGC	23	54	54	108
ctrl_2 [chr1]	GGGTTCCCTAAGGGTTGGA	19	cgctactactat	12	CAGCTGGACGAGTACCAGGAGCTT	24	72.8	CTGGACATCAAGCTGGCCCTG	21	72.7	aactaaatctac	12	TCTAGATTGGATCTTGCTGGCGC	23	55	56	111
BARD1_e03	GGGTTCCCTAAGGGTTGGA	19	cgctacta	8	CAACTGGACAGCATGATTCAACTTTGTAGT	30	70.6	AAGCTTCGAAATTTGCTACATGACAATGAGC	31	71.1	tac	3	TCTAGATTGGATCTTGCTGGCGC	23	57	57	114
BARD1_e06	GGGTTCCCTAAGGGTTGGA	19	cgctactact	10	GAATTATTGCTCCAGCATAAGGCATTGGT	29	71.4	GAACACCACCGGGTATCAAAATGACT	26	71.0	ctaaatctac	10	TCTAGATTGGATCTTGCTGGCGC	23	58	59	117
BARD1_e07	GGGTTCCCTAAGGGTTGGA	19	cgctactacta	11	GAAAGTATGAAATCGCTATTGCTGCTACCA	30	70.3	GAGAAGAATGAATCATCCTCAGCTAGCCA	29	71.9	aaatctac	8	TCTAGATTGGATCTTGCTGGCGC	23	60	60	120
BARD1_e09	GGGTTCCCTAAGGGTTGGA	19	cgctactactattagt	16	ACTCATGTTGTTGTTCCTGGTGATGCA	27	71.6	GTTCAAAGTACCTTGAAGTGTATGCTTGGG	30	71.5	taaatctac	9	TCTAGATTGGATCTTGCTGGCGC	23	62	62	124
BARD1_e08	GGGTTCCCTAAGGGTTGGA	19	cgctactactattagtagaattga	24	ACTGGGCAGCGTAGGGATGGA	21	71.8	CCTCTTGTACTTATAGGCAGTGGGCTG	27	71.4	caaactaaatctac	14	TCTAGATTGGATCTTGCTGGCGC	23	64	64	128
BARD1_e04.1	GGGTTCCCTAAGGGTTGGA	19	cgctactactattagtag	18	GTGGTTTAGCCCTCGAAGTAAGAAAGTCA	29	71,5	GATATGTTGTGAGTAAAGCTTCAGTGCAAACC	32	71.6	actaaatctac	11	TCTAGATTGGATCTTGCTGGCGC	23	66	66	132
BARD1_e01''	GGGTTCCCTAAGGGTTGGA	19	cgctactactattagtagaattgatg	26	TTTCTGGGGGCGGCAGAATCTTT	23	72.3	TCAAATCTTCCGTTTCCTCCTTCCG	25	71.3	aatggtcaaactaaatctac	20	TCTAGATTGGATCTTGCTGGCGC	23	68	68	136
BARD1_e05	GGGTTCCCTAAGGGTTGGA	19	cgctactactattagtaga	19	GCGACATACCTTCTGTTGAATACCTTTTACAA	32	70.5	AATGGAAGTGATCCAAATGTTAAAGACCATGC	32	71.5	tcaaactaaatctac	15	TCTAGATTGGATCTTGCTGGCGC	23	70	70	140
ctrl_3 [chr17]	GGGTTCCCTAAGGGTTGGA	19	cgctactactattagtagaattgatg	26	TCCCTGCGCCATTGAGGTCTATAAAAT	27	70.6	TATAGAGAAAGTTGATTACCCCCGGGATG	29	70.9	aatggtcaaactaaatctac	20	TCTAGATTGGATCTTGCTGGCGC	23	72	72	144
BARD1_e04.2	GGGTTCCCTAAGGGTTGGA	19	cgctactactattagtagaattgatgcc	28	GAGTGATGTCTAGTCCCTCAGCAATGA	27	70.9	AGCTGTTGCCCAATATGGCTGTGAAA	26	71.6	tatctaatggtcaaactaaatctac	25	TCTAGATTGGATCTTGCTGGCGC	23	74	74	148
ARID1A_e03	GGGTTCCCTAAGGGTTGGA	19	cgctactactattagtagaattgatgccacctttt	35	CAGTCTCAACCACCACAGCTCC	22	71.0	AGTCCTCTCAGCCTCCATACTCCC	24	70.7	aatgtatctaatggtcaaactaaatctac	29	TCTAGATTGGATCTTGCTGGCGC	23	76	76	152
ARID1A_i01	GGGTTCCCTAAGGGTTGGA	19	cgctactactattagtagaattgatgccaccttt	34	AGCTTCCACTTCTGTGGACTGCTTC	25	70.5	AGGTGTGTGGTAGTAGTCTAGGTGAGGG	28	70.9	tgtatctaatggtcaaactaaatctac	27	TCTAGATTGGATCTTGCTGGCGC	23	78	78	156
ARID1A_e16	GGGTTCCCTAAGGGTTGGA	19	cgctactactattagtagaattgatgccaccttttcagct	40	CCACAGCCGAATCTCATGCCT	21	71.1	TCCAACCCAGACTCGGGGATG	21	72.6	tttgcgaaatgtatctaatggtcaaactaaatctac	36	TCTAGATTGGATCTTGCTGGCGC	23	80	80	160

PSS – primer specific sequence, SS – stuffer sequence, TSS - target specific sequence, HPL – half probe length

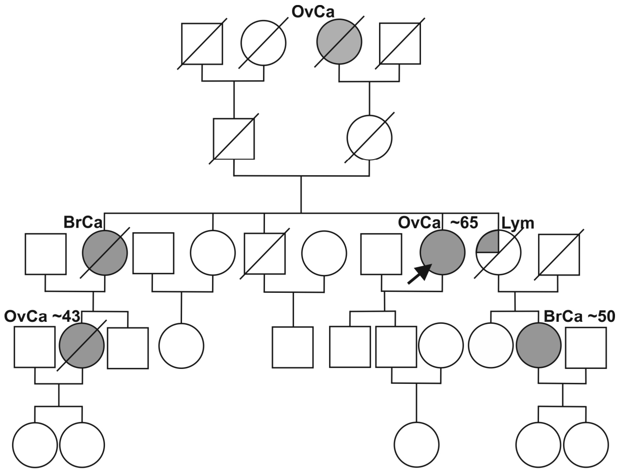

Supplementary Figure S1 A pedigree of proband #53 (carrier of the c.1690C>T mutation), who is indicated by the arrow. The family members with breast or ovarian cancer are shown in grey. The circle that is ¼ grey indicates a different cancer type. OvCa – ovarian cancer, BrCa – breast cancer, Lym – lymphoma. The numbers near the patient's cancer type indicate the age of diagnosis.
